# Landscape analysis of pregnancy exposure registries in low- and middle-income countries: a scoping review

**DOI:** 10.1136/bmjopen-2024-097198

**Published:** 2025-10-29

**Authors:** Niranjan Bhat, Sophie Knudson, Rahmeh AbuShweimeh, Hilma Nakambale, Jessica Mooney, Nancy Salts, Ushma C Mehta, Esperança Sevene, Deshayne Fell, Smaragda Lamprianou, Shanthi N Pal, Andy Stergachis

**Affiliations:** 1 Center for Vaccine Innovation and Access, Program for Appropriate Technology in Health, Seattle, Washington, USA; 2 School of Public Health, University of Washington, Seattle, Washington, USA; 3 Department of Global Health, School of Public Health, University of Washington, Seattle, Washington, USA; 4 Division of Clinical Pharmacology, Department of Medicine, University of Cape Town, Rondebosch, South Africa; 5 Centro de Investigacao em Saude de Manhica, Manhica, Maputo, Mozambique; 6 Department of Physiological Sciences, Faculty of Medicine, Universidade Eduardo Mondlane, Maputo, Mozambique; 7 School of Epidemiology and Public Health, University of Ottawa, Ottawa, Ontario, Canada; 8 Department of Pediatrics, Children’s Hospital of Eastern Ontario, Ottawa, Ontario, Canada; 9 Regulation and Prequalification Department, World Health Organization, Geneva, Switzerland; 10 Department of Pharmacy, University of Washington School of Public Health, Seattle, Washington, USA; 11 Department of Pharmacy, School of Pharmacy, University of Washington, Seattle, Washington, USA

**Keywords:** Pregnancy, Vaccination, REGISTRIES, Drug Utilization, Safety

## Abstract

**Abstract:**

**Introduction:**

Drug and vaccine safety information relevant to pregnant individuals is typically insufficient, especially so for persons living in low- and middle-income countries (LMICs). Pregnancy exposure registries (PERs) and similar systems are used to monitor medical products safety. A better understanding of the landscape of PERs in LMICs can support medicines regulatory system strengthening and preparation for new vaccine and drug introductions.

**Objectives:**

To identify PERs and related health data collection platforms in LMICs that systematically record pregnancy exposures to medical products and pregnancy outcomes to inform how future efforts, such as new vaccine introductions and treatment programmes, can better support maternal populations in these countries.

**Design:**

Scoping review based on methodology outlined in the Joanna Briggs Institute manual for scoping reviews.

**Data sources:**

Electronic search of Medline/PubMed, Embase, CINAHL and Global Index Medicus in June 2022, and key informants via online survey in July 2022 and interviews.

**Eligibility criteria:**

Eligible resources included registries, surveillance systems and databases that collect information on exposures to medical products during pregnancy and on subsequent maternal, perinatal and neonatal outcomes in populations located entirely or partially in LMICs. Eligible records were published from January 2000 through June 2022.

**Data extraction and synthesis:**

Search results were screened and data extracted using a standardised form by two independent reviewers. Instances of discordance were resolved by a third reviewer. Identified systems were categorised by resource type.

**Results:**

A total of 7515 records from electronic searches were screened, with 396 selected for full-text review and 47 additional records obtained from other sources. From these, 45 data collection systems located in African, Asian and Latin American LMICs were identified, with 36 currently in operation. These resources were grouped into six categories based on structure and approach and summarised according to key features, strengths and weaknesses.

**Conclusions:**

This scoping review identified several resources in LMICs dedicated to drug and vaccine safety in pregnancy, but findings indicate that more investment will be needed to ensure such efforts are widespread and sustainable. Understanding the current landscape of such resources in these settings is an important step towards improving safe, world-wide access to life-saving interventions for pregnant populations.

**Trial registration number:**

The protocol for this review has been registered with Open Science Framework (https://doi.org/10.17605/OSF.IO/FU5AT).

**STRENGTHS AND LIMITATIONS OF THIS STUDY**

- This analysis documents pregnancy registries and similar systems in low- and middle-income countries for monitoring the safety of drugs and vaccines.
- This scoping review employed a structured search of the published scientific literature, augmented by a grey literature search, online survey and expert consultations.
- Some registries, particularly those without publications or accessible websites, may nevertheless have been missed in this review.
- Registries were not always thorough in reporting the details of their methods, strengths and limitations in their publications.

## Introduction

 Vaccines and drugs have the potential to reduce mortality and morbidity among pregnant individuals, and their offspring, worldwide. However, sufficient information on the safety of drugs and biologic products used during pregnancy is rarely available at the time of licensure.^1 2^ To account for this, active safety monitoring during the postlicensure phase strengthens their benefit-risk assessments throughout the product life-cycle. While such safety data are critical for clinical and policy decision-making, they are rarely available from low- and middle-income countries (LMICs), which often have differing background rates of obstetric and infant adverse outcomes, prevalent conditions such as malaria and HIV^3 4^ and limited healthcare access.

Pregnancy exposure registries (PERs) are a common type of observational study that systematically collects health information following exposure to medical products during pregnancy, typically after product licensure.^5^ PERs are frequently established in high-income countries (HICs)^6 7^ but are much less common in LMICs, where they typically focus on medicines particularly relevant to the health burden of the populations, such as antiretrovirals and antimalarials.^28^,^10^ New vaccines developed specifically for use by pregnant individuals, such as those for respiratory syncytial virus and Group B *Streptococcus*, have been approved or are anticipated for introduction in LMICs, but prelicensure safety data are primarily from HICs with some exceptions.^11^,^13^ To prepare for new medical products, a better understanding of the presence and characteristics of PERs in LMICs is important.

To address this need, an earlier scoping review was conducted to identify existing perinatal data collection systems in LMICs that could support pharmacovigilance in maternal populations, focusing on eight surveillance systems and platforms that held promise for supporting future introductions.^14^ Building on this work, we adopted a complementary and expanded approach that encompasses a broader variety of systems supporting pregnancy safety monitoring in LMICs, designed to inform how future efforts, such as new vaccine introductions and treatment programmes, can better support maternal populations in LMICs. The resulting scoping review has now been posted online^15^ and is summarised here.

## Methods

The methods and tools for this review have been published.^16^ Briefly, a scoping review was conducted to identify PERs, databases and other systems in LMICs that systematically record exposures to medical products during pregnancy and maternal and infant outcomes. We conducted a systematic search of the scientific and grey literature, supplemented by an online survey and interviews with key informants, as needed. We followed the Joanna Briggs Institute (JBI) manual for scoping reviews, and the search strategy is reported using the Preferred Reporting Items for Systematic reviews and Meta-Analyses extension for Scoping Reviews (PRISMA-ScR) Checklist.^17 18^ This protocol was registered with the Open Science Framework.^19^

### Search strategy and information sources

Using an iterative process, the search strategy used predefined criteria for a search in PubMed incorporating controlled vocabulary and free text, as previously described.^16^ The full search strategies are provided in online supplemental file 1. An independent information specialist peer-reviewed the strategy using thePeer Review of Electronic Search Strategies (PRESS) Checklist.^20^ The strategy was then translated to Embase, CINAHL and WHO’s Global Index Medicus. Reference lists of potentially relevant records and articles were also reviewed. Additionally, a grey literature search was conducted, encompassing a Google Scholar search and a review of relevant websites, including industry and professional organisations, associations and alliances; selected public sector agencies (Ministries of Health, regulatory agencies and pharmacovigilance centres) in LMICs; and selected HIC organisations, academic and other non-governmental groups.

### Study selection and data extraction

Retrieved records were downloaded to EndNote V.9.3.3 (Clarivate) for de-duplication, following which Covidence was used for screening. Each title and abstract were screened by two independent reviewer authors to determine eligibility. Sources available in the databases used were mainly in the English language, although some records in Spanish and Portuguese were captured. Abstracts and full texts for these articles were evaluated through automated translation. Disagreements between reviewers were resolved by a third reviewer. Full-text reviews were conducted by two reviewers then selected records proceeded for data extraction. A PRISMA flow diagram^18^ was constructed to summarise record disposition. Key information was recorded using a pilot-tested data extraction form and entered into an electronic database (Smartsheet).

### Informant survey, interviews and expert consultation

An online survey was distributed to experts to identify additional resources in LMICs that may not have been captured, or to provide additional detail for resources already identified. An online survey instrument was developed and shared by members of the WHO Pharmacovigilance Team with counterparts at the WHO regional offices and then delivered on 7 July 2022, to all members of the WHO Programme for International Drug Monitoring (PIDM),^21^ as these are the national pharmacovigilance authorities for their respective countries, and are therefore expected to be knowledgeable about registries in their countries as operators, stakeholders and/or users of the data. We considered it likely that teams that run safety registries in a country would consult with national pharmacovigilance staff during the establishment or operation of their systems. The survey was also sent to members of the WHO Expert Steering Committee (ESC) on Safety Surveillance in Pregnancy in LMICs. The ESC is a group of experts in maternal immunisation safety who are members of the following WHO advisory committees: Strategic Advisory Group of Experts on Immunization, Advisory Committee on the Safety of Medicinal Products, Global Advisory Committee on Vaccine Safety and Mother and Newborn Information for Tracking Outcomes & Results. ESC members assessed and advised WHO on a number of topics managed by the WHO’s pharmacovigilance team, including this study. Additional experts were identified to receive the survey based on the published and grey literature review as well as personal referrals. Semistructured interviews were conducted when additional information about the registries was required. A multidisciplinary technical working group was established to assist and guide the review, and the protocol and results were reviewed by the ESC. The data extraction form, survey questionnaire and interview guide are provided in online supplemental file 1.

### Data analysis

Identified resources were summarised in tables according to relevant characteristics, and the selected PERs were further evaluated based on additional questions, such as their strengths, weaknesses, ability to add new interventions and ability to combine data with other systems. Criteria to determine whether the described system could be utilised or adapted for future PERs include whether the system was currently operational, designed with flexibility to accommodate new vaccines or drugs, capable of timely identification of adverse events, employ electronic or mobile tools for data collection and reporting, incorporate mechanisms for follow-up and reminders or had prior use in postmarketing safety surveillance. The ease with which new exposures could be added could also vary based on the complexity of the exposure, such as if multiple doses are given, or if the drug or vaccine can be administered at different locations, or if additional verifications are needed. Geographic coverage was assessed using maps.

### Patient and public involvement

Patients were not involved in this scoping review.

## Results

### Literature search results

**Figure 1:**
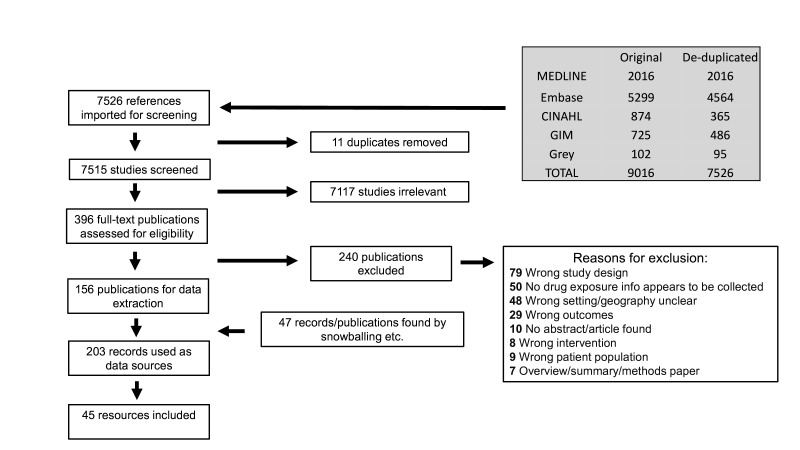
Preferred Reporting Items for Systematic Reviews and Meta-Analyses (PRISMA) flow diagram of records screened and included.^18^

A total of 9016 records were identified in our search; 7515 records after de-duplication were imported for title and abstract screening and 396 were selected for full-text review (figure 1). Of these, 156 met criteria for data extraction. Eighteen individuals responded to the survey, yielding 21 potential resources. After excluding duplicates and negative responses, these results were incorporated into the total. Twelve research studies that were stand-alone, time-limited analyses of retrospectively collected clinical data to evaluate maternal and/or infant outcomes following exposure to drugs or vaccines during pregnancy did not meet eligibility and were not included in the analysis.^22^,^33^ An additional 47 records, publications and other sources were identified through reference lists, websites and informant interviews, resulting in a total of 203 records with relevant information. These records included multiple papers or other sources that referred to a single resource (PER or similar data collection system), as well as individual papers that described multiple resources. The 203 records yielded 45 relevant systems that met our criteria for inclusion.

### Resource categorisation and geographic distribution

**Figure 2:**
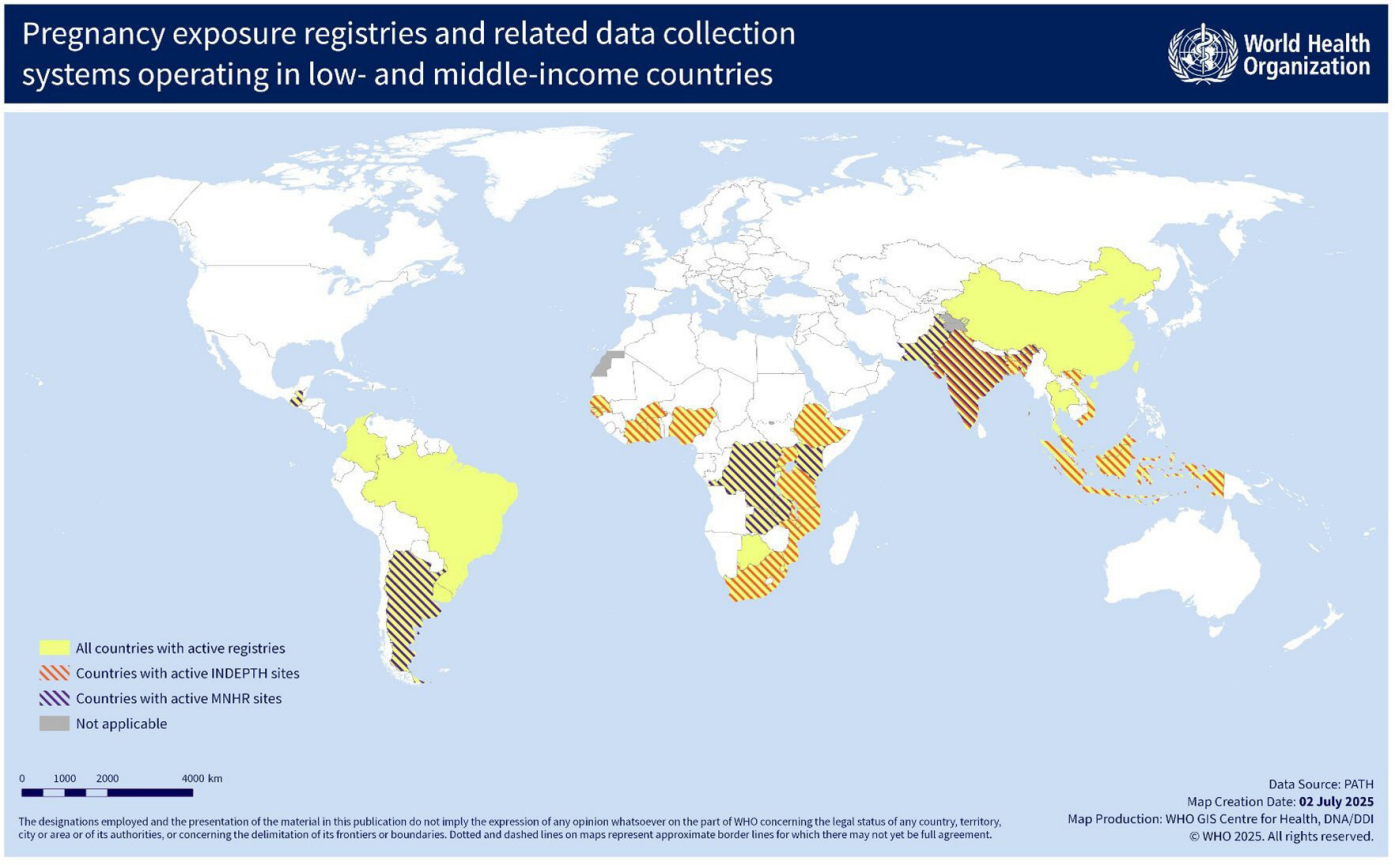
Low- and middle-income countries with active pregnancy exposure registries and related data collection systems.

Resources were grouped into categories based on broad characteristics, as summarised in table 1. These systems were distributed across several LMICs, as demonstrated in figure 2. More than one resource was identified in some countries; conversely, some resources were found to operate in multiple countries. The 36 resources that were determined to be currently active are summarised in online supplemental table S1 and are described in further detail in the following sections according to the resource categories in which they were grouped.

**Table 1:** Categorisation of the pregnancy exposure registries and other resources identified through study methods

Resource category	Brief description	Number of resources (number currently active)
Pregnancy exposure registries	Self-designated pregnancy exposure registries (PERs) with prospective enrolment and a stated aim to record exposures and outcomes	11 (7)
Health and demographic surveillance systems and other observational cohorts	Population-based cohorts with prospective collection of clinical and epidemiologic data	7 (7)
Outcomes-based registries	Registries that focus on outcomes, such as congenital anomalies	7 (7)
Maternal condition-based registries	Registries that enrol pregnant women with specific underlying health conditions	6 (3)
Manufacturer registries	Registries established by a drug or vaccine manufacturer, often for regulatory purposes	8 (6)
Electronic medical record databases and clinical software platforms	Electronic platforms that prospectively record clinical information within a healthcare institution or system	6 (6)
Total		45 (36)

### Pregnancy exposure registries

Resources included in this category met the conventional definition of PERs in that they were prospective observational cohorts focused on the enrolment and follow-up of pregnant women who have received one or more specific drug(s) or vaccine(s) of interest.^5^ Enrolment typically occurs before exposure or at least before any pregnancy outcomes are known. Data are systematically collected on all exposures and outcomes for the woman and child. An unexposed or non-pregnant population may be enroled for comparison, allowing calculation of risk for specific health events.

The majority of PERs in LMICs are currently active and located in sub-Saharan Africa. Many PERs have been in operation for fewer than 5 years with the exception of the Western Cape Pregnancy Exposure Registry (now a contributing site to UBOMI BUHLE).^34 35^ Nine of the PERs are funded through public or donor sources and are run by academic or non-governmental organisations.

Most PERs focus on antiretroviral drugs, with a minority assessing antimalarials, COVID-19 therapeutics or vaccines. In some instances, products of interest being monitored are not fixed; for example, one registry, Malaria in Mothers and Babies Pregnancy Exposure Registry,^36 37^ added COVID-19 vaccinations during the pandemic. Sites of operation extend from single hospitals to multinational networks, and sample sizes range from less than 500 to more than 25 000 participants accumulated over time. As seen in online supplemental table S1, some registries enrol a comparison group such as non-pregnant women of childbearing age, while others enrol only pregnant women, but follow participants exposed and unexposed to the interventions of interest.

The amount of detail provided in the available publications and other reports regarding which outcomes were monitored varied; for instance, conditions such as preterm birth or congenital malformations were not always precisely defined or classified. Most PERs only follow the infants through the neonatal period, with a focus on identifying congenital anomalies, but a small number follow through 1 year or more to follow growth and development or detect late-appearing congenital anomalies.

PERs were among the stronger study designs identified in this scoping review. Key features include systematic, prospective data collection; a focus on pregnant populations and an emphasis on specific exposures of interest. Enrolment before outcomes are known avoids the risk of recall and reporting biases, allows for systematic history review and permits standardisation of methods such as gestational dating and the dose and timing of exposures. The availability of both numerator and denominator data allows calculations of event rates and disease incidence, and comparator groups allow for estimation of risk.

Limitations of PERs include voluntary enrolment, which may bias data towards high-risk pregnancies, and selection bias if refusals are high and participating women have different characteristics from those who do not. Moreover, abnormal outcomes are more likely to be reported than normal outcomes. Limiting enrolment to those attending antenatal care may bias results and diminish the generalisability in some settings. Late disclosure of pregnancy and late initiation of antenatal care limit information regarding the first trimester of pregnancy, gestational age dating and early pregnancy loss. Finally, home births and migration increase loss to follow-up, which may bias results.

### Health and demographic surveillance systems and other Observational cohorts

Several population-based observational cohorts identified in this analysis engaged in maternal pharmacovigilance, but do not meet the strict definition of a PER. These cohorts are located in countries throughout the LMIC regions, and all are currently active. Most are designated as health and demographic surveillance systems (HDSS), indicating that they collect demographic and health events from a geographically defined population, of which pregnant women can be a subset. These sites generally conduct longitudinal surveillance, collecting clinical and epidemiological data prospectively at regular intervals, usually focused on overall health rather than specific exposures. Most of these systems may have the ability to conduct pharmacovigilance in pregnant participants, yet this report only includes those that have published on this topic. A majority of the HDSS resources identified in this review are members of INDEPTH (International Network of field sites with continuous Demographic Evaluation of Populations and Their Health) (http://www.indepth-network.org/), a network that currently encompasses 42 independent health research centres and 49 field sites in 19 LMICs.

The Maternal Newborn Health Registry (MNHR) is another large observational cohort with sites in seven LMICs.^38^ The MNHR is a prospective, population-based research registry that collects data to assess trends in pregnancy outcomes and inform research studies within the network.^39^ The MNHR primarily monitors the outcomes of maternal mortality, neonatal mortality and stillbirth. Specific sites in INDEPTH and MNHR were recently evaluated for their capabilities to conduct safety surveillance following immunisation in pregnancy.^40^

The Child Health and Mortality Prevention Surveillance Network (CHAMPS) Pregnancy Surveillance is a cohort study operating at HDSS sites in several LMICs. Established within the infrastructure of the main CHAMPS study (described separately below), this evaluation is modelled after the MNHR and enrols prospectively and retrospectively to monitor for major birth outcomes, including mortality. Two additional observational cohorts focus on maternal pharmacovigilance, including the Shoklo Malaria Research Unit,^41^,^43^ based on the Thai-Myanmar border, and PREPARE,^44^ created in preparation for planned clinical trials of a candidate maternal vaccine against Group B *Streptococcus*.

The resources in this section pay particular attention to maternal outcomes, but also capture major infant outcomes, with a few extending into the first year of life. Major obstetric outcomes (including spontaneous abortion, stillbirth and maternal death) are universally recorded, but some groups capture a larger range of maternal and/or newborn clinical conditions. Most infant outcomes captured in these systems derive from the early neonatal period, including preterm birth, low birth weight and congenital anomalies.

Also included in this category is the International Epidemiology Databases to Evaluate AIDS (IeDEA), an international research and data exchange consortium that combines observational cohort datasets from over 2.2 million people in 44 countries living with and at risk for HIV. This collaboration combines data from HIV programmes to study antiretroviral treatment, including pregnant individuals. The group has published studies from Brazil,^45 46^ Malawi,^47^ South Africa^48^ and the West African region.^49^

### Outcomes-based registries

Outcomes-based registries focus on the capture of specific outcomes, such as congenital malformations. These studies may be open to an entire hospital catchment area, or may target certain groups, such as those receiving antiretrovirals. These systems typically enrol at the time of birth, where exposures are identified from clinical records or via retrospective methods. Some registries involve a single visit while others follow infants for longer periods, and some may include unaffected infants as a comparison group. A single visit with a focus on examination and classification of surface anomalies allows them to efficiently enrol a large sample size, while longer monitoring can identify late-appearing congenital conditions or monitor growth and development. Some registries that only enrol pregnancies resulting in a live birth do not capture all maternal outcomes (including miscarriages and stillbirths). Unaffected mother-infant pairs from the screened population can be compared with affected pairs to assess for risks associated with exposures of interest.

Follow-up care and support to families may be important roles of the registry. In addition, several birth defects surveillance programmes in Africa have joined together to form the sub-Saharan African Congenital Anomalies Network^50^ to provide a forum for technical support and collaboration through resource sharing and workshops.

Most registries identified in sub-Saharan Africa focus on antiretroviral drugs, while many in East Asia and South America assess incidence rates of congenital anomalies in the broader population and do not emphasise particular interventions or exposures. A limited number (eg, in Uganda, Eswatini and Botswana) were established to assess the teratogenic risks of specific antiretroviral medications such as dolutegravir and cabotegravir. In addition, the CHAMPS study is uniquely focused on stillbirths and neonatal deaths in LMICs.^51 52^

### Maternal condition-based registries

Registries in this category monitor the safety of treatments given to pregnant women with underlying health conditions such as epilepsy, HIV or cardiac disease. These registries may emphasise specific infant outcomes due to a known or suspected safety signal associated with an individual drug or class of pharmacologic products. Some also monitor maternal outcomes associated with the condition, such as seizure frequency or worsening heart failure. Such registries are more likely to be prospective, as participants are already being followed. While registries operated by a single clinic or hospital enrol smaller populations, even national or multinational registries generally do not reach large sample sizes. Unexposed pregnant women or exposed non-pregnant women with these conditions may be enroled as comparator groups.

Outside of HIV-specific registries, systems identified in this category are more commonly located in middle-income countries such as India, Brazil and Argentina, which may reflect that physicians in wealthier countries are able to assemble larger patient cohorts in fields such as neurology or cardiology.

### Manufacturer-initiated registries

Registries in this category are usually funded and owned by the manufacturer of the drug or vaccine of interest, and either focus on an individual drug or vaccine, or can monitor all products in the same therapeutic class as the manufacturer’s product. The US Food and Drug Administration and the European Medicines Agency recommend registries for products likely to be used during pregnancy,^53 54^ and these systems are often established to comply with a regulatory requirement. They can contribute to benefit-harm assessments, support product labelling and inform guidance throughout a medical product’s life-cycle. Most manufacturer registries focus on high-income markets,^55^ but participants from other areas of the world may also be included. Those that indicated some recruitment from LMICs have been included in this review. Active surveillance studies and other research efforts intended to answer specific safety questions that are usually classified as Phase 4 studies were not included.

The registries identified are operated by a range of groups, including contract research organisations, academic groups and the manufacturers themselves. Some use passive surveillance and receive sporadic reports of exposures directly from patients or providers, although some may conduct outreach. Instructions on how to enrol are provided in the product literature or company website. The voluntary nature of enrolment may affect completeness, but once a participant is enroled, the registries maintain contact to solicit outcomes. Some registries are designed for a specific scientific question and thus may limit the timeframe or sample size, while others appear to operate indefinitely.

### Electronic health records databases and clinical software platforms

Systems were included in this review if a published study or report relied on an electronic clinical care database or software platform. In these cases, investigators may have developed programming to identify pregnancies, extract data and classify outcomes appropriately. Electronic health records (EHRs) databases and clinical software platforms typically record information prospectively on all patients within a healthcare system.

EHR platforms may be implemented by individual facilities, health systems or sectors, or nationally. By collecting and storing data electronically, databases can be searched and analysed using structured, reproducible methods. Some EHRs may be deployed in targeted facilities, such as maternal and newborn clinics, or pregnant women and their infants may represent a subset of the total covered population. Linking records across time and databases allows identification of large numbers of relevant exposures and outcomes. Identified databases include SmartCare in Zambia, the Baobab Health Antiretroviral Therapy system in Malawi, and the Provincial Health Data Centre system in the Western Cape province of South Africa.

Software platforms, such as DHIS2 (formerly District Health Information Software), comprise another set of systems. DHIS2 is an open-source web-based software platform that collects and analyses data at the population and individual levels, and can be designed for facilities, health systems or national programmes. By the end of 2022, DHIS2 was used in more than 75 LMICs, with 69 countries using DHIS2 at a national scale.^56 57^ DHIS2 can provide for individual patient care and conduct epidemiological analyses.^58^ Data are entered per routine care rather than through studies, and therefore capture multiple aspects of healthcare. Specific health topics such as HIV, tuberculosis or immunisations can be analysed through preconfigured metadata packages. For instance, the Reproductive, Maternal, Newborn, Child and Adolescent Health package can track exposures and outcomes during pregnancy. This review identified one clear example in Palestine where DHIS2 was used to track maternal exposures and outcomes,^59^ but other deployments may exist.

Other open-source clinical software platforms, such as OpenMRS and OpenEMR, could provide similar functionality.^14^ However, no publications or online sources describing their use for monitoring drug safety during pregnancy were found in our search. The Perinatal Information System (Sistema Informatico Perinatal (SIP)) is a free standardised perinatal clinical record run by Pan American Health Organization’s Latin American Center for Perinatology/Women’s Health and Reproductive Health.^60 61^ Facilities throughout Latin America and the Caribbean use this resource to produce reports and combine data across facilities, conducting studies at the regional or national level.^61^,^63^ While no publications related specifically to pharmacovigilance using SIP were identified, the capability appears possible.

One study analysed data from a national insurance claims database in China to assess the safety of medication use in pregnancy. This approach is common in HICs where methods are well developed and coverage is high. While national health insurance is expanding in LMICs^64 65^ this growth has been limited in the lower-resource areas of Africa and Southeast Asia. Research using these databases is limited^66^,^68^ and none have involved maternal pharmacovigilance, but opportunities may arise as health insurance programmes become more widespread.

Table 2 lists key features of each resource type, highlighting the relative advantages and disadvantages of specific designs.

**Table 2:** Key features of pregnancy exposure registries and related data collection systems, by resource type

Type of resource	Prospective enrolment of pregnant women*	Exposure ascertainment†	Maternal characteristics ascertainment‡	Maternal and infant outcomes ascertainment	Ability to include a comparison group§	Ability to calculate rates and relative risk	Ability to assess new drugs or vaccines of interest	Complexity and resources requirements
Pregnancy exposure registries	Yes	Yes	Yes	Yes	Yes	Yes	Yes	High
Health and demographic surveillance systems (HDSS) and other observational cohorts	Yes	Yes	Yes	Yes	Yes	Yes	Yes	High
Outcomes-based registries	No	No¶	Yes	Yes	Yes	Sometimes**	Yes	Low
Maternal conditions-based registries	Yes	Yes	Yes	Yes	Yes	No	Yes	Low
Manufacturer registries	Sometimes	Yes	Yes	Yes	Sometimes	Sometimes††	Sometimes	High
EMR databases and clinical software platform with pregnancy exposure module	Yes‡‡	Yes	Yes	Yes§§	Yes	Yes¶¶	Yes	Low

* Enrolment of the pregnant woman before the outcome of pregnancy is known. Can be before or after the exposure has occurred.

† Vaccine and drug type, dose, frequency, duration, timing in relation to gestation.

‡ Can include relevant demographic information, concomitant illnesses and medications, and reproductive history.

§ Comparison group may be unexposed pregnant participants, exposed non-pregnant participants, or unexposed non-pregnant participants.

¶ May be mitigated if routine clinical data can be accessed and are detailed and prospectively entered into medical records and if standardised terminology is used.

** Yes, if surveillance captures all deliveries or other relevant denominator in a population. Typically does not include spontaneous abortions.

†† Can calculate an incidence rate among a population of exposed; cannot calculate a relative risk unless a comparison group is enroled.

‡‡ Yes, if routine clinical data are detailed and prospectively entered into medical records and if standardised terminology is used.

§§ Can be done through record linkage.

¶¶ If health system covers an entire population.

## Discussion

This scoping review identified 45 PERs and related systems pertinent to maternal pharmacovigilance in LMICs, 36 of which remain active. These systems cover a range of approaches and areas of focus ranging from conventional PERs to electronic health information systems.

Of these resource types, traditional PERs remain essential for monitoring safety in pregnancy by employing rigorous designs and methods and including comparison groups to calculate risk. However, they are resource-intensive, which can limit their duration, size and/or geographic coverage. The majority of registries identified focus on antiretrovirals, reflecting the importance of these interventions in many LMIC populations, particularly during pregnancy. Currently active PERs might be adapted to include new exposures of interest, as had been done for COVID-19 vaccines.^36^

HDSS cohorts are widespread throughout LMICs and have a long history of operation.^69^ While most are centred around basic vital statistics reporting, some have incorporated additional activities and investment to identify pregnancies early, perform gestational dating, ensure accurate exposure reporting and capture maternal and infant outcomes more completely. Many sites include community surveillance, and thus can monitor pregnancies and deliveries outside health facilities. These systems can often establish comparator populations, determine background rates and calculate relative risk estimates. Ultimately, these sites require substantial resources and community engagement but provide the expertise and infrastructure necessary to conduct pharmacovigilance studies in pregnant populations.

Outcomes-based registries are ideal to detect uncommon outcomes but are often streamlined and follow participants only when outcomes of interest are detected. Efforts may be required to avoid incompleteness or recall bias due to retrospective data collection, and they might not always capture early obstetric events. Electronic medical record systems represent another avenue that integrates activities within routine clinical care. However, quality relies on training and oversight, and analyses benefit from standardised terminologies and methods. The addition of maternal pharmacovigilance could require a comparatively small incremental investment.

This scoping review employed a structured comprehensive search of published scientific literature, but was subject to some limitations. A number of registries, particularly in LMICs, may not have published their data and may have a minimal online presence. In addition, the databases selected for our search may not have contained all relevant publications. At the time of designing our search strategy, we considered that additional databases such as the Web of Science and Scopus would likely overlap considerably with our chosen data sources. However, these repositories might include some unique references and could be considered for future reviews. Even when publications or websites are available, registries were not always thorough in reporting the details of their methods, strengths and limitations. Our literature search was augmented by grey literature, surveys, expert consultations and outreach to a limited number of registry informants to account for these issues; however, some resources may still have been missed or incompletely characterised.

This landscape identified several resources dedicated to drug and vaccine safety in pregnancy in LMICs, but it is clear that more investment is needed to ensure such efforts are inclusive and sustainable. Most of the registries identified could be strengthened or modified to incorporate new exposures, if adequate resources are provided. The inclusion of desirable features, such as early enrolment, prospective data collection, accurate gestational dating, capture of maternal events and enumerated populations to generate denominator-based rates, should be introduced or reinforced wherever possible. However, specific approaches must be matched to their local context, taking into account the questions to be answered, funding available and existing infrastructure. Registries with flexible design might combine analyses across regions, but such efforts will require a more granular understanding of structure, data variables and methodologic approaches. One consideration for such an undertaking would be whether the registries use standardised case definitions such as those developed by the Brighton Collaboration and the Global Alignment of Immunization Safety Assessment in Pregnancy (GAIA) initiative to promote harmonisation, enhance reproducibility and ensure scientifically robust assessments of safety. Other design elements, such as the duration of follow-up and the degree of detail in describing the exposure would need to be matched. In the situation where multiple registries are operating in the same region, procedures would need to be established to identify individuals with overlapping participation, perhaps by developing data queries that search for records with matching elements such as name, date of birth and contact information. Ultimately, improving the safe access of pregnant populations in LMICs to life-saving drugs and vaccines must begin with a clear understanding of where the greatest needs are in order to develop effective strategies to safeguard against possible harms that may be associated with their use.

## Supplementary material

10.1136/bmjopen-2024-097198 online supplemental file 1

10.1136/bmjopen-2024-097198 online supplemental file 2

## Data Availability

All data relevant to the study are included in the article or uploaded as supplementary information.
