# Supplementary material for: Landscape analysis of pregnancy exposure registries in low- and middle-income countries: a scoping review
Source: BMJ Open. 2025 Oct 29;15(10):e097198. doi: 10.1136/bmjopen-2024-097198 (PMC12574391; doi:10.1136/bmjopen-2024-097198)
Supplement: online supplemental file 1 [file bmjopen-15-10-s001.docx]

**Supplemental Material for:**

Bhat, et al. Landscape Analysis of Pregnancy Exposure Registries in Low- and Middle-Income Countries: a Scoping Review

**Search Strategy by Database – page 1**

**Data Extraction Form – page 30**

**Key Informant Survey and Interview Form – page 40**

**Search strategies by database**

PubMed

| Set # | Query | Results |
| --- | --- | --- |
| 132 | #130 NOT #131 | 2,016 |
| 131 | address[Publication Type] OR autobiography[Publication Type] OR bibliography[Publication Type] OR biography[Publication Type] OR comment[Publication Type] OR dictionary[Publication Type] OR directory[Publication Type] OR editorial[Publication Type] OR "expression of concern"[Publication Type] OR festschrift[Publication Type] OR historical article[Publication Type] OR interactive tutorial[Publication Type] OR lecture[Publication Type] OR news[Publication Type] OR newspaper article[Publication Type] OR portrait[Publication Type] OR video-audio media[Publication Type] OR webcast[Publication Type] | 2,050,653 |
| 130 | #128 NOT #129 | 2,048 |
| 129 | Animals[mesh] NOT Humans[mesh] | 5,016,862 |
| 128 | #126 AND #127 | 2,051 |
| 127 | ("2000"[Date - Publication] : "3000"[Date - Publication]) | 20,721,844 |
| 126 | #61 AND #125 | 2,597 |
| 125 | #62 OR #63 OR #64 OR #65 OR #66 OR #67 OR #68 OR #69 OR #70 OR #71 OR #72 OR #73 OR #74 OR #75 OR #76 OR #77 OR #78 OR #79 OR #80 OR #81 OR #82 OR #83 OR #84 OR #85 OR #86 OR #87 OR #88 OR #89 OR #90 OR #91 OR #92 OR #93 OR #94 OR #95 OR #96 OR #97 OR #98 OR #99 OR #100 OR #101 OR #102 OR #103 OR #104 OR #105 OR #106 OR #107 OR #108 OR #109 OR #110 OR #111 OR #112 OR #113 OR #114 OR #115 OR #116 OR #117 OR #118 OR #119 OR #120 OR #121 OR #122 OR #123 OR #124 | 7,704,889 |
| 124 | MNCH[Other Term] AND (maternal*[Other Term] OR newborn*[Other Term] OR child*[Other Term]) | 11 |
| 123 | MNCH[Title] AND (maternal*[Title] OR newborn*[Title] OR child*[Title]) | 22 |
| 122 | "Maternal Health"[mesh] | 2,142 |
| 121 | "Child Health"[mesh] | 4,447 |
| 120 | "Infant Health"[mesh] | 1,175 |
| 119 | (birth[Other Term] OR births[Other Term]) AND defect*[Other Term] | 1,029 |
| 118 | (birth[Title] OR births[Title]) AND defect*[Title] | 2,671 |
| 117 | (birth[Other Term] OR births[Other Term] OR matern*[Other Term] OR neonat*[Other Term] OR neo-nat*[Other Term] OR perinatal*[Other Term] OR peri-natal*[Other Term] OR peripartum[Other Term] OR "peri-partum"[Other Term] OR postnatal*[Other Term] OR post-natal*[Other Term] OR postpartum[Other Term] OR "post-partum"[Other Term] OR post-birth*[Other Term] OR pregnanc*[Other Term]) AND outcome*[Other Term] | 9,426 |
| 116 | (birth[Title] OR births[Title] OR matern*[Title] OR neonat*[Title] OR neo-nat*[Title] OR perinatal*[Title] OR peri-natal*[Title] OR peripartum[Title] OR "peri-partum"[Title] OR postnatal*[Title] OR post-natal*[Title] OR postpartum[Title] OR "post-partum"[Title] OR post-birth*[Title] OR pregnanc*[Title]) AND outcome*[Title] | 31,066 |
| 115 | Pregnancy Outcome[mesh] | 82,242 |
| 114 | (fetal[OT] OR foetal[OT] OR fetus*[OT] OR foetus*[OT] OR prenatal*[OT] OR pre-natal*[OT]) AND expos*[OT] | 2,033 |
| 113 | (fetal[Title] OR foetal[Title] OR fetus*[Title] OR foetus*[Title] OR prenatal*[Title] OR pre-natal*[Title]) AND expos*[Title] | 10,858 |
| 112 | "Prenatal Exposure Delayed Effects"[mesh] | 33,016 |
| 111 | (fetal[Other Term] OR foetal[Other Term] OR fetus*[Other Term] OR foetus*[Other Term]) AND develop*[Other Term] | 2,531 |
| 110 | (fetal[Title] OR foetal[Title] OR fetus*[Title] OR foetus*[Title]) AND develop*[Title] | 8,734 |
| 109 | "Fetal Development"[mesh:noexp] | 9,246 |
| 108 | antiretroviral*[Other Term] OR anti-retroviral*[Other Term] OR "anti-HIV"[Other Term] OR "anti-AIDS"[Other Term] OR "AIDS drug"[Other Term] OR "AIDS drugs"[Other Term] | 7,307 |
| 107 | (antiretroviral*[Title] OR anti-retroviral*[Title] OR "anti-HIV"[Title] OR "anti-AIDS"[Title] OR "AIDS drug"[Title] OR "AIDS drugs"[Title]) | 29,826 |
| 106 | "Anti-Retroviral Agents"[mesh] | 69,532 |
| 105 | "Zika Virus Infection/prevention and control"[MeSH] | 1,096 |
| 104 | "Streptococcal Infections/prevention and control"[MeSH:noexp] | 2,944 |
| 103 | "Respiratory Syncytial Virus Infections/prevention and control"[MeSH Terms] | 1,832 |
| 102 | "Hepatitis E/prevention and control"[MeSH Terms] | 346 |
| 101 | "Cytomegalovirus Infections/prevention and control"[MeSH Terms] | 3,130 |
| 100 | antimalarial*[Other Term] OR anti-malarial*[Other Term] | 2,577 |
| 99 | antimalarial*[Title] OR anti-malarial*[Title] | 8,562 |
| 98 | Antimalarials[mesh] | 28,226 |
| 97 | "Malaria/prevention and control"[MeSH Terms] | 17,528 |
| 96 | (maternal[Other Term] OR pregnan*[Other Term]) AND (immunisation*[Other Term] OR immunization*[Other Term]) | 754 |
| 95 | (maternal[Title] OR pregnan*[Title]) AND (immunisation*[Title] OR immunization*[Title]) | 1,153 |
| 94 | (antibod*[Other Term] OR anti-bod*[Other Term]) AND transfer*[Other Term] | 338 |
| 93 | (antibod*[Title] OR anti-bod*[Title]) AND transfer*[Title] | 1,620 |
| 92 | immunit*[Other Term] AND maternally-acqui*[Other Term] | 8 |
| 91 | immunit*[Title] AND maternally-acqui*[Title] | 8 |
| 90 | immunit*[Other Term] AND transfer*[Other Term] | 161 |
| 89 | immunit*[Title] AND transfer*[Title] | 882 |
| 88 | "Immunity, Maternally-Acquired"[mesh] | 5,842 |
| 87 | "Immunization Programs"[mesh:noexp] | 12,435 |
| 86 | Immunization[mesh:noexp] | 144,668 |
| 85 | vaccin*[ti] OR vaccin*[ot] | 220,223 |
| 84 | "Viral Vaccines"[mesh] | 132,729 |
| 83 | "Vaccines, Live, Unattenuated"[mesh] | 82 |
| 82 | "Vaccines, Synthetic"[mesh] | 31,589 |
| 81 | "Vaccines, Inactivated"[mesh:noexp] | 6,082 |
| 80 | "Vaccines, Combined"[mesh] | 10,562 |
| 79 | "Vaccines, Attenuated"[mesh] | 12,810 |
| 78 | "Protozoan Vaccines"[mesh] | 6,894 |
| 77 | "Bacterial Vaccines"[mesh] | 72,365 |
| 76 | Vaccines[mesh:noexp] | 25,746 |
| 75 | Vaccination[mesh] | 100,718 |
| 74 | GAIA[Text Word] AND (alignment[Text Word] OR immunisation[Text Word] OR immunization[Text Word] OR safety[Text Word] OR pregnan*[Text Word]) | 35 |
| 73 | "Global Alignment of Immunization Safety Assessment in Pregnancy"[tw] - Schema: all | 0 |
| 72 | AEFI[Other Term] AND adverse[Other Term] | 68 |
| 71 | AEFI[Title] AND adverse[Title] | 35 |
| 70 | adverse effect[Other Term] OR adverse effects[Other Term] OR adverse reaction[Other Term] OR adverse reactions[Other Term] OR adverse event[Other Term] OR adverse events[Other Term] OR adverse outcome[Other Term] OR adverse outcomes[Other Term] | 17,729 |
| 69 | adverse effect[Title] OR adverse effects[Title] OR adverse reaction[Title] OR adverse reactions[Title] OR adverse event[Title] OR adverse events[Title] OR adverse outcome[Title] OR adverse outcomes[Title] | 31,848 |
| 68 | safe[Other Term] OR safety[Other Term] OR side effect[Other Term] OR side effects[Other Term] OR undesirable effect[Other Term] OR undesirable effects[Other Term] OR treatment emergent[Other Term] OR tolerability[Other Term] OR toxicity[Other Term] OR adrs[Other Term] | 98,171 |
| 67 | safe[Title] OR safety[Title] OR side effect[Title] OR side effects[Title] OR undesirable effect[Title] OR undesirable effects[Title] OR treatment emergent[Title] OR tolerability[Title] OR toxicity[Title] OR adrs[Title] | 296,710 |
| 66 | ("adverse effects"[MeSH Subheading]) OR (Complications[MeSH Subheading]) OR ("drug effects"[MeSH Subheading]) | 6,963,724 |
| 65 | (drug[Title] OR drugs[Title] OR medicine[Title] OR medicines[Title] OR medication*[Title] OR pharmaceutical*[Title] OR pharma-ceutical*[Title]) AND expos*[Title] | 4,387 |
| 64 | "Drug-Related Side Effects and Adverse Reactions"[MeSH Terms] | 127,437 |
| 63 | (global[Title] OR international*[Title] OR world*[Title]) AND health*[Title] | 27,778 |
| 62 | Global Health[mesh] | 53,845 |
| 61 | #56 AND #60 | 8,450 |
| 60 | #57 OR #58 OR #59 | 1,104,126 |
| 59 | "high burden country"[Other Term] OR "high burden countries"[Other Term] OR "high-burden country"[Other Term] OR "high-burden countries"[Other Term] OR "countdown country"[Other Term] OR "countdown countries"[Other Term] | 10 |
| 58 | "high burden country"[Title/Abstract] OR "high burden countries"[Title/Abstract] OR "high-burden country"[Title/Abstract] OR "high-burden countries"[Title/Abstract] OR "countdown country"[Title/Abstract] OR "countdown countries"[Title/Abstract] | 633 |
| 57 | afghanistan[Text Word] OR albania[Text Word] OR algeria[Text Word] OR "american samoa"[Text Word] OR angola[Text Word] OR antigua[Text Word] OR barbuda[Text Word] OR argentina[Text Word] OR armenia[Text Word] OR armenian[Text Word] OR aruba[Text Word] OR azerbaijan[Text Word] OR bahrain[Text Word] OR bangladesh[Text Word] OR barbados[Text Word] OR belarus[Text Word] OR byelarus[Text Word] OR belorussia[Text Word] OR byelorussian[Text Word] OR belize[Text Word] OR "british honduras"[Text Word] OR benin[Text Word] OR dahomey[Text Word] OR bhutan[Text Word] OR bolivia[Text Word] OR "bosnia herzegovina"[Text Word] OR bosnia[Text Word] OR herzegovina[Text Word] OR botswana[Text Word] OR bechuanaland[Text Word] OR brazil[Text Word] OR brasil[Text Word] OR bulgaria[Text Word] OR "burkina faso"[Text Word] OR "burkina fasso"[Text Word] OR "upper volta"[Text Word] OR burundi[Text Word] OR urundi[Text Word] OR "cabo verde"[Text Word] OR "cape verde"[Text Word] OR cambodia[Text Word] OR kampuchea[Text Word] OR "khmer republic"[Text Word] OR cameroon[Text Word] OR cameron[Text Word] OR cameroun[Text Word] OR "central african republic"[Text Word] OR "ubangi shari"[Text Word] OR chad[Text Word] OR chile[Text Word] OR china[Text Word] OR colombia[Text Word] OR comoros[Text Word] OR "comoro islands"[Text Word] OR "iles comores"[Text Word] OR mayotte[Text Word] OR "democratic republic of the congo"[Text Word] OR "democratic republic congo"[Text Word] OR congo[Text Word] OR zaire[Text Word] OR "costa rica"[Text Word] OR "cote d’ivoire"[Text Word] OR "cote d’ ivoire"[Text Word] OR "cote divoire"[Text Word] OR "cote d ivoire"[Text Word] OR "ivory coast"[Text Word] OR croatia[Text Word] OR cuba[Text Word] OR cyprus[Text Word] OR "czech republic"[Text Word] OR czechoslovakia[Text Word] OR djibouti[Text Word] OR "french somaliland"[Text Word] OR dominica[Text Word] OR "dominican republic"[Text Word] OR ecuador[Text Word] OR egypt[Text Word] OR "united arab republic"[Text Word] OR "el salvador"[Text Word] OR "equatorial guinea"[Text Word] OR "spanish guinea"[Text Word] OR eritrea[Text Word] OR estonia[Text Word] OR eswatini[Text Word] OR swaziland[Text Word] OR ethiopia[Text Word] OR fiji[Text Word] OR gabon[Text Word] OR "gabonese republic"[Text Word] OR gambia[Text Word] OR "georgia (republic)"[Text Word] OR georgian[Text Word] OR ghana[Text Word] OR "gold coast"[Text Word] OR gibraltar[Text Word] OR greece[Text Word] OR grenada[Text Word] OR guam[Text Word] OR guatemala[Text Word] OR guinea[Text Word] OR "guinea bissau"[Text Word] OR guyana[Text Word] OR "british guiana"[Text Word] OR haiti[Text Word] OR hispaniola[Text Word] OR honduras[Text Word] OR hungary[Text Word] OR india[Text Word] OR indonesia[Text Word] OR timor[Text Word] OR iran[Text Word] OR iraq[Text Word] OR "isle of man"[Text Word] OR jamaica[Text Word] OR jordan[Text Word] OR kazakhstan[Text Word] OR kazakh[Text Word] OR kenya[Text Word] OR "democratic people’s republic of korea"[Text Word] OR "republic of korea"[Text Word] OR "north korea"[Text Word] OR "south korea"[Text Word] OR korea[Text Word] OR kosovo[Text Word] OR kyrgyzstan[Text Word] OR kirghizia[Text Word] OR kirgizstan[Text Word] OR "kyrgyz republic"[Text Word] OR kirghiz[Text Word] OR laos[Text Word] OR "lao pdr"[Text Word] OR "lao people's democratic republic"[Text Word] OR latvia[Text Word] OR lebanon[Text Word] OR lebanese republic[Text Word] OR lesotho[Text Word] OR basutoland[Text Word] OR liberia[Text Word] OR libya[Text Word] OR "libyan arab jamahiriya"[Text Word] OR lithuania[Text Word] OR macau[Text Word] OR macao[Text Word] OR republic of "north macedonia"[Text Word] OR macedonia[Text Word] OR madagascar[Text Word] OR "malagasy republic"[Text Word] OR malawi[Text Word] OR nyasaland[Text Word] OR malaysia[Text Word] OR "malay federation"[Text Word] OR "malaya federation"[Text Word] OR maldives[Text Word] OR "indian ocean islands"[Text Word] OR "indian ocean"[Text Word] OR mali[Text Word] OR malta[Text Word] OR micronesia[Text Word] OR "federated states of micronesia"[Text Word] OR kiribati[Text Word] OR "marshall islands"[Text Word] OR nauru[Text Word] OR "northern mariana islands"[Text Word] OR palau[Text Word] OR tuvalu[Text Word] OR mauritania[Text Word] OR mauritius[Text Word] OR mexico[Text Word] OR moldova[Text Word] OR moldovian[Text Word] OR mongolia[Text Word] OR montenegro[Text Word] OR morocco[Text Word] OR ifni[Text Word] OR mozambique[Text Word] OR "portuguese east africa"[Text Word] OR myanmar[Text Word] OR burma[Text Word] OR namibia[Text Word] OR nepal[Text Word] OR "netherlands antilles"[Text Word] OR nicaragua[Text Word] OR niger[Text Word] OR nigeria[Text Word] OR oman[Text Word] OR muscat[Text Word] OR pakistan[Text Word] OR panama[Text Word] OR "papua new guinea"[Text Word] OR "new guinea"[Text Word] OR paraguay[Text Word] OR peru[Text Word] OR philippines[Text Word] OR philipines[Text Word] OR phillipines[Text Word] OR phillippines[Text Word] OR poland[Text Word] OR "polish people's republic"[Text Word] OR portugal[Text Word] OR "portuguese republic"[Text Word] OR "puerto rico"[Text Word] OR romania[Text Word] OR russia[Text Word] OR "russian federation"[Text Word] OR ussr[Text Word] OR "soviet union"[Text Word] OR "union of soviet socialist republics"[Text Word] OR rwanda[Text Word] OR ruanda[Text Word] OR samoa[Text Word] OR "pacific islands"[Text Word] OR polynesia[Text Word] OR "samoan islands"[Text Word] OR "navigator island"[Text Word] OR "navigator islands"[Text Word] OR "sao tome and principe"[Text Word] OR "saudi arabia"[Text Word] OR senegal[Text Word] OR serbia[Text Word] OR seychelles[Text Word] OR "sierra leone"[Text Word] OR slovakia[Text Word] OR "slovak republic"[Text Word] OR slovenia[Text Word] OR melanesia[Text Word] OR "solomon island"[Text Word] OR "solomon islands"[Text Word] OR "norfolk island"[Text Word] OR "norfolk islands"[Text Word] OR somalia[Text Word] OR "south africa"[Text Word] OR "south sudan"[Text Word] OR "sri lanka"[Text Word] OR ceylon[Text Word] OR "saint kitts and nevis"[Text Word] OR "st. kitts and nevis"[Text Word] OR "saint lucia"[Text Word] OR "st. lucia"[Text Word] OR "saint Vincent and the grenadines"[Text Word] OR "saint vincent"[Text Word] OR "st. vincent"[Text Word] OR grenadines[Text Word] OR sudan[Text Word] OR suriname[Text Word] OR surinam[Text Word] OR "dutch guiana"[Text Word] OR "netherlands guiana"[Text Word] OR syria[Text Word] OR "syrian arab republic"[Text Word] OR tajikistan[Text Word] OR tadjikistan[Text Word] OR tadzhikistan[Text Word] OR tadzhik[Text Word] OR tanzania[Text Word] OR tanganyika[Text Word] OR thailand[Text Word] OR siam[Text Word] OR "timor leste"[Text Word] OR "east timor"[Text Word] OR togo[Text Word] OR "togolese republic"[Text Word] OR tonga[Text Word] OR "Trinidad and tobago"[Text Word] OR trinidad[Text Word] OR tobago[Text Word] OR tunisia[Text Word] OR turkey[Text Word] OR turkmenistan[Text Word] OR turkmen[Text Word] OR uganda[Text Word] OR ukraine[Text Word] OR uruguay[Text Word] OR uzbekistan[Text Word] OR uzbek[Text Word] OR vanuatu[Text Word] OR "new hebrides"[Text Word] OR venezuela[Text Word] OR vietnam[Text Word] OR "viet nam"[Text Word] OR "middle east"[Text Word] OR "west bank"[Text Word] OR gaza[Text Word] OR palestine[Text Word] OR yemen[Text Word] OR yugoslavia[Text Word] OR zambia[Text Word] OR zimbabwe[Text Word] OR "northern rhodesia"[Text Word] OR "global south"[Text Word] OR "africa south of the sahara"[Text Word] OR "sub-saharan africa"[Text Word] OR "subsaharan africa"[Text Word] OR "africa, central"[Text Word] OR "central africa"[Text Word] OR "africa, northern"[Text Word] OR "north africa"[Text Word] OR "northern africa"[Text Word] OR magreb[Text Word] OR maghrib[Text Word] OR sahara[Text Word] OR "africa, southern"[Text Word] OR "southern africa"[Text Word] OR "africa, eastern"[Text Word] OR "east africa"[Text Word] OR "eastern africa"[Text Word] OR "africa, western"[Text Word] OR "west africa"[Text Word] OR "western africa"[Text Word] OR "west indies"[Text Word] OR caribbean[Text Word] OR "central america"[Text Word] OR "latin america"[Text Word] OR "south and central america"[Text Word] OR "south america"[Text Word] OR "asia, central"[Text Word] OR "central asia"[Text Word] OR "asia, northern"[Text Word] OR "north asia"[Text Word] OR "northern asia"[Text Word] OR "asia, southeastern"[Text Word] OR "southeastern asia"[Text Word] OR "south eastern asia"[Text Word] OR "southeast asia"[Text Word] OR "south east asia"[Text Word] OR "asia, western"[Text Word] OR "western asia"[Text Word] OR "europe, eastern"[Text Word] OR "east europe"[Text Word] OR "eastern europe"[Text Word] OR "developing country"[Text Word] OR "developing countries"[Text Word] OR "developing nation"[Text Word] OR "developing nations"[Text Word] OR "developing population"[Text Word] OR "developing populations"[Text Word] OR "developing world"[Text Word] OR "less developed country"[Text Word] OR "less developed countries"[Text Word] OR "less developed nation"[Text Word] OR "less developed nations"[Text Word] OR "less developed population"[Text Word] OR "less developed populations"[Text Word] OR "less developed world"[Text Word] OR "lesser developed country"[Text Word] OR "lesser developed countries"[Text Word] OR "lesser developed nation"[Text Word] OR "lesser developed nations"[Text Word] OR "lesser developed population"[Text Word] OR "lesser developed populations"[Text Word] OR "lesser developed world"[Text Word] OR "under developed country"[Text Word] OR "under developed countries"[Text Word] OR "under developed nation"[Text Word] OR "under developed nations"[Text Word] OR "under developed population"[Text Word] OR "under developed populations"[Text Word] OR "under developed world"[Text Word] OR "underdeveloped country"[Text Word] OR "underdeveloped countries"[Text Word] OR "underdeveloped nation"[Text Word] OR "underdeveloped nations"[Text Word] OR "underdeveloped population"[Text Word] OR "underdeveloped populations"[Text Word] OR "underdeveloped world"[Text Word] OR "middle income country"[Text Word] OR "middle income countries"[Text Word] OR "middle income nation"[Text Word] OR "middle income nations"[Text Word] OR "middle income population"[Text Word] OR "middle income populations"[Text Word] OR "low income country"[Text Word] OR "low income countries"[Text Word] OR "low income nation"[Text Word] OR "low income nations"[Text Word] OR "low income population"[Text Word] OR "low income populations"[Text Word] OR "lower income country"[Text Word] OR "lower income countries"[Text Word] OR "lower income nation"[Text Word] OR "lower income nations"[Text Word] OR "lower income population"[Text Word] OR "lower income populations"[Text Word] OR "underserved country"[Text Word] OR "underserved countries"[Text Word] OR "underserved nation"[Text Word] OR "underserved nations"[Text Word] OR "underserved population"[Text Word] OR "underserved populations"[Text Word] OR "underserved world"[Text Word] OR "under served country"[Text Word] OR "under served countries"[Text Word] OR "under served nation"[Text Word] OR "under served nations"[Text Word] OR "under served population"[Text Word] OR "under served populations"[Text Word] OR "under served world"[Text Word] OR "deprived country"[Text Word] OR "deprived countries"[Text Word] OR "deprived nation"[Text Word] OR "deprived nations"[Text Word] OR "deprived population"[Text Word] OR "deprived populations"[Text Word] OR "deprived world"[Text Word] OR "poor country"[Text Word] OR "poor countries"[Text Word] OR "poor nation"[Text Word] OR "poor nations"[Text Word] OR "poor population"[Text Word] OR "poor populations"[Text Word] OR "poor world"[Text Word] OR "poorer country"[Text Word] OR "poorer countries"[Text Word] OR "poorer nation"[Text Word] OR "poorer nations"[Text Word] OR "poorer population"[Text Word] OR "poorer populations"[Text Word] OR "poorer world"[Text Word] OR "developing economy"[Text Word] OR "developing economies"[Text Word] OR "less developed economy"[Text Word] OR "less developed economics"[Text Word] OR "lesser developed economy"[Text Word] OR "lesser developed economies"[Text Word] OR "under developed economy"[Text Word] OR "under developed economies"[Text Word] OR "underdeveloped economy"[Text Word] OR "underdeveloped economies"[Text Word] OR "middle income economy"[Text Word] OR "middle income economies"[Text Word] OR "low income economy"[Text Word] OR "low income economies"[Text Word] OR "lower income economy"[Text Word] OR "lower income economies"[Text Word] OR "low gdp"[Text Word] OR "low gnp"[Text Word] OR "low gross domestic"[Text Word] OR "low gross national"[Text Word] OR "lower gdp"[Text Word] OR "lower gnp"[Text Word] OR "lower gross domestic"[Text Word] OR "lower gross national"[Text Word] OR lmic[Text Word] OR lmics[Text Word] OR "third world"[Text Word] OR "lami country"[Text Word] OR "lami countries"[Text Word] OR "transitional country"[Text Word] OR "transitional economies"[Text Word] OR "emerging economy"[Text Word] OR "emerging economies"[Text Word] OR "emerging nation"[Text Word] OR "emerging nations"[Text Word] | 1,103,822 |
| 56 | #1 OR #55 | 39,866 |
| 55 | #21 AND #54 | 39,859 |
| 54 | #22 OR #23 OR #24 OR #25 OR #26 OR #27 OR #28 OR #29 OR #30 OR #31 OR #32 OR #33 OR #34 OR #35 OR #36 OR #37 OR #38 OR #39 OR #40 OR #41 OR #42 OR #43 OR #44 OR #45 OR #46 OR #47 OR #48 OR #49 OR #50 OR #51 OR #52 OR #53 | 850,027 |
| 53 | "Product Surveillance, Postmarketing/statistics and numerical data"[MeSH Terms] | 2,361 |
| 52 | "Maternal Health Services/statistics and numerical data"[MeSH Terms] | 7,290 |
| 51 | "Maternal Exposure/statistics and numerical data"[MeSH Terms] | 1,123 |
| 50 | pharmacovigilan*[ti] OR pharmaco-vigilan*[ti] OR pharmacovigilan*[ot] OR pharmaco-vigilan*[ot] | 3,751 |
| 49 | "Product Surveillance, Postmarketing"[mesh:noexp] | 7,553 |
| 48 | "data system"[ti] OR "data systems"[ti] OR "information system"[ti] OR "information systems"[ti] OR "data system"[ot] OR "data systems"[ot] OR "information system"[ot] OR "information systems"[ot] | 13,581 |
| 47 | "Health Information Systems"[mesh] | 1,546 |
| 46 | (decision*[ti] AND support*[ti] AND clinical*[ti]) OR (decision*[ot] AND support*[ot] AND clinical*[ot]) | 4,467 |
| 45 | "Decision Support Systems, Clinical"[mesh] | 9,106 |
| 44 | "Databases, Factual"[mesh] | 163,862 |
| 43 | "Databases as Topic"[mesh:noexp] | 9,688 |
| 42 | surveillance*[ti] OR surveillance*[ot] | 61,304 |
| 41 | "Population Surveillance"[mesh] | 74,026 |
| 40 | survey*[ti] OR survey*[ot] | 189,840 |
| 39 | "Health Surveys"[mesh:noexp] | 66,266 |
| 38 | "Health Care Surveys"[MeSH Major Topic] | 10,167 |
| 37 | "Surveys and Questionnaires"[MeSH Major Topic:noexp] | 49,876 |
| 36 | registry[ot] OR registries[ot] OR eregistr*[ot] OR "e-registry"[ot] OR "e-registries"[ot] | 8,509 |
| 35 | registry[ti] OR registries[ti] OR eregistr*[ti] OR "e-registry"[ti] OR "e-registries"[ti] | 34,311 |
| 34 | Registries[mesh:noexp] | 104,749 |
| 33 | (preliminary[ot] OR "pilot project"[ot] OR "pilot projects"[ot]) AND data[ot] | 35 |
| 32 | (preliminary[ti] OR "pilot project"[ti] OR "pilot projects"[ti]) AND data[ti] | 2,947 |
| 31 | "Preliminary Data"[mesh] | 724 |
| 30 | "focus group"[ti] OR "focus groups"[ti] OR "focus group"[ot] OR "focus groups"[ot] | 6,048 |
| 29 | "Focus Groups"[mesh] | 34,384 |
| 28 | databas*[ot] OR "data base"[ot] OR "data bases"[ot] OR databank*[ot] OR "data bank"[ot] OR "data banks"[ot] OR dataset*[ot] OR "data set"[ot] OR "data sets"[ot] | 14,502 |
| 27 | databas*[ti] OR "data base"[ti] OR "data bases"[ti] OR databank*[ti] OR "data bank"[ti] OR "data banks"[ti] OR dataset*[ti] OR "data set"[ti] OR "data sets"[ti] | 55,119 |
| 26 | "Datasets as Topic"[mesh] | 7,252 |
| 25 | data[ot] AND (accumulat*[ot] OR accura*[ot] OR assembl*[ot] OR captur*[ot] OR collect*[ot] OR compil*[ot] OR coordinat*[ot] OR co-ordinat*[ot] OR gather*[ot] OR hub[ot] OR hubs[ot]) | 5,205 |
| 24 | data[ti] AND (accumulat*[ti] OR accura*[ti] OR assembl*[ti] OR captur*[ti] OR collect*[ti] OR compil*[ti] OR coordinat*[ti] OR co-ordinat*[ti] OR gather*[ti] OR hub[ti] OR hubs[ti]) | 11,365 |
| 23 | "Data Accuracy"[mesh] | 3,726 |
| 22 | "Data Collection"[mesh:noexp] | 91,761 |
| 21 | #2 OR #3 OR #4 OR #5 OR #6 OR #7 OR #8 OR #9 OR #10 OR #11 OR #12 OR #13 OR #14 OR #15 OR #16 OR #17 OR #18 OR #19 OR #20 | 1,067,642 |
| 20 | maternal[ot] AND (fetal[ot] OR fetus[ot] OR foetal[ot] OR foetus[ot]) | 3,848 |
| 19 | (maternal*[ot] OR maternity[ot]) AND (care[ot] OR health*[ot] OR service[ot] OR services[ot]) | 11,210 |
| 18 | (maternal*[ot] or maternity[ot]) AND (contact*[ot] or expos*[ot]) | 601 |
| 17 | perinatal*[ot] OR "peri-natal"[ot] OR "peri-natally"[ot] OR peripartum[ot] or "peri-partum"[ot] | 7,552 |
| 16 | prenatal*[ot] OR antenatal*[ot] OR "ante-natal"[ot] OR "ante-natally"[ot] OR antepartum[ot] or "ante-partum"[ot] | 15,862 |
| 15 | maternal[ti] AND (fetal[ti] OR fetus[ti] OR foetal[ti] OR foetus[ti]) | 13,964 |
| 14 | (maternal*[ti] OR maternity[ti]) AND (care[ti] OR health*[ti] OR service[ti] OR services[ti]) | 13,467 |
| 13 | "Maternal Health Services"[mesh:noexp] | 15,617 |
| 12 | (maternal*[ti] or maternity[ti]) AND (contact*[ti] or expos*[ti]) | 3,847 |
| 11 | "Maternal Exposure"[mesh] | 10,689 |
| 10 | perinatal*[ti] OR "peri-natal"[ti] OR "peri-natally"[ti] OR peripartum[ti] or "peri-partum"[ti] | 30,982 |
| 9 | "Perinatal Care"[mesh] | 11,334 |
| 8 | prenatal*[ti] OR antenatal*[ti] OR "ante-natal"[ti] OR "ante-natally"[ti] OR antepartum[ti] or "ante-partum"[ti] | 59,414 |
| 7 | "Prenatal Care"[mesh] | 31,249 |
| 6 | pregnan*[ti] | 252,201 |
| 5 | "Pregnancy Trimesters"[mesh] | 43,874 |
| 4 | "Pregnant Women"[mesh] | 12,334 |
| 3 | "Pregnancy Complications"[mesh] | 460,551 |
| 2 | Pregnancy[mesh] | 970,546 |
| 1 | "pregnancy exposure database" [tw] OR "pregnancy exposure databases" [tw] OR "pregnancy exposure data base" [tw] OR "pregnancy exposure data bases" [tw] OR "pregnancy exposure registry" [tw] OR "pregnancy exposure registries" [tw] | 34 |

Embase

--------------------------------------------------------------------------------

1 ("pregnancy exposure database" or "pregnancy exposure databases" or "pregnancy exposure data base" or "pregnancy exposure data bases" or "pregnancy exposure registry" or "pregnancy exposure registries").tw,kw,kf. (113)

2 exp pregnancy/ (727815)

3 exp pregnancy complication/ (141579)

4 exp named groups by pregnancy/ (130431)

5 pregnan*.ti,kw,kf. (329712)

6 exp prenatal care/ (167165)

7 (prenatal* or antenatal* or "ante-natal" or "ante-natally" or antepartum or "ante-partum").ti,kw,kf. (87488)

8 exp perinatal care/ (65235)

9 (perinatal* or peri-natal* or peripartum or "peri-partum").ti,kw,kf. (46195)

10 maternal exposure/ (3606)

11 ((maternal* or maternity) and (contact* or expos*)).ti,kw,kf. (6357)

12 maternal health service/ (2454)

13 ((maternal* or maternity) and (care or health* or service or services)).ti,kw,kf. (22738)

14 (maternal and (fetal or fetus or foetal or foetus)).ti,kw,kf. (20741)

15 or/2-14 [PREGNANCY] (1035932)

16 *information processing/ (37078)

17 data aggregation/ (374)

18 data accuracy/ (1596)

19 (data and (accumulat* or accura* or assembl* or captur* or collect* or compil* or coordinat* or co-ordinat* or gather* or hub or hubs)).ti,kw,kf. (18259)

20 (databas* or "data base" or "data bases" or databank* or "data bank" or "data banks" or dataset* or "data set" or "data sets").ti,kw,kf. (86633)

21 (focus group or focus groups).ti,kw,kf. (7608)

22 preliminary data/ (31472)

23 ((preliminary or pilot project or pilot projects) and data).ti,kw,kf. (5481)

24 exp register/ (180900)

25 (registry or registries or eregistr* or "e-registry" or "e-registries").ti,kw,kf. (68347)

26 questionnaire/ (814172)

27 *health care survey/ (2606)

28 health survey/ (213987)

29 survey*.ti,kw,kf. (220889)

30 population surveillance/ (117)

31 surveillance*.ti,kw,kf. (82221)

32 data base/ (248533)

33 factual database/ (27912)

34 clinical decision support system/ (4716)

35 (decision* and support* and clinical*).ti,kw,kf. (5904)

36 medical information system/ (22591)

37 ("data system" or "data systems" or "information system" or "information systems").ti,kw,kf. (18222)

38 exp postmarketing surveillance/ (37924)

39 (pharmacovigilan* or pharmaco-vigilan*).ti,kw,kf. (7417)

40 or/16-39 [DATA COLLECTION, REGISTRIES] (1815231)

41 15 and 40 [PREGNANCY - DATA COLLECTION, REGISTRIES] (71373)

42 1 or 41 [PERs, PREGNANCY - DATA COLLECTION, REGISTRIES] (71381)

43 (afghanistan or albania or algeria or american samoa or angola or "antigua and barbuda" or antigua or barbuda or argentina or armenia or armenian or aruba or azerbaijan or bahrain or bangladesh or barbados or republic of belarus or belarus or byelarus or belorussia or byelorussian or belize or british honduras or benin or dahomey or bhutan or bolivia or "bosnia and herzegovina" or bosnia or herzegovina or botswana or bechuanaland or brazil or brasil or bulgaria or burkina faso or burkina fasso or upper volta or burundi or urundi or cabo verde or cape verde or cambodia or kampuchea or khmer republic or cameroon or cameron or cameroun or central african republic or ubangi shari or chad or chile or china or colombia or comoros or comoro islands or iles comores or mayotte or democratic republic of the congo or democratic republic congo or congo or zaire or costa rica or "cote d’ivoire" or "cote d’ ivoire" or cote divoire or cote d ivoire or ivory coast or croatia or cuba or cyprus or czech republic or czechoslovakia or djibouti or french somaliland or dominica or dominican republic or ecuador or egypt or united arab republic or el salvador or equatorial guinea or spanish guinea or eritrea or estonia or eswatini or swaziland or ethiopia or fiji or gabon or gabonese republic or gambia or "georgia (republic)" or georgian or ghana or gold coast or gibraltar or greece or grenada or guam or guatemala or guinea or guinea bissau or guyana or british guiana or haiti or hispaniola or honduras or hungary or india or indonesia or timor or iran or iraq or isle of man or jamaica or jordan or kazakhstan or kazakh or kenya or "democratic people’s republic of korea" or republic of korea or north korea or south korea or korea or kosovo or kyrgyzstan or kirghizia or kirgizstan or kyrgyz republic or kirghiz or laos or lao pdr or "lao people's democratic republic" or latvia or lebanon or lebanese republic or lesotho or basutoland or liberia or libya or libyan arab jamahiriya or lithuania or macau or macao or republic of north macedonia or macedonia or madagascar or malagasy republic or malawi or nyasaland or malaysia or malay federation or malaya federation or maldives or indian ocean islands or indian ocean or mali or malta or micronesia or federated states of micronesia or kiribati or marshall islands or nauru or northern mariana islands or palau or tuvalu or mauritania or mauritius or mexico or moldova or moldovian or mongolia or montenegro or "montenegro (republic)" or morocco or ifni or mozambique or portuguese east africa or myanmar or burma or namibia or nepal or netherlands antilles or nicaragua or niger or nigeria or oman or muscat or pakistan or panama or papua new guinea or new guinea or paraguay or peru or philippines or philipines or phillipines or phillippines or poland or "polish people's republic" or portugal or portuguese republic or puerto rico or romania or russia or russian federation or ussr or soviet union or union of soviet socialist republics or rwanda or ruanda or samoa or pacific islands or polynesia or samoan islands or navigator island or navigator islands or "sao tome and principe" or saudi arabia or senegal or serbia or seychelles or sierra leone or slovakia or slovak republic or slovenia or melanesia or solomon island or solomon islands or norfolk island or norfolk islands or somalia or south africa or south sudan or sri lanka or ceylon or "saint kitts and nevis" or "st. kitts and nevis" or saint lucia or "st. lucia" or "saint vincent and the grenadines" or saint vincent or "st. vincent" or grenadines or sudan or suriname or surinam or dutch guiana or netherlands guiana or syria or syrian arab republic or tajikistan or tadjikistan or tadzhikistan or tadzhik or tanzania or tanganyika or thailand or siam or timor leste or east timor or togo or togolese republic or tonga or "trinidad and tobago" or trinidad or tobago or tunisia or "turkey (republic)" or turkey or turkmenistan or turkmen or uganda or ukraine or uruguay or uzbekistan or uzbek or vanuatu or new hebrides or venezuela or vietnam or viet nam or middle east or west bank or gaza or palestine or yemen or yugoslavia or zambia or zimbabwe or northern rhodesia or global south or africa south of the sahara or "sub saharan africa" or subsaharan africa or africa, central or central africa or africa, northern or north africa or northern africa or magreb or maghrib or sahara or africa, southern or southern africa or africa, eastern or east africa or eastern africa or africa, western or west africa or western africa or west indies or indian ocean islands or caribbean region or caribbean islands or caribbean or central america or latin america or "south and central america" or south america or asia, central or central asia or asia, northern or north asia or northern asia or asia, southeastern or southeastern asia or south eastern asia or southeast asia or south east asia or asia, western or western asia or europe, eastern or east europe or eastern europe or developing country or developing countries or developing nation? or developing population? or developing world or less developed countr* or less developed nation? or less developed population? or less developed world or lesser developed countr* or lesser developed nation? or lesser developed population? or lesser developed world or under developed countr* or under developed nation? or under developed population? or under developed world or underdeveloped countr* or underdeveloped nation? or underdeveloped population? or underdeveloped world or middle income countr* or middle income nation? or middle income population? or low income countr* or low income nation? or low income population? or lower income countr* or lower income nation? or lower income population? or underserved countr* or underserved nation? or underserved population? or underserved world or under served countr* or under served nation? or under served population? or under served world or deprived countr* or deprived nation? or deprived population? or deprived world or poor countr* or poor nation? or poor population? or poor world or poorer countr* or poorer nation? or poorer population? or poorer world or developing econom* or less developed econom* or lesser developed econom* or under developed econom* or underdeveloped econom* or middle income econom* or low income econom* or lower income econom* or low gdp or low gnp or low gross domestic or low gross national or lower gdp or lower gnp or lower gross domestic or lower gross national or lmic or lmics or third world or lami countr* or transitional countr* or emerging economies or emerging nation?).ti,ab,sh,kw. (2517925)

44 ("high burden country" or "high burden countries" or "high-burden country" or "high-burden countries" or "countdown country" or "countdown countries").ti,ab,kw,kf. (808)

45 43 or 44 [LMICs] (2518149)

46 42 and 45 [PERs, PREGNANCY - DATA COLLECTION, REGISTRIES - LMICs] (18846)

47 global health/ (16219)

48 ((global or international* or world*) and health*).ti,kw,kf. (45947)

49 exp adverse drug reaction/ (586248)

50 ((drug or drugs or medicine or medicines or medication* or pharmaceutical* or pharma-ceutical*) and expos*).ti,kw,kf. (9329)

51 (ae or co).fs. (2915988)

52 (safe or safety or side effect or side effects or undesirable effect or undesirable effects or treatment emergent or tolerability or toxicity or adrs).ti,kw,kf. (514988)

53 (adverse effect or adverse effects or adverse reaction or adverse reactions or adverse event or adverse events or adverse outcome or adverse outcomes).ti,kw,kf. (70731)

54 (AEFI and adverse).ti,kw,kf. (157)

55 "Global Alignment of Immuni#ation Safety Assessment in Pregnancy".tw,kw,kf. (20)

56 (GAIA and (alignment or immuni#ation or safety or pregnan*)).tw,kw,kf. (71)

57 exp vaccination/ (205483)

58 vaccine/ (67719)

59 exp bacterial vaccine/ or exp cell-based vaccine/ or conjugate vaccine/ or edible vaccine/ or exp fungus vaccine/ or exp inactivated vaccine/ or live vaccine/ or exp meningitis vaccine/ or exp nucleic acid vaccine/ or exp parasite vaccine/ or exp peptide vaccine/ or protein vaccine/ or exp subunit vaccine/ or exp toxoid vaccine/ or exp vector vaccine/ or virosome vaccine/ or exp virus vaccine/ (304544)

60 vaccin*.ti,kw,kf. (251220)

61 immunization/ (104541)

62 passive immunization/ (12623)

63 (immunit* and transfer*).ti,kw,kf. (1262)

64 (immunit* and (maternally-acqui* or passive*)).ti,kw,kf. (766)

65 ((antibod* or anti-bod*) and transfer*).ti,kw,kf. (2375)

66 ((maternal or pregnan*) and immuni#ation*).ti,kw,kf. (1766)

67 exp malaria/pc [Prevention] (14654)

68 exp antimalarial agent/ (161880)

69 (antimalarial* or anti-malarial*).ti,kw,kf. (11586)

70 exp cytomegalovirus infection/pc [Prevention] (5146)

71 respiratory syncytial virus infection/pc [Prevention] (1057)

72 exp Streptococcus infection/pc [Prevention] (10568)

73 exp Zika fever/pc [Prevention] (780)

74 exp antiretrovirus agent/ (216196)

75 (antiretroviral* or anti-retroviral* or "anti-HIV" or "anti-AIDS" or "AIDS drug" or "AIDS drugs").ti,kw,kf. (46286)

76 exp fetus development/ (29871)

77 ((fetal or foetal or fetus* or foetus*) and develop*).ti,kw,kf. (14926)

78 exp prenatal exposure/ (37294)

79 ((fetal or foetal or fetus* or foetus* or prenatal* or pre-natal*) and expos*).ti,kw,kf. (16619)

80 pregnancy outcome/ (72177)

81 ((birth or births or matern* or neonat* or neo-nat* or perinatal* or peri-natal*or peripartum or "peri-partum" or postnatal* or post-natal* or postpartum or "post-partum" or post-birth* or pregnanc*) and outcome*).ti,kw,kf. (55800)

82 ((birth or births) and defect*).ti,kw,kf. (4849)

83 child health/ (32206)

84 maternal welfare/ (15842)

85 (MNCH and (maternal* or newborn* or child*)).ti,kw,kf. (49)

86 or/47-85 [DRUGS, VACCINES, SAFETY, OUTCOMES] (4592324)

87 46 and 86 [PERs, PREGNANCY - DATA COLLECTION, REGISTRIES - LMICs - DRUGS, VACCINES, SAFETY, OUTCOMES] (5663)

88 exp animal/ or exp animal experimentation/ or exp animal model/ or exp animal experiment/ or nonhuman/ or exp vertebrate/ (30677570)

89 exp human/ or exp human experimentation/ or exp human experiment/ (23737031)

90 88 not 89 (6941713)

91 87 not 90 [ANIMAL-ONLY REMOVED] (5643)

92 editorial.pt. (729310)

93 91 not 92 [EDITORIALS REMOVED] (5614)

94 limit 93 to yr="2000-current" (5299)

***************************

CINAHL

| # | Query | Results |
| --- | --- | --- |
| S92 | S90 and S91 | 878 |
| S91 | DT 2000 - 2022 | 7,386,209 |
| S90 | S88 NOT S89 | 885 |
| S89 | PT editorial or opinion or commentary | 684,750 |
| S88 | S45 AND S87 | 891 |
| S87 | S46 OR S47 OR S48 OR S49 OR S50 OR S51 OR S52 OR S53 OR S54 OR S55 OR S56 OR S57 OR S58 OR S59 OR S60 OR S61 OR S62 OR S63 OR S64 OR S65 OR S66 OR S67 OR S68 OR S69 OR S70 OR S71 OR S72 OR S73 OR S74 OR S75 OR S76 OR S77 OR S78 OR S79 OR S80 OR S81 OR S82 OR S83 OR S84 OR S85 OR S86 | 354,157 |
| S86 | TI (MNCH AND (maternal* OR newborn* OR child*)) | 17 |
| S85 | (MH "Maternal-Child Health") | 3,727 |
| S84 | (MH "Child Health") | 17,073 |
| S83 | TI (birth OR births) and defect* | 969 |
| S82 | TI (birth OR births OR matern* OR neonat* OR neo-nat* OR perinatal* OR peri-natal* OR peripartum OR "peri-partum" OR postnatal* OR post-natal* OR postpartum OR "post-partum" OR post-birth* OR pregnanc*) AND outcome* | 17,343 |
| S81 | (MH "Pregnancy Outcomes") | 27,127 |
| S80 | TI (fetal OR foetal OR fetus* OR foetus* or prenatal* or pre-natal*) AND expos* | 2,603 |
| S79 | (MH "Prenatal Exposure Delayed Effects") | 6,314 |
| S78 | TI (fetal OR foetal OR fetus* OR foetus*) AND develop* | 1,028 |
| S77 | (MH "Fetal Development") | 7,851 |
| S76 | TI antiretroviral* OR anti-retroviral* OR "anti-HIV" OR "anti-AIDS" OR "AIDS drug" OR "AIDS drugs" | 9,126 |
| S75 | (MH "Anti-Retroviral Agents+") | 24,911 |
| S74 | (MH "Zika Virus Infections/PC") | 168 |
| S73 | (MH "Streptococcal Infections+/PC") | 2,280 |
| S72 | (MH "Respiratory Syncytial Virus Infections/PC") | 583 |
| S71 | (MH "Hepatitis E/PC") | 69 |
| S70 | (MH "Cytomegalovirus Infections+/PC") | 509 |
| S69 | TI antimalarial* or (anti W0 malarial*) | 714 |
| S68 | (MH "Antimalarials+") | 8,320 |
| S67 | (MH "Malaria/PC") | 3,293 |
| S66 | TI (maternal* or pregnan*) and immuni?ation* | 253 |
| S65 | TI ((antibod* or (anti W0 bod*)) and transfer*) | 79 |
| S64 | TI immunit* AND (passive* or "maternally-acquired") | 13 |
| S63 | TI immunit* AND transfer* | 16 |
| S62 | (MH "Immunity, Maternally Acquired") | 232 |
| S61 | (MH "Immunization Programs") | 6,426 |
| S60 | (MH "Viral Vaccines+") | 36,206 |
| S59 | (MH "Vaccines, Combined+") | 3,562 |
| S58 | (MH "Toxoids+") | 2,372 |
| S57 | (MH "Bacterial Vaccines+") | 10,296 |
| S56 | (MH "Vaccines") | 9,533 |
| S55 | (MH "Immunization+") | 31,914 |
| S54 | TI ( GAIA AND (alignment or immuni?ation or safety or pregnan*) ) OR AB ( GAIA AND (alignment or immuni?ation or safety or pregnan*) ) | 6 |
| S53 | TI ( "Global Alignment of Immunisation Safety Assessment in Pregnancy" or "Global Alignment of Immunization Safety Assessment in Pregnancy" ) OR AB ( "Global Alignment of Immunisation Safety Assessment in Pregnancy" or "Global Alignment of Immunization Safety Assessment in Pregnancy" ) | 0 |
| S52 | TI AEFI and adverse | 5 |
| S51 | TI "adverse effect" or "adverse effects" or "adverse reaction" or "adverse reactions" or "adverse event" or "adverse events" or "adverse outcome" or "adverse outcomes" | 12,615 |
| S50 | TI safe or safety or "side effect" or "side effects" or "undesirable effect" or "undesirable effects" or "treatment emergent" or tolerability or toxicity or adrs | 113,937 |
| S49 | TI (drug or drugs or medicine or medicines or medication* or pharmaceutical* or pharma-ceutical*) AND expos* | 1,420 |
| S48 | (MH "Adverse Drug Event") | 16,207 |
| S47 | TI (global or international* or world*) and health* | 22,194 |
| S46 | (MH "World Health") | 29,391 |
| S45 | S39 AND S44 | 4,402 |
| S44 | S40 OR S41 OR S42 OR S43 | 379,212 |
| S43 | TI ( "high burden country" or "high burden countries" or "high-burden country" or "high-burden countries" or "countdown country" or "countdown countries" ) OR AB ( "high burden country" or "high burden countries" or "high-burden country" or "high-burden countries" or "countdown country" or "countdown countries" ) | 188 |
| S42 | TI ( "developing country" or "developing countries" or "developing nation" or "developing nations" or "developing population" or "developing populations" or "developing world" or "less developed country" or "less developed countries" or "less developed nation" or "less developed nations" or "less developed population" or "less developed populations" or "less developed world" or "lesser developed country" or "lesser developed countries" or "lesser developed nation" or "lesser developed nations" or "lesser developed population" or "lesser developed populations" or "lesser developed world" or "under developed country" or "under developed countries" or "under developed nation" or "under developed nations" or "under developed population" or "under developed populations" or "under developed world" or "underdeveloped country" or "underdeveloped countries" or "underdeveloped nation" or "underdeveloped nations" or "underdeveloped population" or "underdeveloped populations" or "underdeveloped world" or "middle income country" or "middle income countries" or "middle income nation" or "middle income nations" or "middle income population" or "middle income populations" or "low income country" or "low income countries" or "low income nation" or "low income nations" or "low income population" or "low income populations" or "lower income country" or "lower income countries" or "lower income nation" or "lower income nations" or "lower income population" or "lower income populations" or "underserved country" or "underserved countries" or "underserved nation" or "underserved nations" or "underserved population" or "underserved populations" or "underserved world" or "under served country" or "under served countries" or "under served nation" or "under served nations" or "under served population" or "under served populations" or "under served world" or "deprived country" or "deprived countries" or "deprived nation" or "deprived nations" or "deprived population" or "deprived populations" or "deprived world" or "poor country" or "poor countries" or "poor nation" or "poor nations" or "poor population" or "poor populations" or "poor world" or "poorer country" or "poorer countries" or "poorer nation" or "poorer nations" or "poorer population" or "poorer populations" or "poorer world" or "developing economy" or "developing economies" or "less developed economy" or "less developed economics" or "lesser developed economy" or "lesser developed economies" or "under developed economy" or "under developed economies" or "underdeveloped economy" or "underdeveloped economies" or "middle income economy" or "middle income economies" or "low income economy" or "low income economies" or "lower income economy" or "lower income economies" or "low gdp" or "low gnp" or "low gross domestic" or "low gross national" or "lower gdp" or "lower gnp" or "lower gross domestic" or "lower gross national" or lmic or lmics or "third world" or "lami country" or "lami countries" or "transitional country" or "transitional economies" or "emerging economy" or "emerging economies" or "emerging nation" or "emerging nations" ) OR AB ( "developing country" or "developing countries" or "developing nation" or "developing nations" or "developing population" or "developing populations" or "developing world" or "less developed country" or "less developed countries" or "less developed nation" or "less developed nations" or "less developed population" or "less developed populations" or "less developed world" or "lesser developed country" or "lesser developed countries" or "lesser developed nation" or "lesser developed nations" or "lesser developed population" or "lesser developed populations" or "lesser developed world" or "under developed country" or "under developed countries" or "under developed nation" or "under developed nations" or "under developed population" or "under developed populations" or "under developed world" or "underdeveloped country" or "underdeveloped countries" or "underdeveloped nation" or "underdeveloped nations" or "underdeveloped population" or "underdeveloped populations" or "underdeveloped world" or "middle income country" or "middle income countries" or "middle income nation" or "middle income nations" or "middle income population" or "middle income populations" or "low income country" or "low income countries" or "low income nation" or "low income nations" or "low income population" or "low income populations" or "lower income country" or "lower income countries" or "lower income nation" or "lower income nations" or "lower income population" or "lower income populations" or "underserved country" or "underserved countries" or "underserved nation" or "underserved nations" or "underserved population" or "underserved populations" or "underserved world" or "under served country" or "under served countries" or "under served nation" or "under served nations" or "under served population" or "under served populations" or "under served world" or "deprived country" or "deprived countries" or "deprived nation" or "deprived nations" or "deprived population" or "deprived populations" or "deprived world" or "poor country" or "poor countries" or "poor nation" or "poor nations" or "poor population" or "poor populations" or "poor world" or "poorer country" or "poorer countries" or "poorer nation" or "poorer nations" or "poorer population" or "poorer populations" or "poorer world" or "developing economy" or "developing economies" or "less developed economy" or "less developed economics" or "lesser developed economy" or "lesser developed economies" or "under developed economy" or "under developed economies" or "underdeveloped economy" or "underdeveloped economies" or "middle income economy" or "middle income economies" or "low income economy" or "low income economies" or "lower income economy" or "lower income economies" or "low gdp" or "low gnp" or "low gross domestic" or "low gross national" or "lower gdp" or "lower gnp" or "lower gross domestic" or "lower gross national" or lmic or lmics or "third world" or "lami country" or "lami countries" or "transitional country" or "transitional economies" or "emerging economy" or "emerging economies" or "emerging nation" or "emerging nations" ) | 39,219 |
| S41 | TI ( mali or malta or micronesia or "federated states of micronesia" or kiribati or "marshall islands" or nauru or "northern mariana islands" or palau or tuvalu or mauritania or mauritius or mexico or moldova or moldovian or mongolia or montenegro or morocco or ifni or mozambique or "portuguese east africa" or myanmar or burma or namibia or nepal or "netherlands antilles" or nicaragua or niger or nigeria or oman or muscat or pakistan or panama or "papua new guinea" or "new guinea" or paraguay or peru or philippines or philipines or phillipines or phillippines or poland or "polish people's republic" or portugal or "portuguese republic" or "puerto rico" or romania or russia or "russian federation" or ussr or "soviet union" or "union of soviet socialist republics" or rwanda or ruanda or samoa or "pacific islands" or polynesia or "samoan islands" or "navigator island" or "navigator islands" or "sao tome and principe" or "saudi arabia" or senegal or serbia or seychelles or "sierra leone" or slovakia or "slovak republic" or slovenia or melanesia or "solomon island" or "solomon islands" or "norfolk island" or "norfolk islands" or somalia or "south africa" or "south sudan" or "sri lanka" or ceylon or "saint kitts and nevis" or "st. kitts and nevis" or "saint lucia" or "st. lucia" or "saint Vincent and the grenadines" or "saint vincent" or "st. vincent" or grenadines or sudan or suriname or surinam or "dutch guiana" or "netherlands guiana" or syria or "syrian arab republic" or tajikistan or tadjikistan or tadzhikistan or tadzhik or tanzania or tanganyika or thailand or siam or "timor leste" or "east timor" or togo or "togolese republic" or tonga or "Trinidad and tobago" or trinidad or tobago or tunisia or turkey or turkmenistan or turkmen or uganda or ukraine or uruguay or uzbekistan or uzbek or vanuatu or "new hebrides" or venezuela or vietnam or "viet nam" or "middle east" or "west bank" or gaza or palestine or yemen or yugoslavia or zambia or zimbabwe or "northern rhodesia" or "global south" or "africa south of the sahara" or "sub-saharan africa" or "subsaharan africa" or "africa, central" or "central africa" or "africa, northern" or "north africa" or "northern africa" or magreb or maghrib or sahara or "africa, southern" or "southern africa" or "africa, eastern" or "east africa" or "eastern africa" or "africa, western" or "west africa" or "western africa" or "west indies" or caribbean or "central america" or "latin america" or "south and central america" or "south america" or "asia, central" or "central asia" or "asia, northern" or "north asia" or "northern asia" or "asia, southeastern" or "southeastern asia" or "south eastern asia" or "southeast asia" or "south east asia" or "asia, western" or "western asia" or "europe, eastern" or "east europe" or "eastern europe" ) OR AB ( mali or malta or micronesia or "federated states of micronesia" or kiribati or "marshall islands" or nauru or "northern mariana islands" or palau or tuvalu or mauritania or mauritius or mexico or moldova or moldovian or mongolia or montenegro or morocco or ifni or mozambique or "portuguese east africa" or myanmar or burma or namibia or nepal or "netherlands antilles" or nicaragua or niger or nigeria or oman or muscat or pakistan or panama or "papua new guinea" or "new guinea" or paraguay or peru or philippines or philipines or phillipines or phillippines or poland or "polish people's republic" or portugal or "portuguese republic" or "puerto rico" or romania or russia or "russian federation" or ussr or "soviet union" or "union of soviet socialist republics" or rwanda or ruanda or samoa or "pacific islands" or polynesia or "samoan islands" or "navigator island" or "navigator islands" or "sao tome and principe" or "saudi arabia" or senegal or serbia or seychelles or "sierra leone" or slovakia or "slovak republic" or slovenia or melanesia or "solomon island" or "solomon islands" or "norfolk island" or "norfolk islands" or somalia or "south africa" or "south sudan" or "sri lanka" or ceylon or "saint kitts and nevis" or "st. kitts and nevis" or "saint lucia" or "st. lucia" or "saint Vincent and the grenadines" or "saint vincent" or "st. vincent" or grenadines or sudan or suriname or surinam or "dutch guiana" or "netherlands guiana" or syria or "syrian arab republic" or tajikistan or tadjikistan or tadzhikistan or tadzhik or tanzania or tanganyika or thailand or siam or "timor leste" or "east timor" or togo or "togolese republic" or tonga or "Trinidad and tobago" or trinidad or tobago or tunisia or turkey or turkmenistan or turkmen or uganda or ukraine or uruguay or uzbekistan or uzbek or vanuatu or "new hebrides" or venezuela or vietnam or "viet nam" or "middle east" or "west bank" or gaza or palestine or yemen or yugoslavia or zambia or zimbabwe or "northern rhodesia" or "global south" or "africa south of the sahara" or "sub-saharan africa" or "subsaharan africa" or "africa, central" or "central africa" or "africa, northern" or "north africa" or "northern africa" or magreb or maghrib or sahara or "africa, southern" or "southern africa" or "africa, eastern" or "east africa" or "eastern africa" or "africa, western" or "west africa" or "western africa" or "west indies" or caribbean or "central america" or "latin america" or "south and central america" or "south america" or "asia, central" or "central asia" or "asia, northern" or "north asia" or "northern asia" or "asia, southeastern" or "southeastern asia" or "south eastern asia" or "southeast asia" or "south east asia" or "asia, western" or "western asia" or "europe, eastern" or "east europe" or "eastern europe" ) | 148,518 |
| S40 | TI ( afghanistan or albania or algeria or "american samoa" or angola or antigua or barbuda or argentina or armenia or armenian or aruba or azerbaijan or bahrain or bangladesh or barbados or belarus or byelarus or belorussia or byelorussian or belize or "british honduras" or benin or dahomey or bhutan or bolivia or "bosnia herzegovina" or bosnia or herzegovina or botswana or bechuanaland or brazil or brasil or bulgaria or "burkina faso" or "burkina fasso" or "upper volta" or burundi or urundi or "cabo verde" or "cape verde" or cambodia or kampuchea or "khmer republic" or cameroon or cameron or cameroun or "central african republic" or "ubangi shari" or chad or chile or china or colombia or comoros or "comoro islands" or "iles comores" or mayotte or "democratic republic of the congo" or "democratic republic congo" or congo or zaire or "costa rica" or "cote d’ivoire" or "cote d’ ivoire" or "cote divoire" or "cote d ivoire" or "ivory coast" or croatia or cuba or cyprus or "czech republic" or czechoslovakia or djibouti or "french somaliland" or dominica or "dominican republic" or ecuador or egypt or "united arab republic" or "el salvador" or "equatorial guinea" or "spanish guinea" or eritrea or estonia or eswatini or swaziland or ethiopia or fiji or gabon or "gabonese republic" or gambia or "georgia (republic)" or georgian or ghana or "gold coast" or gibraltar or greece or grenada or guam or guatemala or guinea or "guinea bissau" or guyana or "british guiana" or haiti or hispaniola or honduras or hungary or india or indonesia or timor or iran or iraq or "isle of man" or jamaica or jordan or kazakhstan or kazakh or kenya or "democratic people’s republic of korea" or "republic of korea" or "north korea" or "south korea" or korea or kosovo or kyrgyzstan or kirghizia or kirgizstan or "kyrgyz republic" or kirghiz or laos or "lao pdr" or "lao people's democratic republic" or latvia or lebanon or lebanese republic or lesotho or basutoland or liberia or libya or "libyan arab jamahiriya" or lithuania or macau or macao or republic of "north macedonia" or macedonia or madagascar or "malagasy republic" or malawi or nyasaland or malaysia or "malay federation" or "malaya federation" or maldives or "indian ocean islands" or "indian ocean" ) OR AB ( afghanistan or albania or algeria or "american samoa" or angola or antigua or barbuda or argentina or armenia or armenian or aruba or azerbaijan or bahrain or bangladesh or barbados or belarus or byelarus or belorussia or byelorussian or belize or "british honduras" or benin or dahomey or bhutan or bolivia or "bosnia herzegovina" or bosnia or herzegovina or botswana or bechuanaland or brazil or brasil or bulgaria or "burkina faso" or "burkina fasso" or "upper volta" or burundi or urundi or "cabo verde" or "cape verde" or cambodia or kampuchea or "khmer republic" or cameroon or cameron or cameroun or "central african republic" or "ubangi shari" or chad or chile or china or colombia or comoros or "comoro islands" or "iles comores" or mayotte or "democratic republic of the congo" or "democratic republic congo" or congo or zaire or "costa rica" or "cote d’ivoire" or "cote d’ ivoire" or "cote divoire" or "cote d ivoire" or "ivory coast" or croatia or cuba or cyprus or "czech republic" or czechoslovakia or djibouti or "french somaliland" or dominica or "dominican republic" or ecuador or egypt or "united arab republic" or "el salvador" or "equatorial guinea" or "spanish guinea" or eritrea or estonia or eswatini or swaziland or ethiopia or fiji or gabon or "gabonese republic" or gambia or "georgia (republic)" or georgian or ghana or "gold coast" or gibraltar or greece or grenada or guam or guatemala or guinea or "guinea bissau" or guyana or "british guiana" or haiti or hispaniola or honduras or hungary or india or indonesia or timor or iran or iraq or "isle of man" or jamaica or jordan or kazakhstan or kazakh or kenya or "democratic people’s republic of korea" or "republic of korea" or "north korea" or "south korea" or korea or kosovo or kyrgyzstan or kirghizia or kirgizstan or "kyrgyz republic" or kirghiz or laos or "lao pdr" or "lao people's democratic republic" or latvia or lebanon or lebanese republic or lesotho or basutoland or liberia or libya or "libyan arab jamahiriya" or lithuania or macau or macao or republic of "north macedonia" or macedonia or madagascar or "malagasy republic" or malawi or nyasaland or malaysia or "malay federation" or "malaya federation" or maldives or "indian ocean islands" or "indian ocean" ) | 230,968 |
| S39 | S1 OR S38 | 15,244 |
| S38 | S16 AND S37 | 15,243 |
| S37 | S17 OR S18 OR S19 OR S20 OR S21 OR S22 OR S23 OR S24 OR S25 OR S26 OR S27 OR S28 OR S29 OR S30 OR S31 OR S32 OR S33 OR S34 OR S35 OR S36 | 354,620 |
| S36 | (MH "Product Surveillance+/SN") | 1 |
| S35 | (MH "Maternal Health Services+/SN") | 1,245 |
| S34 | TI pharmacovigilan* or (pharmaco W0 vigilan*) | 846 |
| S33 | (MH "Product Surveillance+") | 2,417 |
| S32 | TI "data system" or "data systems" or "information system" or "information systems" | 4,466 |
| S31 | (MH "Health Information Systems") | 3,566 |
| S30 | TI (decision* AND support* AND clinical*) | 1,865 |
| S29 | (MH "Decision Support Systems, Clinical") | 6,139 |
| S28 | TI surveillance* | 15,975 |
| S27 | (MH "Population Surveillance+") | 11,277 |
| S26 | TI survey* | 71,609 |
| S25 | (MH "Surveys") | 157,567 |
| S24 | TI registry or registries or eregistr* or "e-registry" or "e-registries" | 13,946 |
| S23 | TI (preliminary or "pilot project" or "pilot projects") AND data | 682 |
| S22 | TI "focus group" or "focus groups" | 2,957 |
| S21 | (MH "Focus Groups") | 48,414 |
| S20 | TI databas* or "data base" or "data bases" or databank* or "data bank" or "data banks" or dataset* or "data set" or "data sets" | 16,019 |
| S19 | TI data AND (accumulat* or accura* or assembl* or captur* or collect* or compil* or coordinat* or co-ordinat* or gather* or hub or hubs) | 4,425 |
| S18 | (MH "Data Curation") | 184 |
| S17 | (MH "Data Collection") | 49,942 |
| S16 | S2 OR S3 OR S4 OR S5 OR S6 OR S7 OR S8 OR S9 OR S10 OR S11 OR S12 OR S13 OR S14 OR S15 | 290,175 |
| S15 | TI maternal AND (fetal or fetus or foetal or foetus) | 4,004 |
| S14 | TI (maternal* or maternity) AND (care or health* or service or services) | 9,718 |
| S13 | (MH "Maternal Health Services") | 11,199 |
| S12 | TI (maternal* or maternity) AND (contact* or expos*) | 1,028 |
| S11 | (MH "Maternal Exposure") | 2,588 |
| S10 | TI perinatal* or "peri-natal" or "peri-natally" or peripartum or "peri-partum" | 12,858 |
| S9 | (MH "Perinatal Care") | 5,019 |
| S8 | TI prenatal* or antenatal* or "ante-natal" or "ante-natally" or antepartum or "ante-partum" | 20,877 |
| S7 | (MH "Prenatal Care") | 19,422 |
| S6 | TI pregnan* | 80,123 |
| S5 | (MH "Pregnancy Trimesters+") | 13,521 |
| S4 | (MH "Expectant Mothers") | 11,382 |
| S3 | (MH "Pregnancy Complications+") | 106,825 |
| S2 | (MH "Pregnancy") OR (MH "Pregnancy, Multiple+") OR (MH "Pregnancy, Unplanned") OR (MH "Pregnancy, Unwanted") OR (MH "Pregnancy, High Risk") OR (MH "Pregnancy, Prolonged") | 227,240 |
| S1 | TI ( "pregnancy exposure database" OR "pregnancy exposure databases" OR "pregnancy exposure data base" OR "pregnancy exposure data bases" OR "pregnancy exposure registry" OR "pregnancy exposure registries" ) OR AB ( "pregnancy exposure database" OR "pregnancy exposure databases" OR "pregnancy exposure data base" OR "pregnancy exposure data bases" OR "pregnancy exposure registry" OR "pregnancy exposure registries" ) | 7 |

Global Index Medicus

tw:((tw:(pregnan* exposure* database*)) OR (tw:(pregnan* exposure* "data base")) OR (tw:(pregnan* exposure* "data bases")) OR (tw:(pregnan* exposure* databank*)) OR (tw:(pregnan* exposure* "data bank")) OR (tw:(pregnan* exposure* "data banks")) OR (tw:(pregnan* exposure* register)) OR (tw:(pregnan* exposure* registries)) OR (tw:(pregnan* exposure* registry)) OR (tw:(pregnan* exposure* registries))) – *108 records*

tw:((ti:((pregnan* OR prenatal* OR antenatal* OR "ante-natal" OR "ante-natally" OR antepartum OR "ante-partum" OR perinatal* OR "peri-natal" OR "peri-natally" OR peripartum OR "peri-partum" OR maternal*) AND (data OR database* OR databank* OR register OR registers OR registry OR registries OR survey* OR surveillance OR pharmacovigilan* OR "pharmaco-vigilance") AND (safe OR safety OR "side effect" of "side effects" OR "undesirable effect" OR "undesirable effects" OR "treatment emergent" OR tolerability OR toxicity OR adrs OR aefi)))) – *361 records*

tw:(ti:((pregnan* OR prenatal* OR antenatal* OR "ante-natal" OR "ante-natally" OR antepartum OR "ante-partum" OR perinatal* OR "peri-natal" OR "peri-natally" OR peripartum OR "peri-partum" OR maternal*) AND (data OR database* OR databank* OR register OR registers OR registry OR registries OR survey* OR surveillance OR pharmacovigilan* OR "pharmaco-vigilance") AND (MNCH or "maternal health" or "child health" or "infant health" or "birth defect" or "birth defects" or "birth outcome" or "birth outcomes" or "pregnancy outcome" or "pregnancy outcomes" or "neonatal outcome" or "neonatal outcomes"))) – *50 records*

tw:(ti:((pregnan* OR prenatal* OR antenatal* OR "ante-natal" OR "ante-natally" OR antepartum OR "ante-partum" OR perinatal* OR "peri-natal" OR "peri-natally" OR peripartum OR "peri-partum" OR maternal*) AND (data OR database* OR databank* OR register OR registers OR registry OR registries OR survey* OR surveillance OR pharmacovigilan* OR "pharmaco-vigilance") AND (vaccine or vaccines or vaccination* or immunization* or immunization* or “maternally-acquired”))) – *0 records*

tw:((ti:((pregnan* OR prenatal* OR antenatal* OR "ante-natal" OR "ante-natally" OR antepartum OR "ante-partum" OR perinatal* OR "peri-natal" OR "peri-natally" OR peripartum OR "peri-partum" OR maternal*) AND (data OR database* OR databank* OR register OR registers OR registry OR registries OR survey* OR surveillance OR pharmacovigilan* OR "pharmaco-vigilance") AND (drug OR drugs OR medicine OR medicines OR medication* OR pharmaceutical* OR "pharma-ceutical" OR "pharma-ceuticals" OR vaccin* OR immun* OR antimalarial* OR "anti-malarial" OR "anti-malarials" OR antiviral* OR "anti-viral" OR "anti-virals" OR antiretroviral* OR "anti-retroviral" OR "anti-retrovirals" OR "Anti-HIV" OR "Anti-AIDS")))) – *10 records*

tw:((ti:((pregnan* OR prenatal* OR antenatal* OR "ante-natal" OR "ante-natally" OR antepartum OR "ante-partum" OR perinatal* OR "peri-natal" OR "peri-natally" OR peripartum OR "peri-partum" OR maternal*) AND (data OR database* OR databank* OR register OR registers OR registry OR registries OR survey* OR surveillance OR pharmacovigilan* OR "pharmaco-vigilance") AND (fetal* OR foetal* OR fetus* OR foetus* OR neonat* OR newborn* OR infant OR infants OR infanc* OR child*) ))) – *125 records*

tw:((ti:((pregnan* OR prenatal* OR antenatal* OR "ante-natal" OR "ante-natally" OR antepartum OR "ante-partum" OR perinatal* OR "peri-natal" OR "peri-natally" OR peripartum OR "peri-partum" OR maternal*) AND (data OR database* OR databank* OR register OR registers OR registry OR registries OR survey* OR surveillance OR pharmacovigilan* OR "pharmaco-vigilance"))) AND (tw:(drug OR drugs OR medicine OR medicines OR medication* OR pharmaceutical* OR "pharma-ceutical" OR "pharma-ceuticals" OR vaccin* OR immunisation* OR immunization* OR antimalarial* OR "anti-malarial" OR "anti-malarials" OR antiviral* OR "anti-viral" OR "anti-virals" OR antiretroviral* OR "anti-retroviral" OR "anti-retrovirals" OR "Anti-HIV" OR "Anti-AIDS"))) – *71 records*

**TOTAL: 725 records**

Google Scholar

"pregnant|pregnancy|prenatal"+"registry|registries|surveillance|pharmacovigilance" + "expose|exposed|exposes|exposure|exposures"+[names of LMICs]

Data extraction form

**PART 1: STUDY IDENTIFICATION / INCLUSION STATUS**

Resource title:

Author(s):

Year of publication:

Resource type:

1. 1 - Peer reviewed journal

2. 2 - Grey literature

3. 3 - Registry

4. 4 - Website

5. 5 - Key informant survey / interview

6. 6 - Other

Database resource pulled from:

Final status:

1. 1 - included

2. 2 - excluded

3. 3 - incomplete/unclear

If excluded, explain reasoning:

**PART 2: REGISTRY INFORMATION OR OTHER RESOURCE**

Name of registry or other resource:

Primary goals/aims:

Funding source:

Years of operation:

Current status

1. Active

2. Inactive

3. On hold

Country(ies) where located:

Country representativeness:

1. Multi-national

2. National

3. State/Provincial

4. District

5. Sub-district

6. Community

7. Health clinic

8. Hospital

9. Other

Drug/vaccine exposure name and type:

Duration of follow up:

Current sample size:

1. <500 participants

2. 500 - 1,999 participants

3. 2,000 - 5,000 participants

4. 5,001 - 10,000 participants

5. 10,001 - 20,000 participants

6. >20,000 participants

Data collection:

1. Administrative databases

2. Medical databases

3. Registries

4. Research study

5. Program implementation

6. Other

Methodology:

Terminology and data system used:

**PART 3: CHARACTERISTICS OF INCLUDED POPULATION**

Age ranges included:

Target population:

1. Pregnant women

2. Non-pregnant women / women of reproductive age

3. Children

4. Infants (<28 days old)

5. General population

6. Other

Gestational age

1. First trimester

2. Second trimester

3. Third trimester

Underlying medical condition(s):

Maternal outcome(s) recorded:

1. Thrombosis and/or thrombocytopenia syndrome

2. Antenatal hospitalization (not including delivery)

3. Disability

4. Gestational diabetes

5. Gestational hypertension

6. Premature rupture of membranes

7. Preterm labor

8. Preeclampsia / Eclampsia

9. Post-partum hemorrhage

10. Antenatal hemorrhage

11. Corporeal infection

12. Spontaneous abortion / miscarriage / pregnancy loss (example: prior to 20 weeks gestation)

13. Maternal death (example: within 42 days of termination of pregnancy)

14. Late maternal death (example: >42 days of termination of pregnancy)

15. Other

Maternal death cause:

1. Direct cause

2. Indirect cause

Neonatal outcome(s) recorded:

1. Preterm birth

2. Small size for gestational age / restricted fetal growth

3. Still birth (death after 28 weeks of pregnancy but before birth)

4. Live birth

5. Congenital anomaly / birth defect

6. Death

7. Other

Neonatal death timeframe

1. Early neonatal (0-7 days)

2. Late neonatal (8-28 days)

3. Post neonatal (29 days - 1 year)

Which congenital anomaly does this monitor (if any)?

Infant/child outcome(s) recorded:

1. Infections

2. Respiratory illness

3. Developmental outcomes

4. Other

What is the duration of follow up?

**PART 4: KEY FINDINGS**

Populate as best you can if information is provided; answer N/A if it is not relevant or N/P if it is not provided.

Strengths of the registry or other resource:

Weaknesses / gaps of registry or other resource:

Challenges of registry or other resource in its specific context:

Does this resource have the possibility to add new interventions?

1. Yes

2. No

3. Maybe

4. Unknown Why?

Can this resource be combined with other systems?

1. Yes

2. No

3. Maybe

4. Unknown Why?

Any upcoming changes to the resource:

Additional comments

**PART 1: STUDY IDENTIFICATION / INCLUSION STATUS**

Resource title:

Author(s):

Year of publication:

Resource type:

1. 1 - Peer reviewed journal

2. 2 - Grey literature

3. 3 - Registry

4. 4 - Website

5. 5 - Key informant survey / interview

6. 6 - Other

Database resource pulled from:

Final status:

1. 1 - included

2. 2 - excluded

3. 3 - incomplete/unclear

If excluded, explain reasoning:

**PART 2: REGISTRY INFORMATION OR OTHER RESOURCE**

Name of registry or other resource:

Primary goals/aims:

Funding source:

Years of operation:

Current status

1. Active

2. Inactive

3. On hold

Country(ies) where located:

Country representativeness:

1. Multi-national

2. National

3. State/Provincial

4. District

5. Sub-district

6. Community

7. Health clinic

8. Hospital

9. Other

Drug/vaccine exposure name and type:

Duration of follow up:

Current sample size:

1. <500 participants

2. 500 - 1,999 participants

3. 2,000 - 5,000 participants

4. 5,001 - 10,000 participants

5. 10,001 - 20,000 participants

6. >20,000 participants

Data collection:

1. Administrative databases

2. Medical databases

3. Registries

4. Research study

5. Program implementation

6. Other

Methodology:

Terminology and data system used:

**PART 3: CHARACTERISTICS OF INCLUDED POPULATION**

Age ranges included:

Target population:

1. Pregnant women

2. Non-pregnant women / women of reproductive age

3. Children

4. Infants (<28 days old)

5. General population

6. Other

Gestational age

1. First trimester

2. Second trimester

3. Third trimester

Underlying medical condition(s):

Maternal outcome(s) recorded:

1. Thrombosis and/or thrombocytopenia syndrome

2. Antenatal hospitalization (not including delivery)

3. Disability

4. Gestational diabetes

5. Gestational hypertension

6. Premature rupture of membranes

7. Preterm labor

8. Preeclampsia / Eclampsia

9. Post-partum hemorrhage

10. Antenatal hemorrhage

11. Corporeal infection

12. Spontaneous abortion / miscarriage / pregnancy loss (example: prior to 20 weeks gestation)

13. Maternal death (example: within 42 days of termination of pregnancy)

14. Late maternal death (example: >42 days of termination of pregnancy)

15. Other

Maternal death cause:

1. Direct cause

2. Indirect cause

Neonatal outcome(s) recorded:

1. Preterm birth

2. Small size for gestational age / restricted fetal growth

3. Still birth (death after 28 weeks of pregnancy but before birth)

4. Live birth

5. Congenital anomaly / birth defect

6. Death

7. Other

Neonatal death timeframe

1. Early neonatal (0-7 days)

2. Late neonatal (8-28 days)

3. Post neonatal (29 days - 1 year)

Which congenital anomaly does this monitor (if any)?

Infant/child outcome(s) recorded:

1. Infections

2. Respiratory illness

3. Developmental outcomes

4. Other

What is the duration of follow up?

**PART 4: KEY FINDINGS**

Populate as best you can if information is provided; answer N/A if it is not relevant or N/P if it is not provided.

Strengths of the registry or other resource:

Weaknesses / gaps of registry or other resource:

Challenges of registry or other resource in its specific context:

Does this resource have the possibility to add new interventions?

1. Yes

2. No

3. Maybe

4. Unknown Why?

Can this resource be combined with other systems?

1. Yes

2. No

3. Maybe

4. Unknown Why?

Any upcoming changes to the resource:

Additional comments

Key informant survey and interview

Pregnancy Exposure Data and Resources Stakeholder Survey

We are conducting a landscape analysis in collaboration with WHO to identify current and recent resources, including pregnancy exposure and surveillance registries, databases, cohort surveys, and routinely collected data, that record exposure to medicines and vaccines during pregnancy and maternal and perinatal outcomes in low- and middle-income countries (LMICs). We are asking for your help in identifying examples of these resources. We may follow up with you to discuss the appropriateness or fit for purpose of the resource you identify. Our goal is to understand what is currently available in LMICs and make connections for future evaluation of maternal use of medicines and vaccines in the product pipeline.

You have been identified as someone who is knowledgeable about or involved with these resources in LMICs. Please complete the following form for each resource you know of. We will ask for your name and contact information so that we may follow up with you for further information, if necessary. All of the personal information you provide will be kept confidential. When we report our findings, if we need to mention something you have said or information you have provided, we will refer to you by a unique study ID to keep your identity confidential. By submitting the form, you are agreeing to participate and allow us to use the information you have provided.

Please fill out the following questions to the best of your knowledge. If there are any specific points that are not included as options in the dropdown menus that are relevant to the resource, please type in the answer and hit “enter”.

**Participant details**

Please note we may contact you to follow up about the resource you describe if we have any questions.

Name*: ____________________________________________________________________________

Email*: ____________________________________________________________________________

Organization*: ______________________________________________________________________

Job Title: ___________________________________________________________________________

**Resource details**

Below we will be asking you to fill in information about any resources you are familiar with as outlined above. If you know of multiple resources that should be brought to our attention, please fill out a separate survey for each resource. As a reminder, resources can include pregnancy exposure and surveillance registries, databases, cohort surveys, and routinely collected data, that record exposure to medicines and vaccines during pregnancy and maternal and perinatal outcomes in low- and middle-income countries (LMICs)

Resource or Project Name: ___________________________________________________________

Please provide a link to the resource if available: __________________________________________

What location(s) does the resource cover (country/countries or region(s))? ____________________

Who oversees or maintains the resource? Please provide the name of the organization/s or specific person(s) and their contact information if available.

Name of organization: _______________________________________________________________

Primary contact name: _______________________________________________________________

Primary contact email: _______________________________________________________________

How is data collected? Select all that apply.

- Administrative databases
- Medical databases
- Registries
- Research study
- Program implementation
- Other

If you selected “other”, please specify: ______________________________________________

Any additional details you would like to provide? :_______________________________

The data in this resource are captured at a:

- Multi-national
- National
- State / Provincial
- District
- Sub-district
- Community
- Health clinic
- Hospital
- Other

If you selected “other”, please specify: ______________________________________________

How are these data collected?

- Retrospectively
- Prospectively

How many individuals are enrolled in this resource (total)?

- <500 participants
- 500 – 2,000 participants
- 2,000 – 5,000 participants
- 5,001 – 10,000 participants
- 10,001 – 20,000 participants
- >20,000 participants

What population(s) are the target for this resource. Select all that apply.

- Pregnant women
- Non-pregnant women / women of reproductive age
- Breastfeeding women
- Children
- Infants (<28 days old)
- General population

What intervention(s) does this resource include? Select all that apply.

- Vaccine/Immunization
  - COVID-19
  - Influenza
  - Meningococcus
  - Tetanus toxoid
  - Pertussis
  - Other

If you selected “other”, please specify: ___________________________

- Medicines/Drugs/Biologics
  - Antimalarials
  - Antiretrovirals/HIV/AIDS
  - Medicines related to mental health
  - Medicines related to autoimmune diseases
  - Medicines related to cancer
  - Medicines related to diabetes
  - Medicines related to epilepsy
  - Other

If you selected “other”, please specify: ______________________________________________

What outcome(s) is/are recorded while under observation in this resource? Please select all that apply.

- Maternal outcomes
  - Thrombosis and/or thrombocytopenia syndrome
  - Antenatal hospitalization not including delivery
  - Disability
  - Gestational diabetes
  - Gestational hypertension
  - Premature rupture of membranes
  - Preterm labor
  - Preeclampsia / eclampsia
  - Post-partum hemorrhage
  - Antenatal hemorrhage
  - Corporeal infection
  - Spontaneous abortion / miscarriage / pregnancy loss (example: prior to 20 weeks gestation)
  - Maternal death (example: within 42 days of termination of pregnancy)
  - Late maternal death (example: >42 days – 1 year after termination of pregnancy)
  - Other

If you selected “maternal death” or “late maternal death”, please specify the cause of death:

- Direct
- Indirect

If you selected “other”, please specify: ______________________________________________

- Neonatal outcomes
  - Preterm birth
  - Small size for gestational age / restricted fetal growth
  - Stillbirth (death after 28 weeks of pregnancy but before birth)
  - Live birth
  - Congenital anomalies / birth defects
  - Death
  - Other

If “congenital anomalies / birth defects” is selected, please specify: ________________________

If neonatal “death” was selected, please specify the timeframe:

- Early neonatal (0-7 days)
- Late neonatal (8-28 days)
- Post neonatal (29 days – 1 year)

If you selected “other”, please specify: ______________________________________________

- Infant/child outcomes
  - Neonatal infections
  - Respiratory illness
  - Developmental outcomes
    - Specify (motor, cognitive, neurologic, autism, etc.): _____________________
  - Other

If you selected “other”, please specify: ______________________________________________

What is the duration of follow up? _______________________________________________

Resource start date: _________________________________________________________________

Resource end date (if applicable): _____________________________________________________

What is the current status of the resource?

- Open
- Closed

Who has access to this resource and its data? ____________________________________________

Do you participate in the running of this resource?

- Yes
- No

Do you contribute data to this resource?

- Yes
- No
- Not applicable

Are you a user of this resource?

- Yes
- No

Related publications/links: ___________________________________________________________

Anything else you’d like to share? _____________________________________________________

*File upload.* If available, please upload any relevant documents here from the resource, including templates, data collection forms, data dictionaries, data structures, publications, etc. These documents will help us understand the breadth of information that is captured in this resource.

PERLA Key Informant Interview

**Consent script**

Hello, my name is _____________ and I work at PATH, an international NGO working in health. We are conducting a landscape analysis in collaboration with WHO to identify available resources, including pregnancy exposure and surveillance registries, databases, surveys, and routinely collected data, that record exposure to medical products during pregnancy and maternal and perinatal outcomes in low- and middle-income countries (LMICs). You either filled out a survey about one or more resources that fit this description, or have been identified as someone who may have more information on these types of resources and may be interested in telling us more. This interview will take between 15-30 minutes and we will ask for your feedback and impressions on the resource(s) you’ve identified. Please keep in mind there are no right or wrong answers, and we are interested in your understanding of the resource as someone who maintains or interacts with these types of resources. We may record this session to help us later with our report. No video footage will be recorded, only audio. Participation is voluntary and if you would like to stop or not answer a question, you may do so at any time. Your name or identity will not be associated with the feedback you provide. Results may be compiled into a report, peer reviewed manuscript, or other communications materials that will be made publicly available. If we make reference to something you have said in our report, you would be referred to as “Participant #1, or #2, etc.” Please confirm your interest in participating in this session?

- Yes
- No

Thank you for agreeing to participate in this interview. Let’s get started. **(I will start the recording now.)**

This is an interview with participant _________.

**Interviewee information**

*For interviewer only: Please fill this section out before the interview and do not speak any identifying information over the recording. If any sections of their survey have been left blank, please include those as follow up questions.*

Unique ID. Please reference the sheet if they have already filled out a survey and use their unique ID here. If they did not fill out a survey, assign them a new unique ID. ____________________________

Name: _____________________________________________________________________________

Email: _____________________________________________________________________________

Organization: ________________________________________________________________________

Title: _______________________________________________________________________________

Did they complete a survey form?

- Yes
- No

**Resource information**

*For interviewers only: If “No” is selected under the previous question, all the questions from the survey will show up. If “Yes” is selected, the survey questions that have been answered will be skipped and go straight into the following questions.*

We are interested in understanding whether this resource is well equipped or well suited for its designed role or purpose. This could include the possibility of the resource to be combined with others for broader safety surveillance purposes and to understand its impact on supporting maternal health generally.

1. Can you tell me about the overall goals of the resource you have described to us?

*Probe: target population, intervention monitored, outcomes monitored, enrollment size, etc.*

1. How have findings from this resource been used? In particular, have they been useful decision-making?

*Probe: in public health? In regulation? For clinicians? For other healthcare bodies?*

1. What are the advantages of this resource?

*Probe: Comprehensiveness (outcomes or breadth of resources), coverage, timeliness, usefulness, accuracy, completeness .*

1. What are the most important gaps or needs of this resource?

*Probe on gaps: Comprehensiveness (outcomes or breadth of resources), coverage, timeliness, usefulness, accuracy, completeness.*

1. What are the challenges this resource faces in the current context?

*Probe on challenges: Cost, time requirements to maintain the resource, software limitations, etc.*

1. Could data from this resource be able to be combined with other health surveillance resources that are used in this region?

*Probe: Why/why not? Are there any challenges you foresee?*

*Probe: How could these systems link?*

1. Are you aware of any changes that will be made to this resource in the foreseeable future?
2. Is there anyone else you know who might have useful information about resources like the ones we discussed today?

That was my last question. Before we finish, do you have any questions or additional comments regarding the topics discussed during this interview?

Thank you for your time today. Your input is greatly appreciated.
