# Supplementary material for: Landscape analysis of pregnancy exposure registries in low- and middle-income countries: a scoping review
Source: BMJ Open. 2025 Oct 29;15(10):e097198. doi: 10.1136/bmjopen-2024-097198 (PMC12574391; doi:10.1136/bmjopen-2024-097198)
Supplement: online supplemental file 2 [file bmjopen-15-10-s002.docx]

**Supplemental Material for:**

Bhat, et al. Landscape Analysis of Pregnancy Exposure Registries in Low- and Middle-Income Countries: a Scoping Review

**Search Strategy by Database – page 1**

**Data Extraction Form – page 30**

**Key Informant Survey and Interview Form – page 40**

**Table S1. Key characteristics of identified pregnancy exposure registries and related resources – page 48**

**Search strategies by database**

PubMed

| Set # | Query | Results |
| --- | --- | --- |
| 132 | #130 NOT #131 | 2,016 |
| 131 | address[Publication Type] OR autobiography[Publication Type] OR bibliography[Publication Type] OR biography[Publication Type] OR comment[Publication Type] OR dictionary[Publication Type] OR directory[Publication Type] OR editorial[Publication Type] OR "expression of concern"[Publication Type] OR festschrift[Publication Type] OR historical article[Publication Type] OR interactive tutorial[Publication Type] OR lecture[Publication Type] OR news[Publication Type] OR newspaper article[Publication Type] OR portrait[Publication Type] OR video-audio media[Publication Type] OR webcast[Publication Type] | 2,050,653 |
| 130 | #128 NOT #129 | 2,048 |
| 129 | Animals[mesh] NOT Humans[mesh] | 5,016,862 |
| 128 | #126 AND #127 | 2,051 |
| 127 | ("2000"[Date - Publication] : "3000"[Date - Publication]) | 20,721,844 |
| 126 | #61 AND #125 | 2,597 |
| 125 | #62 OR #63 OR #64 OR #65 OR #66 OR #67 OR #68 OR #69 OR #70 OR #71 OR #72 OR #73 OR #74 OR #75 OR #76 OR #77 OR #78 OR #79 OR #80 OR #81 OR #82 OR #83 OR #84 OR #85 OR #86 OR #87 OR #88 OR #89 OR #90 OR #91 OR #92 OR #93 OR #94 OR #95 OR #96 OR #97 OR #98 OR #99 OR #100 OR #101 OR #102 OR #103 OR #104 OR #105 OR #106 OR #107 OR #108 OR #109 OR #110 OR #111 OR #112 OR #113 OR #114 OR #115 OR #116 OR #117 OR #118 OR #119 OR #120 OR #121 OR #122 OR #123 OR #124 | 7,704,889 |
| 124 | MNCH[Other Term] AND (maternal*[Other Term] OR newborn*[Other Term] OR child*[Other Term]) | 11 |
| 123 | MNCH[Title] AND (maternal*[Title] OR newborn*[Title] OR child*[Title]) | 22 |
| 122 | "Maternal Health"[mesh] | 2,142 |
| 121 | "Child Health"[mesh] | 4,447 |
| 120 | "Infant Health"[mesh] | 1,175 |
| 119 | (birth[Other Term] OR births[Other Term]) AND defect*[Other Term] | 1,029 |
| 118 | (birth[Title] OR births[Title]) AND defect*[Title] | 2,671 |
| 117 | (birth[Other Term] OR births[Other Term] OR matern*[Other Term] OR neonat*[Other Term] OR neo-nat*[Other Term] OR perinatal*[Other Term] OR peri-natal*[Other Term] OR peripartum[Other Term] OR "peri-partum"[Other Term] OR postnatal*[Other Term] OR post-natal*[Other Term] OR postpartum[Other Term] OR "post-partum"[Other Term] OR post-birth*[Other Term] OR pregnanc*[Other Term]) AND outcome*[Other Term] | 9,426 |
| 116 | (birth[Title] OR births[Title] OR matern*[Title] OR neonat*[Title] OR neo-nat*[Title] OR perinatal*[Title] OR peri-natal*[Title] OR peripartum[Title] OR "peri-partum"[Title] OR postnatal*[Title] OR post-natal*[Title] OR postpartum[Title] OR "post-partum"[Title] OR post-birth*[Title] OR pregnanc*[Title]) AND outcome*[Title] | 31,066 |
| 115 | Pregnancy Outcome[mesh] | 82,242 |
| 114 | (fetal[OT] OR foetal[OT] OR fetus*[OT] OR foetus*[OT] OR prenatal*[OT] OR pre-natal*[OT]) AND expos*[OT] | 2,033 |
| 113 | (fetal[Title] OR foetal[Title] OR fetus*[Title] OR foetus*[Title] OR prenatal*[Title] OR pre-natal*[Title]) AND expos*[Title] | 10,858 |
| 112 | "Prenatal Exposure Delayed Effects"[mesh] | 33,016 |
| 111 | (fetal[Other Term] OR foetal[Other Term] OR fetus*[Other Term] OR foetus*[Other Term]) AND develop*[Other Term] | 2,531 |
| 110 | (fetal[Title] OR foetal[Title] OR fetus*[Title] OR foetus*[Title]) AND develop*[Title] | 8,734 |
| 109 | "Fetal Development"[mesh:noexp] | 9,246 |
| 108 | antiretroviral*[Other Term] OR anti-retroviral*[Other Term] OR "anti-HIV"[Other Term] OR "anti-AIDS"[Other Term] OR "AIDS drug"[Other Term] OR "AIDS drugs"[Other Term] | 7,307 |
| 107 | (antiretroviral*[Title] OR anti-retroviral*[Title] OR "anti-HIV"[Title] OR "anti-AIDS"[Title] OR "AIDS drug"[Title] OR "AIDS drugs"[Title]) | 29,826 |
| 106 | "Anti-Retroviral Agents"[mesh] | 69,532 |
| 105 | "Zika Virus Infection/prevention and control"[MeSH] | 1,096 |
| 104 | "Streptococcal Infections/prevention and control"[MeSH:noexp] | 2,944 |
| 103 | "Respiratory Syncytial Virus Infections/prevention and control"[MeSH Terms] | 1,832 |
| 102 | "Hepatitis E/prevention and control"[MeSH Terms] | 346 |
| 101 | "Cytomegalovirus Infections/prevention and control"[MeSH Terms] | 3,130 |
| 100 | antimalarial*[Other Term] OR anti-malarial*[Other Term] | 2,577 |
| 99 | antimalarial*[Title] OR anti-malarial*[Title] | 8,562 |
| 98 | Antimalarials[mesh] | 28,226 |
| 97 | "Malaria/prevention and control"[MeSH Terms] | 17,528 |
| 96 | (maternal[Other Term] OR pregnan*[Other Term]) AND (immunisation*[Other Term] OR immunization*[Other Term]) | 754 |
| 95 | (maternal[Title] OR pregnan*[Title]) AND (immunisation*[Title] OR immunization*[Title]) | 1,153 |
| 94 | (antibod*[Other Term] OR anti-bod*[Other Term]) AND transfer*[Other Term] | 338 |
| 93 | (antibod*[Title] OR anti-bod*[Title]) AND transfer*[Title] | 1,620 |
| 92 | immunit*[Other Term] AND maternally-acqui*[Other Term] | 8 |
| 91 | immunit*[Title] AND maternally-acqui*[Title] | 8 |
| 90 | immunit*[Other Term] AND transfer*[Other Term] | 161 |
| 89 | immunit*[Title] AND transfer*[Title] | 882 |
| 88 | "Immunity, Maternally-Acquired"[mesh] | 5,842 |
| 87 | "Immunization Programs"[mesh:noexp] | 12,435 |
| 86 | Immunization[mesh:noexp] | 144,668 |
| 85 | vaccin*[ti] OR vaccin*[ot] | 220,223 |
| 84 | "Viral Vaccines"[mesh] | 132,729 |
| 83 | "Vaccines, Live, Unattenuated"[mesh] | 82 |
| 82 | "Vaccines, Synthetic"[mesh] | 31,589 |
| 81 | "Vaccines, Inactivated"[mesh:noexp] | 6,082 |
| 80 | "Vaccines, Combined"[mesh] | 10,562 |
| 79 | "Vaccines, Attenuated"[mesh] | 12,810 |
| 78 | "Protozoan Vaccines"[mesh] | 6,894 |
| 77 | "Bacterial Vaccines"[mesh] | 72,365 |
| 76 | Vaccines[mesh:noexp] | 25,746 |
| 75 | Vaccination[mesh] | 100,718 |
| 74 | GAIA[Text Word] AND (alignment[Text Word] OR immunisation[Text Word] OR immunization[Text Word] OR safety[Text Word] OR pregnan*[Text Word]) | 35 |
| 73 | "Global Alignment of Immunization Safety Assessment in Pregnancy"[tw] - Schema: all | 0 |
| 72 | AEFI[Other Term] AND adverse[Other Term] | 68 |
| 71 | AEFI[Title] AND adverse[Title] | 35 |
| 70 | adverse effect[Other Term] OR adverse effects[Other Term] OR adverse reaction[Other Term] OR adverse reactions[Other Term] OR adverse event[Other Term] OR adverse events[Other Term] OR adverse outcome[Other Term] OR adverse outcomes[Other Term] | 17,729 |
| 69 | adverse effect[Title] OR adverse effects[Title] OR adverse reaction[Title] OR adverse reactions[Title] OR adverse event[Title] OR adverse events[Title] OR adverse outcome[Title] OR adverse outcomes[Title] | 31,848 |
| 68 | safe[Other Term] OR safety[Other Term] OR side effect[Other Term] OR side effects[Other Term] OR undesirable effect[Other Term] OR undesirable effects[Other Term] OR treatment emergent[Other Term] OR tolerability[Other Term] OR toxicity[Other Term] OR adrs[Other Term] | 98,171 |
| 67 | safe[Title] OR safety[Title] OR side effect[Title] OR side effects[Title] OR undesirable effect[Title] OR undesirable effects[Title] OR treatment emergent[Title] OR tolerability[Title] OR toxicity[Title] OR adrs[Title] | 296,710 |
| 66 | ("adverse effects"[MeSH Subheading]) OR (Complications[MeSH Subheading]) OR ("drug effects"[MeSH Subheading]) | 6,963,724 |
| 65 | (drug[Title] OR drugs[Title] OR medicine[Title] OR medicines[Title] OR medication*[Title] OR pharmaceutical*[Title] OR pharma-ceutical*[Title]) AND expos*[Title] | 4,387 |
| 64 | "Drug-Related Side Effects and Adverse Reactions"[MeSH Terms] | 127,437 |
| 63 | (global[Title] OR international*[Title] OR world*[Title]) AND health*[Title] | 27,778 |
| 62 | Global Health[mesh] | 53,845 |
| 61 | #56 AND #60 | 8,450 |
| 60 | #57 OR #58 OR #59 | 1,104,126 |
| 59 | "high burden country"[Other Term] OR "high burden countries"[Other Term] OR "high-burden country"[Other Term] OR "high-burden countries"[Other Term] OR "countdown country"[Other Term] OR "countdown countries"[Other Term] | 10 |
| 58 | "high burden country"[Title/Abstract] OR "high burden countries"[Title/Abstract] OR "high-burden country"[Title/Abstract] OR "high-burden countries"[Title/Abstract] OR "countdown country"[Title/Abstract] OR "countdown countries"[Title/Abstract] | 633 |
| 57 | afghanistan[Text Word] OR albania[Text Word] OR algeria[Text Word] OR "american samoa"[Text Word] OR angola[Text Word] OR antigua[Text Word] OR barbuda[Text Word] OR argentina[Text Word] OR armenia[Text Word] OR armenian[Text Word] OR aruba[Text Word] OR azerbaijan[Text Word] OR bahrain[Text Word] OR bangladesh[Text Word] OR barbados[Text Word] OR belarus[Text Word] OR byelarus[Text Word] OR belorussia[Text Word] OR byelorussian[Text Word] OR belize[Text Word] OR "british honduras"[Text Word] OR benin[Text Word] OR dahomey[Text Word] OR bhutan[Text Word] OR bolivia[Text Word] OR "bosnia herzegovina"[Text Word] OR bosnia[Text Word] OR herzegovina[Text Word] OR botswana[Text Word] OR bechuanaland[Text Word] OR brazil[Text Word] OR brasil[Text Word] OR bulgaria[Text Word] OR "burkina faso"[Text Word] OR "burkina fasso"[Text Word] OR "upper volta"[Text Word] OR burundi[Text Word] OR urundi[Text Word] OR "cabo verde"[Text Word] OR "cape verde"[Text Word] OR cambodia[Text Word] OR kampuchea[Text Word] OR "khmer republic"[Text Word] OR cameroon[Text Word] OR cameron[Text Word] OR cameroun[Text Word] OR "central african republic"[Text Word] OR "ubangi shari"[Text Word] OR chad[Text Word] OR chile[Text Word] OR china[Text Word] OR colombia[Text Word] OR comoros[Text Word] OR "comoro islands"[Text Word] OR "iles comores"[Text Word] OR mayotte[Text Word] OR "democratic republic of the congo"[Text Word] OR "democratic republic congo"[Text Word] OR congo[Text Word] OR zaire[Text Word] OR "costa rica"[Text Word] OR "cote d’ivoire"[Text Word] OR "cote d’ ivoire"[Text Word] OR "cote divoire"[Text Word] OR "cote d ivoire"[Text Word] OR "ivory coast"[Text Word] OR croatia[Text Word] OR cuba[Text Word] OR cyprus[Text Word] OR "czech republic"[Text Word] OR czechoslovakia[Text Word] OR djibouti[Text Word] OR "french somaliland"[Text Word] OR dominica[Text Word] OR "dominican republic"[Text Word] OR ecuador[Text Word] OR egypt[Text Word] OR "united arab republic"[Text Word] OR "el salvador"[Text Word] OR "equatorial guinea"[Text Word] OR "spanish guinea"[Text Word] OR eritrea[Text Word] OR estonia[Text Word] OR eswatini[Text Word] OR swaziland[Text Word] OR ethiopia[Text Word] OR fiji[Text Word] OR gabon[Text Word] OR "gabonese republic"[Text Word] OR gambia[Text Word] OR "georgia (republic)"[Text Word] OR georgian[Text Word] OR ghana[Text Word] OR "gold coast"[Text Word] OR gibraltar[Text Word] OR greece[Text Word] OR grenada[Text Word] OR guam[Text Word] OR guatemala[Text Word] OR guinea[Text Word] OR "guinea bissau"[Text Word] OR guyana[Text Word] OR "british guiana"[Text Word] OR haiti[Text Word] OR hispaniola[Text Word] OR honduras[Text Word] OR hungary[Text Word] OR india[Text Word] OR indonesia[Text Word] OR timor[Text Word] OR iran[Text Word] OR iraq[Text Word] OR "isle of man"[Text Word] OR jamaica[Text Word] OR jordan[Text Word] OR kazakhstan[Text Word] OR kazakh[Text Word] OR kenya[Text Word] OR "democratic people’s republic of korea"[Text Word] OR "republic of korea"[Text Word] OR "north korea"[Text Word] OR "south korea"[Text Word] OR korea[Text Word] OR kosovo[Text Word] OR kyrgyzstan[Text Word] OR kirghizia[Text Word] OR kirgizstan[Text Word] OR "kyrgyz republic"[Text Word] OR kirghiz[Text Word] OR laos[Text Word] OR "lao pdr"[Text Word] OR "lao people's democratic republic"[Text Word] OR latvia[Text Word] OR lebanon[Text Word] OR lebanese republic[Text Word] OR lesotho[Text Word] OR basutoland[Text Word] OR liberia[Text Word] OR libya[Text Word] OR "libyan arab jamahiriya"[Text Word] OR lithuania[Text Word] OR macau[Text Word] OR macao[Text Word] OR republic of "north macedonia"[Text Word] OR macedonia[Text Word] OR madagascar[Text Word] OR "malagasy republic"[Text Word] OR malawi[Text Word] OR nyasaland[Text Word] OR malaysia[Text Word] OR "malay federation"[Text Word] OR "malaya federation"[Text Word] OR maldives[Text Word] OR "indian ocean islands"[Text Word] OR "indian ocean"[Text Word] OR mali[Text Word] OR malta[Text Word] OR micronesia[Text Word] OR "federated states of micronesia"[Text Word] OR kiribati[Text Word] OR "marshall islands"[Text Word] OR nauru[Text Word] OR "northern mariana islands"[Text Word] OR palau[Text Word] OR tuvalu[Text Word] OR mauritania[Text Word] OR mauritius[Text Word] OR mexico[Text Word] OR moldova[Text Word] OR moldovian[Text Word] OR mongolia[Text Word] OR montenegro[Text Word] OR morocco[Text Word] OR ifni[Text Word] OR mozambique[Text Word] OR "portuguese east africa"[Text Word] OR myanmar[Text Word] OR burma[Text Word] OR namibia[Text Word] OR nepal[Text Word] OR "netherlands antilles"[Text Word] OR nicaragua[Text Word] OR niger[Text Word] OR nigeria[Text Word] OR oman[Text Word] OR muscat[Text Word] OR pakistan[Text Word] OR panama[Text Word] OR "papua new guinea"[Text Word] OR "new guinea"[Text Word] OR paraguay[Text Word] OR peru[Text Word] OR philippines[Text Word] OR philipines[Text Word] OR phillipines[Text Word] OR phillippines[Text Word] OR poland[Text Word] OR "polish people's republic"[Text Word] OR portugal[Text Word] OR "portuguese republic"[Text Word] OR "puerto rico"[Text Word] OR romania[Text Word] OR russia[Text Word] OR "russian federation"[Text Word] OR ussr[Text Word] OR "soviet union"[Text Word] OR "union of soviet socialist republics"[Text Word] OR rwanda[Text Word] OR ruanda[Text Word] OR samoa[Text Word] OR "pacific islands"[Text Word] OR polynesia[Text Word] OR "samoan islands"[Text Word] OR "navigator island"[Text Word] OR "navigator islands"[Text Word] OR "sao tome and principe"[Text Word] OR "saudi arabia"[Text Word] OR senegal[Text Word] OR serbia[Text Word] OR seychelles[Text Word] OR "sierra leone"[Text Word] OR slovakia[Text Word] OR "slovak republic"[Text Word] OR slovenia[Text Word] OR melanesia[Text Word] OR "solomon island"[Text Word] OR "solomon islands"[Text Word] OR "norfolk island"[Text Word] OR "norfolk islands"[Text Word] OR somalia[Text Word] OR "south africa"[Text Word] OR "south sudan"[Text Word] OR "sri lanka"[Text Word] OR ceylon[Text Word] OR "saint kitts and nevis"[Text Word] OR "st. kitts and nevis"[Text Word] OR "saint lucia"[Text Word] OR "st. lucia"[Text Word] OR "saint Vincent and the grenadines"[Text Word] OR "saint vincent"[Text Word] OR "st. vincent"[Text Word] OR grenadines[Text Word] OR sudan[Text Word] OR suriname[Text Word] OR surinam[Text Word] OR "dutch guiana"[Text Word] OR "netherlands guiana"[Text Word] OR syria[Text Word] OR "syrian arab republic"[Text Word] OR tajikistan[Text Word] OR tadjikistan[Text Word] OR tadzhikistan[Text Word] OR tadzhik[Text Word] OR tanzania[Text Word] OR tanganyika[Text Word] OR thailand[Text Word] OR siam[Text Word] OR "timor leste"[Text Word] OR "east timor"[Text Word] OR togo[Text Word] OR "togolese republic"[Text Word] OR tonga[Text Word] OR "Trinidad and tobago"[Text Word] OR trinidad[Text Word] OR tobago[Text Word] OR tunisia[Text Word] OR turkey[Text Word] OR turkmenistan[Text Word] OR turkmen[Text Word] OR uganda[Text Word] OR ukraine[Text Word] OR uruguay[Text Word] OR uzbekistan[Text Word] OR uzbek[Text Word] OR vanuatu[Text Word] OR "new hebrides"[Text Word] OR venezuela[Text Word] OR vietnam[Text Word] OR "viet nam"[Text Word] OR "middle east"[Text Word] OR "west bank"[Text Word] OR gaza[Text Word] OR palestine[Text Word] OR yemen[Text Word] OR yugoslavia[Text Word] OR zambia[Text Word] OR zimbabwe[Text Word] OR "northern rhodesia"[Text Word] OR "global south"[Text Word] OR "africa south of the sahara"[Text Word] OR "sub-saharan africa"[Text Word] OR "subsaharan africa"[Text Word] OR "africa, central"[Text Word] OR "central africa"[Text Word] OR "africa, northern"[Text Word] OR "north africa"[Text Word] OR "northern africa"[Text Word] OR magreb[Text Word] OR maghrib[Text Word] OR sahara[Text Word] OR "africa, southern"[Text Word] OR "southern africa"[Text Word] OR "africa, eastern"[Text Word] OR "east africa"[Text Word] OR "eastern africa"[Text Word] OR "africa, western"[Text Word] OR "west africa"[Text Word] OR "western africa"[Text Word] OR "west indies"[Text Word] OR caribbean[Text Word] OR "central america"[Text Word] OR "latin america"[Text Word] OR "south and central america"[Text Word] OR "south america"[Text Word] OR "asia, central"[Text Word] OR "central asia"[Text Word] OR "asia, northern"[Text Word] OR "north asia"[Text Word] OR "northern asia"[Text Word] OR "asia, southeastern"[Text Word] OR "southeastern asia"[Text Word] OR "south eastern asia"[Text Word] OR "southeast asia"[Text Word] OR "south east asia"[Text Word] OR "asia, western"[Text Word] OR "western asia"[Text Word] OR "europe, eastern"[Text Word] OR "east europe"[Text Word] OR "eastern europe"[Text Word] OR "developing country"[Text Word] OR "developing countries"[Text Word] OR "developing nation"[Text Word] OR "developing nations"[Text Word] OR "developing population"[Text Word] OR "developing populations"[Text Word] OR "developing world"[Text Word] OR "less developed country"[Text Word] OR "less developed countries"[Text Word] OR "less developed nation"[Text Word] OR "less developed nations"[Text Word] OR "less developed population"[Text Word] OR "less developed populations"[Text Word] OR "less developed world"[Text Word] OR "lesser developed country"[Text Word] OR "lesser developed countries"[Text Word] OR "lesser developed nation"[Text Word] OR "lesser developed nations"[Text Word] OR "lesser developed population"[Text Word] OR "lesser developed populations"[Text Word] OR "lesser developed world"[Text Word] OR "under developed country"[Text Word] OR "under developed countries"[Text Word] OR "under developed nation"[Text Word] OR "under developed nations"[Text Word] OR "under developed population"[Text Word] OR "under developed populations"[Text Word] OR "under developed world"[Text Word] OR "underdeveloped country"[Text Word] OR "underdeveloped countries"[Text Word] OR "underdeveloped nation"[Text Word] OR "underdeveloped nations"[Text Word] OR "underdeveloped population"[Text Word] OR "underdeveloped populations"[Text Word] OR "underdeveloped world"[Text Word] OR "middle income country"[Text Word] OR "middle income countries"[Text Word] OR "middle income nation"[Text Word] OR "middle income nations"[Text Word] OR "middle income population"[Text Word] OR "middle income populations"[Text Word] OR "low income country"[Text Word] OR "low income countries"[Text Word] OR "low income nation"[Text Word] OR "low income nations"[Text Word] OR "low income population"[Text Word] OR "low income populations"[Text Word] OR "lower income country"[Text Word] OR "lower income countries"[Text Word] OR "lower income nation"[Text Word] OR "lower income nations"[Text Word] OR "lower income population"[Text Word] OR "lower income populations"[Text Word] OR "underserved country"[Text Word] OR "underserved countries"[Text Word] OR "underserved nation"[Text Word] OR "underserved nations"[Text Word] OR "underserved population"[Text Word] OR "underserved populations"[Text Word] OR "underserved world"[Text Word] OR "under served country"[Text Word] OR "under served countries"[Text Word] OR "under served nation"[Text Word] OR "under served nations"[Text Word] OR "under served population"[Text Word] OR "under served populations"[Text Word] OR "under served world"[Text Word] OR "deprived country"[Text Word] OR "deprived countries"[Text Word] OR "deprived nation"[Text Word] OR "deprived nations"[Text Word] OR "deprived population"[Text Word] OR "deprived populations"[Text Word] OR "deprived world"[Text Word] OR "poor country"[Text Word] OR "poor countries"[Text Word] OR "poor nation"[Text Word] OR "poor nations"[Text Word] OR "poor population"[Text Word] OR "poor populations"[Text Word] OR "poor world"[Text Word] OR "poorer country"[Text Word] OR "poorer countries"[Text Word] OR "poorer nation"[Text Word] OR "poorer nations"[Text Word] OR "poorer population"[Text Word] OR "poorer populations"[Text Word] OR "poorer world"[Text Word] OR "developing economy"[Text Word] OR "developing economies"[Text Word] OR "less developed economy"[Text Word] OR "less developed economics"[Text Word] OR "lesser developed economy"[Text Word] OR "lesser developed economies"[Text Word] OR "under developed economy"[Text Word] OR "under developed economies"[Text Word] OR "underdeveloped economy"[Text Word] OR "underdeveloped economies"[Text Word] OR "middle income economy"[Text Word] OR "middle income economies"[Text Word] OR "low income economy"[Text Word] OR "low income economies"[Text Word] OR "lower income economy"[Text Word] OR "lower income economies"[Text Word] OR "low gdp"[Text Word] OR "low gnp"[Text Word] OR "low gross domestic"[Text Word] OR "low gross national"[Text Word] OR "lower gdp"[Text Word] OR "lower gnp"[Text Word] OR "lower gross domestic"[Text Word] OR "lower gross national"[Text Word] OR lmic[Text Word] OR lmics[Text Word] OR "third world"[Text Word] OR "lami country"[Text Word] OR "lami countries"[Text Word] OR "transitional country"[Text Word] OR "transitional economies"[Text Word] OR "emerging economy"[Text Word] OR "emerging economies"[Text Word] OR "emerging nation"[Text Word] OR "emerging nations"[Text Word] | 1,103,822 |
| 56 | #1 OR #55 | 39,866 |
| 55 | #21 AND #54 | 39,859 |
| 54 | #22 OR #23 OR #24 OR #25 OR #26 OR #27 OR #28 OR #29 OR #30 OR #31 OR #32 OR #33 OR #34 OR #35 OR #36 OR #37 OR #38 OR #39 OR #40 OR #41 OR #42 OR #43 OR #44 OR #45 OR #46 OR #47 OR #48 OR #49 OR #50 OR #51 OR #52 OR #53 | 850,027 |
| 53 | "Product Surveillance, Postmarketing/statistics and numerical data"[MeSH Terms] | 2,361 |
| 52 | "Maternal Health Services/statistics and numerical data"[MeSH Terms] | 7,290 |
| 51 | "Maternal Exposure/statistics and numerical data"[MeSH Terms] | 1,123 |
| 50 | pharmacovigilan*[ti] OR pharmaco-vigilan*[ti] OR pharmacovigilan*[ot] OR pharmaco-vigilan*[ot] | 3,751 |
| 49 | "Product Surveillance, Postmarketing"[mesh:noexp] | 7,553 |
| 48 | "data system"[ti] OR "data systems"[ti] OR "information system"[ti] OR "information systems"[ti] OR "data system"[ot] OR "data systems"[ot] OR "information system"[ot] OR "information systems"[ot] | 13,581 |
| 47 | "Health Information Systems"[mesh] | 1,546 |
| 46 | (decision*[ti] AND support*[ti] AND clinical*[ti]) OR (decision*[ot] AND support*[ot] AND clinical*[ot]) | 4,467 |
| 45 | "Decision Support Systems, Clinical"[mesh] | 9,106 |
| 44 | "Databases, Factual"[mesh] | 163,862 |
| 43 | "Databases as Topic"[mesh:noexp] | 9,688 |
| 42 | surveillance*[ti] OR surveillance*[ot] | 61,304 |
| 41 | "Population Surveillance"[mesh] | 74,026 |
| 40 | survey*[ti] OR survey*[ot] | 189,840 |
| 39 | "Health Surveys"[mesh:noexp] | 66,266 |
| 38 | "Health Care Surveys"[MeSH Major Topic] | 10,167 |
| 37 | "Surveys and Questionnaires"[MeSH Major Topic:noexp] | 49,876 |
| 36 | registry[ot] OR registries[ot] OR eregistr*[ot] OR "e-registry"[ot] OR "e-registries"[ot] | 8,509 |
| 35 | registry[ti] OR registries[ti] OR eregistr*[ti] OR "e-registry"[ti] OR "e-registries"[ti] | 34,311 |
| 34 | Registries[mesh:noexp] | 104,749 |
| 33 | (preliminary[ot] OR "pilot project"[ot] OR "pilot projects"[ot]) AND data[ot] | 35 |
| 32 | (preliminary[ti] OR "pilot project"[ti] OR "pilot projects"[ti]) AND data[ti] | 2,947 |
| 31 | "Preliminary Data"[mesh] | 724 |
| 30 | "focus group"[ti] OR "focus groups"[ti] OR "focus group"[ot] OR "focus groups"[ot] | 6,048 |
| 29 | "Focus Groups"[mesh] | 34,384 |
| 28 | databas*[ot] OR "data base"[ot] OR "data bases"[ot] OR databank*[ot] OR "data bank"[ot] OR "data banks"[ot] OR dataset*[ot] OR "data set"[ot] OR "data sets"[ot] | 14,502 |
| 27 | databas*[ti] OR "data base"[ti] OR "data bases"[ti] OR databank*[ti] OR "data bank"[ti] OR "data banks"[ti] OR dataset*[ti] OR "data set"[ti] OR "data sets"[ti] | 55,119 |
| 26 | "Datasets as Topic"[mesh] | 7,252 |
| 25 | data[ot] AND (accumulat*[ot] OR accura*[ot] OR assembl*[ot] OR captur*[ot] OR collect*[ot] OR compil*[ot] OR coordinat*[ot] OR co-ordinat*[ot] OR gather*[ot] OR hub[ot] OR hubs[ot]) | 5,205 |
| 24 | data[ti] AND (accumulat*[ti] OR accura*[ti] OR assembl*[ti] OR captur*[ti] OR collect*[ti] OR compil*[ti] OR coordinat*[ti] OR co-ordinat*[ti] OR gather*[ti] OR hub[ti] OR hubs[ti]) | 11,365 |
| 23 | "Data Accuracy"[mesh] | 3,726 |
| 22 | "Data Collection"[mesh:noexp] | 91,761 |
| 21 | #2 OR #3 OR #4 OR #5 OR #6 OR #7 OR #8 OR #9 OR #10 OR #11 OR #12 OR #13 OR #14 OR #15 OR #16 OR #17 OR #18 OR #19 OR #20 | 1,067,642 |
| 20 | maternal[ot] AND (fetal[ot] OR fetus[ot] OR foetal[ot] OR foetus[ot]) | 3,848 |
| 19 | (maternal*[ot] OR maternity[ot]) AND (care[ot] OR health*[ot] OR service[ot] OR services[ot]) | 11,210 |
| 18 | (maternal*[ot] or maternity[ot]) AND (contact*[ot] or expos*[ot]) | 601 |
| 17 | perinatal*[ot] OR "peri-natal"[ot] OR "peri-natally"[ot] OR peripartum[ot] or "peri-partum"[ot] | 7,552 |
| 16 | prenatal*[ot] OR antenatal*[ot] OR "ante-natal"[ot] OR "ante-natally"[ot] OR antepartum[ot] or "ante-partum"[ot] | 15,862 |
| 15 | maternal[ti] AND (fetal[ti] OR fetus[ti] OR foetal[ti] OR foetus[ti]) | 13,964 |
| 14 | (maternal*[ti] OR maternity[ti]) AND (care[ti] OR health*[ti] OR service[ti] OR services[ti]) | 13,467 |
| 13 | "Maternal Health Services"[mesh:noexp] | 15,617 |
| 12 | (maternal*[ti] or maternity[ti]) AND (contact*[ti] or expos*[ti]) | 3,847 |
| 11 | "Maternal Exposure"[mesh] | 10,689 |
| 10 | perinatal*[ti] OR "peri-natal"[ti] OR "peri-natally"[ti] OR peripartum[ti] or "peri-partum"[ti] | 30,982 |
| 9 | "Perinatal Care"[mesh] | 11,334 |
| 8 | prenatal*[ti] OR antenatal*[ti] OR "ante-natal"[ti] OR "ante-natally"[ti] OR antepartum[ti] or "ante-partum"[ti] | 59,414 |
| 7 | "Prenatal Care"[mesh] | 31,249 |
| 6 | pregnan*[ti] | 252,201 |
| 5 | "Pregnancy Trimesters"[mesh] | 43,874 |
| 4 | "Pregnant Women"[mesh] | 12,334 |
| 3 | "Pregnancy Complications"[mesh] | 460,551 |
| 2 | Pregnancy[mesh] | 970,546 |
| 1 | "pregnancy exposure database" [tw] OR "pregnancy exposure databases" [tw] OR "pregnancy exposure data base" [tw] OR "pregnancy exposure data bases" [tw] OR "pregnancy exposure registry" [tw] OR "pregnancy exposure registries" [tw] | 34 |

Embase

--------------------------------------------------------------------------------

1 ("pregnancy exposure database" or "pregnancy exposure databases" or "pregnancy exposure data base" or "pregnancy exposure data bases" or "pregnancy exposure registry" or "pregnancy exposure registries").tw,kw,kf. (113)

2 exp pregnancy/ (727815)

3 exp pregnancy complication/ (141579)

4 exp named groups by pregnancy/ (130431)

5 pregnan*.ti,kw,kf. (329712)

6 exp prenatal care/ (167165)

7 (prenatal* or antenatal* or "ante-natal" or "ante-natally" or antepartum or "ante-partum").ti,kw,kf. (87488)

8 exp perinatal care/ (65235)

9 (perinatal* or peri-natal* or peripartum or "peri-partum").ti,kw,kf. (46195)

10 maternal exposure/ (3606)

11 ((maternal* or maternity) and (contact* or expos*)).ti,kw,kf. (6357)

12 maternal health service/ (2454)

13 ((maternal* or maternity) and (care or health* or service or services)).ti,kw,kf. (22738)

14 (maternal and (fetal or fetus or foetal or foetus)).ti,kw,kf. (20741)

15 or/2-14 [PREGNANCY] (1035932)

16 *information processing/ (37078)

17 data aggregation/ (374)

18 data accuracy/ (1596)

19 (data and (accumulat* or accura* or assembl* or captur* or collect* or compil* or coordinat* or co-ordinat* or gather* or hub or hubs)).ti,kw,kf. (18259)

20 (databas* or "data base" or "data bases" or databank* or "data bank" or "data banks" or dataset* or "data set" or "data sets").ti,kw,kf. (86633)

21 (focus group or focus groups).ti,kw,kf. (7608)

22 preliminary data/ (31472)

23 ((preliminary or pilot project or pilot projects) and data).ti,kw,kf. (5481)

24 exp register/ (180900)

25 (registry or registries or eregistr* or "e-registry" or "e-registries").ti,kw,kf. (68347)

26 questionnaire/ (814172)

27 *health care survey/ (2606)

28 health survey/ (213987)

29 survey*.ti,kw,kf. (220889)

30 population surveillance/ (117)

31 surveillance*.ti,kw,kf. (82221)

32 data base/ (248533)

33 factual database/ (27912)

34 clinical decision support system/ (4716)

35 (decision* and support* and clinical*).ti,kw,kf. (5904)

36 medical information system/ (22591)

37 ("data system" or "data systems" or "information system" or "information systems").ti,kw,kf. (18222)

38 exp postmarketing surveillance/ (37924)

39 (pharmacovigilan* or pharmaco-vigilan*).ti,kw,kf. (7417)

40 or/16-39 [DATA COLLECTION, REGISTRIES] (1815231)

41 15 and 40 [PREGNANCY - DATA COLLECTION, REGISTRIES] (71373)

42 1 or 41 [PERs, PREGNANCY - DATA COLLECTION, REGISTRIES] (71381)

43 (afghanistan or albania or algeria or american samoa or angola or "antigua and barbuda" or antigua or barbuda or argentina or armenia or armenian or aruba or azerbaijan or bahrain or bangladesh or barbados or republic of belarus or belarus or byelarus or belorussia or byelorussian or belize or british honduras or benin or dahomey or bhutan or bolivia or "bosnia and herzegovina" or bosnia or herzegovina or botswana or bechuanaland or brazil or brasil or bulgaria or burkina faso or burkina fasso or upper volta or burundi or urundi or cabo verde or cape verde or cambodia or kampuchea or khmer republic or cameroon or cameron or cameroun or central african republic or ubangi shari or chad or chile or china or colombia or comoros or comoro islands or iles comores or mayotte or democratic republic of the congo or democratic republic congo or congo or zaire or costa rica or "cote d’ivoire" or "cote d’ ivoire" or cote divoire or cote d ivoire or ivory coast or croatia or cuba or cyprus or czech republic or czechoslovakia or djibouti or french somaliland or dominica or dominican republic or ecuador or egypt or united arab republic or el salvador or equatorial guinea or spanish guinea or eritrea or estonia or eswatini or swaziland or ethiopia or fiji or gabon or gabonese republic or gambia or "georgia (republic)" or georgian or ghana or gold coast or gibraltar or greece or grenada or guam or guatemala or guinea or guinea bissau or guyana or british guiana or haiti or hispaniola or honduras or hungary or india or indonesia or timor or iran or iraq or isle of man or jamaica or jordan or kazakhstan or kazakh or kenya or "democratic people’s republic of korea" or republic of korea or north korea or south korea or korea or kosovo or kyrgyzstan or kirghizia or kirgizstan or kyrgyz republic or kirghiz or laos or lao pdr or "lao people's democratic republic" or latvia or lebanon or lebanese republic or lesotho or basutoland or liberia or libya or libyan arab jamahiriya or lithuania or macau or macao or republic of north macedonia or macedonia or madagascar or malagasy republic or malawi or nyasaland or malaysia or malay federation or malaya federation or maldives or indian ocean islands or indian ocean or mali or malta or micronesia or federated states of micronesia or kiribati or marshall islands or nauru or northern mariana islands or palau or tuvalu or mauritania or mauritius or mexico or moldova or moldovian or mongolia or montenegro or "montenegro (republic)" or morocco or ifni or mozambique or portuguese east africa or myanmar or burma or namibia or nepal or netherlands antilles or nicaragua or niger or nigeria or oman or muscat or pakistan or panama or papua new guinea or new guinea or paraguay or peru or philippines or philipines or phillipines or phillippines or poland or "polish people's republic" or portugal or portuguese republic or puerto rico or romania or russia or russian federation or ussr or soviet union or union of soviet socialist republics or rwanda or ruanda or samoa or pacific islands or polynesia or samoan islands or navigator island or navigator islands or "sao tome and principe" or saudi arabia or senegal or serbia or seychelles or sierra leone or slovakia or slovak republic or slovenia or melanesia or solomon island or solomon islands or norfolk island or norfolk islands or somalia or south africa or south sudan or sri lanka or ceylon or "saint kitts and nevis" or "st. kitts and nevis" or saint lucia or "st. lucia" or "saint vincent and the grenadines" or saint vincent or "st. vincent" or grenadines or sudan or suriname or surinam or dutch guiana or netherlands guiana or syria or syrian arab republic or tajikistan or tadjikistan or tadzhikistan or tadzhik or tanzania or tanganyika or thailand or siam or timor leste or east timor or togo or togolese republic or tonga or "trinidad and tobago" or trinidad or tobago or tunisia or "turkey (republic)" or turkey or turkmenistan or turkmen or uganda or ukraine or uruguay or uzbekistan or uzbek or vanuatu or new hebrides or venezuela or vietnam or viet nam or middle east or west bank or gaza or palestine or yemen or yugoslavia or zambia or zimbabwe or northern rhodesia or global south or africa south of the sahara or "sub saharan africa" or subsaharan africa or africa, central or central africa or africa, northern or north africa or northern africa or magreb or maghrib or sahara or africa, southern or southern africa or africa, eastern or east africa or eastern africa or africa, western or west africa or western africa or west indies or indian ocean islands or caribbean region or caribbean islands or caribbean or central america or latin america or "south and central america" or south america or asia, central or central asia or asia, northern or north asia or northern asia or asia, southeastern or southeastern asia or south eastern asia or southeast asia or south east asia or asia, western or western asia or europe, eastern or east europe or eastern europe or developing country or developing countries or developing nation? or developing population? or developing world or less developed countr* or less developed nation? or less developed population? or less developed world or lesser developed countr* or lesser developed nation? or lesser developed population? or lesser developed world or under developed countr* or under developed nation? or under developed population? or under developed world or underdeveloped countr* or underdeveloped nation? or underdeveloped population? or underdeveloped world or middle income countr* or middle income nation? or middle income population? or low income countr* or low income nation? or low income population? or lower income countr* or lower income nation? or lower income population? or underserved countr* or underserved nation? or underserved population? or underserved world or under served countr* or under served nation? or under served population? or under served world or deprived countr* or deprived nation? or deprived population? or deprived world or poor countr* or poor nation? or poor population? or poor world or poorer countr* or poorer nation? or poorer population? or poorer world or developing econom* or less developed econom* or lesser developed econom* or under developed econom* or underdeveloped econom* or middle income econom* or low income econom* or lower income econom* or low gdp or low gnp or low gross domestic or low gross national or lower gdp or lower gnp or lower gross domestic or lower gross national or lmic or lmics or third world or lami countr* or transitional countr* or emerging economies or emerging nation?).ti,ab,sh,kw. (2517925)

44 ("high burden country" or "high burden countries" or "high-burden country" or "high-burden countries" or "countdown country" or "countdown countries").ti,ab,kw,kf. (808)

45 43 or 44 [LMICs] (2518149)

46 42 and 45 [PERs, PREGNANCY - DATA COLLECTION, REGISTRIES - LMICs] (18846)

47 global health/ (16219)

48 ((global or international* or world*) and health*).ti,kw,kf. (45947)

49 exp adverse drug reaction/ (586248)

50 ((drug or drugs or medicine or medicines or medication* or pharmaceutical* or pharma-ceutical*) and expos*).ti,kw,kf. (9329)

51 (ae or co).fs. (2915988)

52 (safe or safety or side effect or side effects or undesirable effect or undesirable effects or treatment emergent or tolerability or toxicity or adrs).ti,kw,kf. (514988)

53 (adverse effect or adverse effects or adverse reaction or adverse reactions or adverse event or adverse events or adverse outcome or adverse outcomes).ti,kw,kf. (70731)

54 (AEFI and adverse).ti,kw,kf. (157)

55 "Global Alignment of Immuni#ation Safety Assessment in Pregnancy".tw,kw,kf. (20)

56 (GAIA and (alignment or immuni#ation or safety or pregnan*)).tw,kw,kf. (71)

57 exp vaccination/ (205483)

58 vaccine/ (67719)

59 exp bacterial vaccine/ or exp cell-based vaccine/ or conjugate vaccine/ or edible vaccine/ or exp fungus vaccine/ or exp inactivated vaccine/ or live vaccine/ or exp meningitis vaccine/ or exp nucleic acid vaccine/ or exp parasite vaccine/ or exp peptide vaccine/ or protein vaccine/ or exp subunit vaccine/ or exp toxoid vaccine/ or exp vector vaccine/ or virosome vaccine/ or exp virus vaccine/ (304544)

60 vaccin*.ti,kw,kf. (251220)

61 immunization/ (104541)

62 passive immunization/ (12623)

63 (immunit* and transfer*).ti,kw,kf. (1262)

64 (immunit* and (maternally-acqui* or passive*)).ti,kw,kf. (766)

65 ((antibod* or anti-bod*) and transfer*).ti,kw,kf. (2375)

66 ((maternal or pregnan*) and immuni#ation*).ti,kw,kf. (1766)

67 exp malaria/pc [Prevention] (14654)

68 exp antimalarial agent/ (161880)

69 (antimalarial* or anti-malarial*).ti,kw,kf. (11586)

70 exp cytomegalovirus infection/pc [Prevention] (5146)

71 respiratory syncytial virus infection/pc [Prevention] (1057)

72 exp Streptococcus infection/pc [Prevention] (10568)

73 exp Zika fever/pc [Prevention] (780)

74 exp antiretrovirus agent/ (216196)

75 (antiretroviral* or anti-retroviral* or "anti-HIV" or "anti-AIDS" or "AIDS drug" or "AIDS drugs").ti,kw,kf. (46286)

76 exp fetus development/ (29871)

77 ((fetal or foetal or fetus* or foetus*) and develop*).ti,kw,kf. (14926)

78 exp prenatal exposure/ (37294)

79 ((fetal or foetal or fetus* or foetus* or prenatal* or pre-natal*) and expos*).ti,kw,kf. (16619)

80 pregnancy outcome/ (72177)

81 ((birth or births or matern* or neonat* or neo-nat* or perinatal* or peri-natal*or peripartum or "peri-partum" or postnatal* or post-natal* or postpartum or "post-partum" or post-birth* or pregnanc*) and outcome*).ti,kw,kf. (55800)

82 ((birth or births) and defect*).ti,kw,kf. (4849)

83 child health/ (32206)

84 maternal welfare/ (15842)

85 (MNCH and (maternal* or newborn* or child*)).ti,kw,kf. (49)

86 or/47-85 [DRUGS, VACCINES, SAFETY, OUTCOMES] (4592324)

87 46 and 86 [PERs, PREGNANCY - DATA COLLECTION, REGISTRIES - LMICs - DRUGS, VACCINES, SAFETY, OUTCOMES] (5663)

88 exp animal/ or exp animal experimentation/ or exp animal model/ or exp animal experiment/ or nonhuman/ or exp vertebrate/ (30677570)

89 exp human/ or exp human experimentation/ or exp human experiment/ (23737031)

90 88 not 89 (6941713)

91 87 not 90 [ANIMAL-ONLY REMOVED] (5643)

92 editorial.pt. (729310)

93 91 not 92 [EDITORIALS REMOVED] (5614)

94 limit 93 to yr="2000-current" (5299)

***************************

CINAHL

| # | Query | Results |
| --- | --- | --- |
| S92 | S90 and S91 | 878 |
| S91 | DT 2000 - 2022 | 7,386,209 |
| S90 | S88 NOT S89 | 885 |
| S89 | PT editorial or opinion or commentary | 684,750 |
| S88 | S45 AND S87 | 891 |
| S87 | S46 OR S47 OR S48 OR S49 OR S50 OR S51 OR S52 OR S53 OR S54 OR S55 OR S56 OR S57 OR S58 OR S59 OR S60 OR S61 OR S62 OR S63 OR S64 OR S65 OR S66 OR S67 OR S68 OR S69 OR S70 OR S71 OR S72 OR S73 OR S74 OR S75 OR S76 OR S77 OR S78 OR S79 OR S80 OR S81 OR S82 OR S83 OR S84 OR S85 OR S86 | 354,157 |
| S86 | TI (MNCH AND (maternal* OR newborn* OR child*)) | 17 |
| S85 | (MH "Maternal-Child Health") | 3,727 |
| S84 | (MH "Child Health") | 17,073 |
| S83 | TI (birth OR births) and defect* | 969 |
| S82 | TI (birth OR births OR matern* OR neonat* OR neo-nat* OR perinatal* OR peri-natal* OR peripartum OR "peri-partum" OR postnatal* OR post-natal* OR postpartum OR "post-partum" OR post-birth* OR pregnanc*) AND outcome* | 17,343 |
| S81 | (MH "Pregnancy Outcomes") | 27,127 |
| S80 | TI (fetal OR foetal OR fetus* OR foetus* or prenatal* or pre-natal*) AND expos* | 2,603 |
| S79 | (MH "Prenatal Exposure Delayed Effects") | 6,314 |
| S78 | TI (fetal OR foetal OR fetus* OR foetus*) AND develop* | 1,028 |
| S77 | (MH "Fetal Development") | 7,851 |
| S76 | TI antiretroviral* OR anti-retroviral* OR "anti-HIV" OR "anti-AIDS" OR "AIDS drug" OR "AIDS drugs" | 9,126 |
| S75 | (MH "Anti-Retroviral Agents+") | 24,911 |
| S74 | (MH "Zika Virus Infections/PC") | 168 |
| S73 | (MH "Streptococcal Infections+/PC") | 2,280 |
| S72 | (MH "Respiratory Syncytial Virus Infections/PC") | 583 |
| S71 | (MH "Hepatitis E/PC") | 69 |
| S70 | (MH "Cytomegalovirus Infections+/PC") | 509 |
| S69 | TI antimalarial* or (anti W0 malarial*) | 714 |
| S68 | (MH "Antimalarials+") | 8,320 |
| S67 | (MH "Malaria/PC") | 3,293 |
| S66 | TI (maternal* or pregnan*) and immuni?ation* | 253 |
| S65 | TI ((antibod* or (anti W0 bod*)) and transfer*) | 79 |
| S64 | TI immunit* AND (passive* or "maternally-acquired") | 13 |
| S63 | TI immunit* AND transfer* | 16 |
| S62 | (MH "Immunity, Maternally Acquired") | 232 |
| S61 | (MH "Immunization Programs") | 6,426 |
| S60 | (MH "Viral Vaccines+") | 36,206 |
| S59 | (MH "Vaccines, Combined+") | 3,562 |
| S58 | (MH "Toxoids+") | 2,372 |
| S57 | (MH "Bacterial Vaccines+") | 10,296 |
| S56 | (MH "Vaccines") | 9,533 |
| S55 | (MH "Immunization+") | 31,914 |
| S54 | TI ( GAIA AND (alignment or immuni?ation or safety or pregnan*) ) OR AB ( GAIA AND (alignment or immuni?ation or safety or pregnan*) ) | 6 |
| S53 | TI ( "Global Alignment of Immunisation Safety Assessment in Pregnancy" or "Global Alignment of Immunization Safety Assessment in Pregnancy" ) OR AB ( "Global Alignment of Immunisation Safety Assessment in Pregnancy" or "Global Alignment of Immunization Safety Assessment in Pregnancy" ) | 0 |
| S52 | TI AEFI and adverse | 5 |
| S51 | TI "adverse effect" or "adverse effects" or "adverse reaction" or "adverse reactions" or "adverse event" or "adverse events" or "adverse outcome" or "adverse outcomes" | 12,615 |
| S50 | TI safe or safety or "side effect" or "side effects" or "undesirable effect" or "undesirable effects" or "treatment emergent" or tolerability or toxicity or adrs | 113,937 |
| S49 | TI (drug or drugs or medicine or medicines or medication* or pharmaceutical* or pharma-ceutical*) AND expos* | 1,420 |
| S48 | (MH "Adverse Drug Event") | 16,207 |
| S47 | TI (global or international* or world*) and health* | 22,194 |
| S46 | (MH "World Health") | 29,391 |
| S45 | S39 AND S44 | 4,402 |
| S44 | S40 OR S41 OR S42 OR S43 | 379,212 |
| S43 | TI ( "high burden country" or "high burden countries" or "high-burden country" or "high-burden countries" or "countdown country" or "countdown countries" ) OR AB ( "high burden country" or "high burden countries" or "high-burden country" or "high-burden countries" or "countdown country" or "countdown countries" ) | 188 |
| S42 | TI ( "developing country" or "developing countries" or "developing nation" or "developing nations" or "developing population" or "developing populations" or "developing world" or "less developed country" or "less developed countries" or "less developed nation" or "less developed nations" or "less developed population" or "less developed populations" or "less developed world" or "lesser developed country" or "lesser developed countries" or "lesser developed nation" or "lesser developed nations" or "lesser developed population" or "lesser developed populations" or "lesser developed world" or "under developed country" or "under developed countries" or "under developed nation" or "under developed nations" or "under developed population" or "under developed populations" or "under developed world" or "underdeveloped country" or "underdeveloped countries" or "underdeveloped nation" or "underdeveloped nations" or "underdeveloped population" or "underdeveloped populations" or "underdeveloped world" or "middle income country" or "middle income countries" or "middle income nation" or "middle income nations" or "middle income population" or "middle income populations" or "low income country" or "low income countries" or "low income nation" or "low income nations" or "low income population" or "low income populations" or "lower income country" or "lower income countries" or "lower income nation" or "lower income nations" or "lower income population" or "lower income populations" or "underserved country" or "underserved countries" or "underserved nation" or "underserved nations" or "underserved population" or "underserved populations" or "underserved world" or "under served country" or "under served countries" or "under served nation" or "under served nations" or "under served population" or "under served populations" or "under served world" or "deprived country" or "deprived countries" or "deprived nation" or "deprived nations" or "deprived population" or "deprived populations" or "deprived world" or "poor country" or "poor countries" or "poor nation" or "poor nations" or "poor population" or "poor populations" or "poor world" or "poorer country" or "poorer countries" or "poorer nation" or "poorer nations" or "poorer population" or "poorer populations" or "poorer world" or "developing economy" or "developing economies" or "less developed economy" or "less developed economics" or "lesser developed economy" or "lesser developed economies" or "under developed economy" or "under developed economies" or "underdeveloped economy" or "underdeveloped economies" or "middle income economy" or "middle income economies" or "low income economy" or "low income economies" or "lower income economy" or "lower income economies" or "low gdp" or "low gnp" or "low gross domestic" or "low gross national" or "lower gdp" or "lower gnp" or "lower gross domestic" or "lower gross national" or lmic or lmics or "third world" or "lami country" or "lami countries" or "transitional country" or "transitional economies" or "emerging economy" or "emerging economies" or "emerging nation" or "emerging nations" ) OR AB ( "developing country" or "developing countries" or "developing nation" or "developing nations" or "developing population" or "developing populations" or "developing world" or "less developed country" or "less developed countries" or "less developed nation" or "less developed nations" or "less developed population" or "less developed populations" or "less developed world" or "lesser developed country" or "lesser developed countries" or "lesser developed nation" or "lesser developed nations" or "lesser developed population" or "lesser developed populations" or "lesser developed world" or "under developed country" or "under developed countries" or "under developed nation" or "under developed nations" or "under developed population" or "under developed populations" or "under developed world" or "underdeveloped country" or "underdeveloped countries" or "underdeveloped nation" or "underdeveloped nations" or "underdeveloped population" or "underdeveloped populations" or "underdeveloped world" or "middle income country" or "middle income countries" or "middle income nation" or "middle income nations" or "middle income population" or "middle income populations" or "low income country" or "low income countries" or "low income nation" or "low income nations" or "low income population" or "low income populations" or "lower income country" or "lower income countries" or "lower income nation" or "lower income nations" or "lower income population" or "lower income populations" or "underserved country" or "underserved countries" or "underserved nation" or "underserved nations" or "underserved population" or "underserved populations" or "underserved world" or "under served country" or "under served countries" or "under served nation" or "under served nations" or "under served population" or "under served populations" or "under served world" or "deprived country" or "deprived countries" or "deprived nation" or "deprived nations" or "deprived population" or "deprived populations" or "deprived world" or "poor country" or "poor countries" or "poor nation" or "poor nations" or "poor population" or "poor populations" or "poor world" or "poorer country" or "poorer countries" or "poorer nation" or "poorer nations" or "poorer population" or "poorer populations" or "poorer world" or "developing economy" or "developing economies" or "less developed economy" or "less developed economics" or "lesser developed economy" or "lesser developed economies" or "under developed economy" or "under developed economies" or "underdeveloped economy" or "underdeveloped economies" or "middle income economy" or "middle income economies" or "low income economy" or "low income economies" or "lower income economy" or "lower income economies" or "low gdp" or "low gnp" or "low gross domestic" or "low gross national" or "lower gdp" or "lower gnp" or "lower gross domestic" or "lower gross national" or lmic or lmics or "third world" or "lami country" or "lami countries" or "transitional country" or "transitional economies" or "emerging economy" or "emerging economies" or "emerging nation" or "emerging nations" ) | 39,219 |
| S41 | TI ( mali or malta or micronesia or "federated states of micronesia" or kiribati or "marshall islands" or nauru or "northern mariana islands" or palau or tuvalu or mauritania or mauritius or mexico or moldova or moldovian or mongolia or montenegro or morocco or ifni or mozambique or "portuguese east africa" or myanmar or burma or namibia or nepal or "netherlands antilles" or nicaragua or niger or nigeria or oman or muscat or pakistan or panama or "papua new guinea" or "new guinea" or paraguay or peru or philippines or philipines or phillipines or phillippines or poland or "polish people's republic" or portugal or "portuguese republic" or "puerto rico" or romania or russia or "russian federation" or ussr or "soviet union" or "union of soviet socialist republics" or rwanda or ruanda or samoa or "pacific islands" or polynesia or "samoan islands" or "navigator island" or "navigator islands" or "sao tome and principe" or "saudi arabia" or senegal or serbia or seychelles or "sierra leone" or slovakia or "slovak republic" or slovenia or melanesia or "solomon island" or "solomon islands" or "norfolk island" or "norfolk islands" or somalia or "south africa" or "south sudan" or "sri lanka" or ceylon or "saint kitts and nevis" or "st. kitts and nevis" or "saint lucia" or "st. lucia" or "saint Vincent and the grenadines" or "saint vincent" or "st. vincent" or grenadines or sudan or suriname or surinam or "dutch guiana" or "netherlands guiana" or syria or "syrian arab republic" or tajikistan or tadjikistan or tadzhikistan or tadzhik or tanzania or tanganyika or thailand or siam or "timor leste" or "east timor" or togo or "togolese republic" or tonga or "Trinidad and tobago" or trinidad or tobago or tunisia or turkey or turkmenistan or turkmen or uganda or ukraine or uruguay or uzbekistan or uzbek or vanuatu or "new hebrides" or venezuela or vietnam or "viet nam" or "middle east" or "west bank" or gaza or palestine or yemen or yugoslavia or zambia or zimbabwe or "northern rhodesia" or "global south" or "africa south of the sahara" or "sub-saharan africa" or "subsaharan africa" or "africa, central" or "central africa" or "africa, northern" or "north africa" or "northern africa" or magreb or maghrib or sahara or "africa, southern" or "southern africa" or "africa, eastern" or "east africa" or "eastern africa" or "africa, western" or "west africa" or "western africa" or "west indies" or caribbean or "central america" or "latin america" or "south and central america" or "south america" or "asia, central" or "central asia" or "asia, northern" or "north asia" or "northern asia" or "asia, southeastern" or "southeastern asia" or "south eastern asia" or "southeast asia" or "south east asia" or "asia, western" or "western asia" or "europe, eastern" or "east europe" or "eastern europe" ) OR AB ( mali or malta or micronesia or "federated states of micronesia" or kiribati or "marshall islands" or nauru or "northern mariana islands" or palau or tuvalu or mauritania or mauritius or mexico or moldova or moldovian or mongolia or montenegro or morocco or ifni or mozambique or "portuguese east africa" or myanmar or burma or namibia or nepal or "netherlands antilles" or nicaragua or niger or nigeria or oman or muscat or pakistan or panama or "papua new guinea" or "new guinea" or paraguay or peru or philippines or philipines or phillipines or phillippines or poland or "polish people's republic" or portugal or "portuguese republic" or "puerto rico" or romania or russia or "russian federation" or ussr or "soviet union" or "union of soviet socialist republics" or rwanda or ruanda or samoa or "pacific islands" or polynesia or "samoan islands" or "navigator island" or "navigator islands" or "sao tome and principe" or "saudi arabia" or senegal or serbia or seychelles or "sierra leone" or slovakia or "slovak republic" or slovenia or melanesia or "solomon island" or "solomon islands" or "norfolk island" or "norfolk islands" or somalia or "south africa" or "south sudan" or "sri lanka" or ceylon or "saint kitts and nevis" or "st. kitts and nevis" or "saint lucia" or "st. lucia" or "saint Vincent and the grenadines" or "saint vincent" or "st. vincent" or grenadines or sudan or suriname or surinam or "dutch guiana" or "netherlands guiana" or syria or "syrian arab republic" or tajikistan or tadjikistan or tadzhikistan or tadzhik or tanzania or tanganyika or thailand or siam or "timor leste" or "east timor" or togo or "togolese republic" or tonga or "Trinidad and tobago" or trinidad or tobago or tunisia or turkey or turkmenistan or turkmen or uganda or ukraine or uruguay or uzbekistan or uzbek or vanuatu or "new hebrides" or venezuela or vietnam or "viet nam" or "middle east" or "west bank" or gaza or palestine or yemen or yugoslavia or zambia or zimbabwe or "northern rhodesia" or "global south" or "africa south of the sahara" or "sub-saharan africa" or "subsaharan africa" or "africa, central" or "central africa" or "africa, northern" or "north africa" or "northern africa" or magreb or maghrib or sahara or "africa, southern" or "southern africa" or "africa, eastern" or "east africa" or "eastern africa" or "africa, western" or "west africa" or "western africa" or "west indies" or caribbean or "central america" or "latin america" or "south and central america" or "south america" or "asia, central" or "central asia" or "asia, northern" or "north asia" or "northern asia" or "asia, southeastern" or "southeastern asia" or "south eastern asia" or "southeast asia" or "south east asia" or "asia, western" or "western asia" or "europe, eastern" or "east europe" or "eastern europe" ) | 148,518 |
| S40 | TI ( afghanistan or albania or algeria or "american samoa" or angola or antigua or barbuda or argentina or armenia or armenian or aruba or azerbaijan or bahrain or bangladesh or barbados or belarus or byelarus or belorussia or byelorussian or belize or "british honduras" or benin or dahomey or bhutan or bolivia or "bosnia herzegovina" or bosnia or herzegovina or botswana or bechuanaland or brazil or brasil or bulgaria or "burkina faso" or "burkina fasso" or "upper volta" or burundi or urundi or "cabo verde" or "cape verde" or cambodia or kampuchea or "khmer republic" or cameroon or cameron or cameroun or "central african republic" or "ubangi shari" or chad or chile or china or colombia or comoros or "comoro islands" or "iles comores" or mayotte or "democratic republic of the congo" or "democratic republic congo" or congo or zaire or "costa rica" or "cote d’ivoire" or "cote d’ ivoire" or "cote divoire" or "cote d ivoire" or "ivory coast" or croatia or cuba or cyprus or "czech republic" or czechoslovakia or djibouti or "french somaliland" or dominica or "dominican republic" or ecuador or egypt or "united arab republic" or "el salvador" or "equatorial guinea" or "spanish guinea" or eritrea or estonia or eswatini or swaziland or ethiopia or fiji or gabon or "gabonese republic" or gambia or "georgia (republic)" or georgian or ghana or "gold coast" or gibraltar or greece or grenada or guam or guatemala or guinea or "guinea bissau" or guyana or "british guiana" or haiti or hispaniola or honduras or hungary or india or indonesia or timor or iran or iraq or "isle of man" or jamaica or jordan or kazakhstan or kazakh or kenya or "democratic people’s republic of korea" or "republic of korea" or "north korea" or "south korea" or korea or kosovo or kyrgyzstan or kirghizia or kirgizstan or "kyrgyz republic" or kirghiz or laos or "lao pdr" or "lao people's democratic republic" or latvia or lebanon or lebanese republic or lesotho or basutoland or liberia or libya or "libyan arab jamahiriya" or lithuania or macau or macao or republic of "north macedonia" or macedonia or madagascar or "malagasy republic" or malawi or nyasaland or malaysia or "malay federation" or "malaya federation" or maldives or "indian ocean islands" or "indian ocean" ) OR AB ( afghanistan or albania or algeria or "american samoa" or angola or antigua or barbuda or argentina or armenia or armenian or aruba or azerbaijan or bahrain or bangladesh or barbados or belarus or byelarus or belorussia or byelorussian or belize or "british honduras" or benin or dahomey or bhutan or bolivia or "bosnia herzegovina" or bosnia or herzegovina or botswana or bechuanaland or brazil or brasil or bulgaria or "burkina faso" or "burkina fasso" or "upper volta" or burundi or urundi or "cabo verde" or "cape verde" or cambodia or kampuchea or "khmer republic" or cameroon or cameron or cameroun or "central african republic" or "ubangi shari" or chad or chile or china or colombia or comoros or "comoro islands" or "iles comores" or mayotte or "democratic republic of the congo" or "democratic republic congo" or congo or zaire or "costa rica" or "cote d’ivoire" or "cote d’ ivoire" or "cote divoire" or "cote d ivoire" or "ivory coast" or croatia or cuba or cyprus or "czech republic" or czechoslovakia or djibouti or "french somaliland" or dominica or "dominican republic" or ecuador or egypt or "united arab republic" or "el salvador" or "equatorial guinea" or "spanish guinea" or eritrea or estonia or eswatini or swaziland or ethiopia or fiji or gabon or "gabonese republic" or gambia or "georgia (republic)" or georgian or ghana or "gold coast" or gibraltar or greece or grenada or guam or guatemala or guinea or "guinea bissau" or guyana or "british guiana" or haiti or hispaniola or honduras or hungary or india or indonesia or timor or iran or iraq or "isle of man" or jamaica or jordan or kazakhstan or kazakh or kenya or "democratic people’s republic of korea" or "republic of korea" or "north korea" or "south korea" or korea or kosovo or kyrgyzstan or kirghizia or kirgizstan or "kyrgyz republic" or kirghiz or laos or "lao pdr" or "lao people's democratic republic" or latvia or lebanon or lebanese republic or lesotho or basutoland or liberia or libya or "libyan arab jamahiriya" or lithuania or macau or macao or republic of "north macedonia" or macedonia or madagascar or "malagasy republic" or malawi or nyasaland or malaysia or "malay federation" or "malaya federation" or maldives or "indian ocean islands" or "indian ocean" ) | 230,968 |
| S39 | S1 OR S38 | 15,244 |
| S38 | S16 AND S37 | 15,243 |
| S37 | S17 OR S18 OR S19 OR S20 OR S21 OR S22 OR S23 OR S24 OR S25 OR S26 OR S27 OR S28 OR S29 OR S30 OR S31 OR S32 OR S33 OR S34 OR S35 OR S36 | 354,620 |
| S36 | (MH "Product Surveillance+/SN") | 1 |
| S35 | (MH "Maternal Health Services+/SN") | 1,245 |
| S34 | TI pharmacovigilan* or (pharmaco W0 vigilan*) | 846 |
| S33 | (MH "Product Surveillance+") | 2,417 |
| S32 | TI "data system" or "data systems" or "information system" or "information systems" | 4,466 |
| S31 | (MH "Health Information Systems") | 3,566 |
| S30 | TI (decision* AND support* AND clinical*) | 1,865 |
| S29 | (MH "Decision Support Systems, Clinical") | 6,139 |
| S28 | TI surveillance* | 15,975 |
| S27 | (MH "Population Surveillance+") | 11,277 |
| S26 | TI survey* | 71,609 |
| S25 | (MH "Surveys") | 157,567 |
| S24 | TI registry or registries or eregistr* or "e-registry" or "e-registries" | 13,946 |
| S23 | TI (preliminary or "pilot project" or "pilot projects") AND data | 682 |
| S22 | TI "focus group" or "focus groups" | 2,957 |
| S21 | (MH "Focus Groups") | 48,414 |
| S20 | TI databas* or "data base" or "data bases" or databank* or "data bank" or "data banks" or dataset* or "data set" or "data sets" | 16,019 |
| S19 | TI data AND (accumulat* or accura* or assembl* or captur* or collect* or compil* or coordinat* or co-ordinat* or gather* or hub or hubs) | 4,425 |
| S18 | (MH "Data Curation") | 184 |
| S17 | (MH "Data Collection") | 49,942 |
| S16 | S2 OR S3 OR S4 OR S5 OR S6 OR S7 OR S8 OR S9 OR S10 OR S11 OR S12 OR S13 OR S14 OR S15 | 290,175 |
| S15 | TI maternal AND (fetal or fetus or foetal or foetus) | 4,004 |
| S14 | TI (maternal* or maternity) AND (care or health* or service or services) | 9,718 |
| S13 | (MH "Maternal Health Services") | 11,199 |
| S12 | TI (maternal* or maternity) AND (contact* or expos*) | 1,028 |
| S11 | (MH "Maternal Exposure") | 2,588 |
| S10 | TI perinatal* or "peri-natal" or "peri-natally" or peripartum or "peri-partum" | 12,858 |
| S9 | (MH "Perinatal Care") | 5,019 |
| S8 | TI prenatal* or antenatal* or "ante-natal" or "ante-natally" or antepartum or "ante-partum" | 20,877 |
| S7 | (MH "Prenatal Care") | 19,422 |
| S6 | TI pregnan* | 80,123 |
| S5 | (MH "Pregnancy Trimesters+") | 13,521 |
| S4 | (MH "Expectant Mothers") | 11,382 |
| S3 | (MH "Pregnancy Complications+") | 106,825 |
| S2 | (MH "Pregnancy") OR (MH "Pregnancy, Multiple+") OR (MH "Pregnancy, Unplanned") OR (MH "Pregnancy, Unwanted") OR (MH "Pregnancy, High Risk") OR (MH "Pregnancy, Prolonged") | 227,240 |
| S1 | TI ( "pregnancy exposure database" OR "pregnancy exposure databases" OR "pregnancy exposure data base" OR "pregnancy exposure data bases" OR "pregnancy exposure registry" OR "pregnancy exposure registries" ) OR AB ( "pregnancy exposure database" OR "pregnancy exposure databases" OR "pregnancy exposure data base" OR "pregnancy exposure data bases" OR "pregnancy exposure registry" OR "pregnancy exposure registries" ) | 7 |

Global Index Medicus

tw:((tw:(pregnan* exposure* database*)) OR (tw:(pregnan* exposure* "data base")) OR (tw:(pregnan* exposure* "data bases")) OR (tw:(pregnan* exposure* databank*)) OR (tw:(pregnan* exposure* "data bank")) OR (tw:(pregnan* exposure* "data banks")) OR (tw:(pregnan* exposure* register)) OR (tw:(pregnan* exposure* registries)) OR (tw:(pregnan* exposure* registry)) OR (tw:(pregnan* exposure* registries))) – *108 records*

tw:((ti:((pregnan* OR prenatal* OR antenatal* OR "ante-natal" OR "ante-natally" OR antepartum OR "ante-partum" OR perinatal* OR "peri-natal" OR "peri-natally" OR peripartum OR "peri-partum" OR maternal*) AND (data OR database* OR databank* OR register OR registers OR registry OR registries OR survey* OR surveillance OR pharmacovigilan* OR "pharmaco-vigilance") AND (safe OR safety OR "side effect" of "side effects" OR "undesirable effect" OR "undesirable effects" OR "treatment emergent" OR tolerability OR toxicity OR adrs OR aefi)))) – *361 records*

tw:(ti:((pregnan* OR prenatal* OR antenatal* OR "ante-natal" OR "ante-natally" OR antepartum OR "ante-partum" OR perinatal* OR "peri-natal" OR "peri-natally" OR peripartum OR "peri-partum" OR maternal*) AND (data OR database* OR databank* OR register OR registers OR registry OR registries OR survey* OR surveillance OR pharmacovigilan* OR "pharmaco-vigilance") AND (MNCH or "maternal health" or "child health" or "infant health" or "birth defect" or "birth defects" or "birth outcome" or "birth outcomes" or "pregnancy outcome" or "pregnancy outcomes" or "neonatal outcome" or "neonatal outcomes"))) – *50 records*

tw:(ti:((pregnan* OR prenatal* OR antenatal* OR "ante-natal" OR "ante-natally" OR antepartum OR "ante-partum" OR perinatal* OR "peri-natal" OR "peri-natally" OR peripartum OR "peri-partum" OR maternal*) AND (data OR database* OR databank* OR register OR registers OR registry OR registries OR survey* OR surveillance OR pharmacovigilan* OR "pharmaco-vigilance") AND (vaccine or vaccines or vaccination* or immunization* or immunization* or “maternally-acquired”))) – *0 records*

tw:((ti:((pregnan* OR prenatal* OR antenatal* OR "ante-natal" OR "ante-natally" OR antepartum OR "ante-partum" OR perinatal* OR "peri-natal" OR "peri-natally" OR peripartum OR "peri-partum" OR maternal*) AND (data OR database* OR databank* OR register OR registers OR registry OR registries OR survey* OR surveillance OR pharmacovigilan* OR "pharmaco-vigilance") AND (drug OR drugs OR medicine OR medicines OR medication* OR pharmaceutical* OR "pharma-ceutical" OR "pharma-ceuticals" OR vaccin* OR immun* OR antimalarial* OR "anti-malarial" OR "anti-malarials" OR antiviral* OR "anti-viral" OR "anti-virals" OR antiretroviral* OR "anti-retroviral" OR "anti-retrovirals" OR "Anti-HIV" OR "Anti-AIDS")))) – *10 records*

tw:((ti:((pregnan* OR prenatal* OR antenatal* OR "ante-natal" OR "ante-natally" OR antepartum OR "ante-partum" OR perinatal* OR "peri-natal" OR "peri-natally" OR peripartum OR "peri-partum" OR maternal*) AND (data OR database* OR databank* OR register OR registers OR registry OR registries OR survey* OR surveillance OR pharmacovigilan* OR "pharmaco-vigilance") AND (fetal* OR foetal* OR fetus* OR foetus* OR neonat* OR newborn* OR infant OR infants OR infanc* OR child*) ))) – *125 records*

tw:((ti:((pregnan* OR prenatal* OR antenatal* OR "ante-natal" OR "ante-natally" OR antepartum OR "ante-partum" OR perinatal* OR "peri-natal" OR "peri-natally" OR peripartum OR "peri-partum" OR maternal*) AND (data OR database* OR databank* OR register OR registers OR registry OR registries OR survey* OR surveillance OR pharmacovigilan* OR "pharmaco-vigilance"))) AND (tw:(drug OR drugs OR medicine OR medicines OR medication* OR pharmaceutical* OR "pharma-ceutical" OR "pharma-ceuticals" OR vaccin* OR immunisation* OR immunization* OR antimalarial* OR "anti-malarial" OR "anti-malarials" OR antiviral* OR "anti-viral" OR "anti-virals" OR antiretroviral* OR "anti-retroviral" OR "anti-retrovirals" OR "Anti-HIV" OR "Anti-AIDS"))) – *71 records*

**TOTAL: 725 records**

Google Scholar

"pregnant|pregnancy|prenatal"+"registry|registries|surveillance|pharmacovigilance" + "expose|exposed|exposes|exposure|exposures"+[names of LMICs]

Data extraction form

**PART 1: STUDY IDENTIFICATION / INCLUSION STATUS**

Resource title:

Author(s):

Year of publication:

Resource type:

1. 1 - Peer reviewed journal

2. 2 - Grey literature

3. 3 - Registry

4. 4 - Website

5. 5 - Key informant survey / interview

6. 6 - Other

Database resource pulled from:

Final status:

1. 1 - included

2. 2 - excluded

3. 3 - incomplete/unclear

If excluded, explain reasoning:

**PART 2: REGISTRY INFORMATION OR OTHER RESOURCE**

Name of registry or other resource:

Primary goals/aims:

Funding source:

Years of operation:

Current status

1. Active

2. Inactive

3. On hold

Country(ies) where located:

Country representativeness:

1. Multi-national

2. National

3. State/Provincial

4. District

5. Sub-district

6. Community

7. Health clinic

8. Hospital

9. Other

Drug/vaccine exposure name and type:

Duration of follow up:

Current sample size:

1. <500 participants

2. 500 - 1,999 participants

3. 2,000 - 5,000 participants

4. 5,001 - 10,000 participants

5. 10,001 - 20,000 participants

6. >20,000 participants

Data collection:

1. Administrative databases

2. Medical databases

3. Registries

4. Research study

5. Program implementation

6. Other

Methodology:

Terminology and data system used:

**PART 3: CHARACTERISTICS OF INCLUDED POPULATION**

Age ranges included:

Target population:

1. Pregnant women

2. Non-pregnant women / women of reproductive age

3. Children

4. Infants (<28 days old)

5. General population

6. Other

Gestational age

1. First trimester

2. Second trimester

3. Third trimester

Underlying medical condition(s):

Maternal outcome(s) recorded:

1. Thrombosis and/or thrombocytopenia syndrome

2. Antenatal hospitalization (not including delivery)

3. Disability

4. Gestational diabetes

5. Gestational hypertension

6. Premature rupture of membranes

7. Preterm labor

8. Preeclampsia / Eclampsia

9. Post-partum hemorrhage

10. Antenatal hemorrhage

11. Corporeal infection

12. Spontaneous abortion / miscarriage / pregnancy loss (example: prior to 20 weeks gestation)

13. Maternal death (example: within 42 days of termination of pregnancy)

14. Late maternal death (example: >42 days of termination of pregnancy)

15. Other

Maternal death cause:

1. Direct cause

2. Indirect cause

Neonatal outcome(s) recorded:

1. Preterm birth

2. Small size for gestational age / restricted fetal growth

3. Still birth (death after 28 weeks of pregnancy but before birth)

4. Live birth

5. Congenital anomaly / birth defect

6. Death

7. Other

Neonatal death timeframe

1. Early neonatal (0-7 days)

2. Late neonatal (8-28 days)

3. Post neonatal (29 days - 1 year)

Which congenital anomaly does this monitor (if any)?

Infant/child outcome(s) recorded:

1. Infections

2. Respiratory illness

3. Developmental outcomes

4. Other

What is the duration of follow up?

**PART 4: KEY FINDINGS**

Populate as best you can if information is provided; answer N/A if it is not relevant or N/P if it is not provided.

Strengths of the registry or other resource:

Weaknesses / gaps of registry or other resource:

Challenges of registry or other resource in its specific context:

Does this resource have the possibility to add new interventions?

1. Yes

2. No

3. Maybe

4. Unknown Why?

Can this resource be combined with other systems?

1. Yes

2. No

3. Maybe

4. Unknown Why?

Any upcoming changes to the resource:

Additional comments

**PART 1: STUDY IDENTIFICATION / INCLUSION STATUS**

Resource title:

Author(s):

Year of publication:

Resource type:

1. 1 - Peer reviewed journal

2. 2 - Grey literature

3. 3 - Registry

4. 4 - Website

5. 5 - Key informant survey / interview

6. 6 - Other

Database resource pulled from:

Final status:

1. 1 - included

2. 2 - excluded

3. 3 - incomplete/unclear

If excluded, explain reasoning:

**PART 2: REGISTRY INFORMATION OR OTHER RESOURCE**

Name of registry or other resource:

Primary goals/aims:

Funding source:

Years of operation:

Current status

1. Active

2. Inactive

3. On hold

Country(ies) where located:

Country representativeness:

1. Multi-national

2. National

3. State/Provincial

4. District

5. Sub-district

6. Community

7. Health clinic

8. Hospital

9. Other

Drug/vaccine exposure name and type:

Duration of follow up:

Current sample size:

1. <500 participants

2. 500 - 1,999 participants

3. 2,000 - 5,000 participants

4. 5,001 - 10,000 participants

5. 10,001 - 20,000 participants

6. >20,000 participants

Data collection:

1. Administrative databases

2. Medical databases

3. Registries

4. Research study

5. Program implementation

6. Other

Methodology:

Terminology and data system used:

**PART 3: CHARACTERISTICS OF INCLUDED POPULATION**

Age ranges included:

Target population:

1. Pregnant women

2. Non-pregnant women / women of reproductive age

3. Children

4. Infants (<28 days old)

5. General population

6. Other

Gestational age

1. First trimester

2. Second trimester

3. Third trimester

Underlying medical condition(s):

Maternal outcome(s) recorded:

1. Thrombosis and/or thrombocytopenia syndrome

2. Antenatal hospitalization (not including delivery)

3. Disability

4. Gestational diabetes

5. Gestational hypertension

6. Premature rupture of membranes

7. Preterm labor

8. Preeclampsia / Eclampsia

9. Post-partum hemorrhage

10. Antenatal hemorrhage

11. Corporeal infection

12. Spontaneous abortion / miscarriage / pregnancy loss (example: prior to 20 weeks gestation)

13. Maternal death (example: within 42 days of termination of pregnancy)

14. Late maternal death (example: >42 days of termination of pregnancy)

15. Other

Maternal death cause:

1. Direct cause

2. Indirect cause

Neonatal outcome(s) recorded:

1. Preterm birth

2. Small size for gestational age / restricted fetal growth

3. Still birth (death after 28 weeks of pregnancy but before birth)

4. Live birth

5. Congenital anomaly / birth defect

6. Death

7. Other

Neonatal death timeframe

1. Early neonatal (0-7 days)

2. Late neonatal (8-28 days)

3. Post neonatal (29 days - 1 year)

Which congenital anomaly does this monitor (if any)?

Infant/child outcome(s) recorded:

1. Infections

2. Respiratory illness

3. Developmental outcomes

4. Other

What is the duration of follow up?

**PART 4: KEY FINDINGS**

Populate as best you can if information is provided; answer N/A if it is not relevant or N/P if it is not provided.

Strengths of the registry or other resource:

Weaknesses / gaps of registry or other resource:

Challenges of registry or other resource in its specific context:

Does this resource have the possibility to add new interventions?

1. Yes

2. No

3. Maybe

4. Unknown Why?

Can this resource be combined with other systems?

1. Yes

2. No

3. Maybe

4. Unknown Why?

Any upcoming changes to the resource:

Additional comments

Key informant survey and interview

Pregnancy Exposure Data and Resources Stakeholder Survey

We are conducting a landscape analysis in collaboration with WHO to identify current and recent resources, including pregnancy exposure and surveillance registries, databases, cohort surveys, and routinely collected data, that record exposure to medicines and vaccines during pregnancy and maternal and perinatal outcomes in low- and middle-income countries (LMICs). We are asking for your help in identifying examples of these resources. We may follow up with you to discuss the appropriateness or fit for purpose of the resource you identify. Our goal is to understand what is currently available in LMICs and make connections for future evaluation of maternal use of medicines and vaccines in the product pipeline.

You have been identified as someone who is knowledgeable about or involved with these resources in LMICs. Please complete the following form for each resource you know of. We will ask for your name and contact information so that we may follow up with you for further information, if necessary. All of the personal information you provide will be kept confidential. When we report our findings, if we need to mention something you have said or information you have provided, we will refer to you by a unique study ID to keep your identity confidential. By submitting the form, you are agreeing to participate and allow us to use the information you have provided.

Please fill out the following questions to the best of your knowledge. If there are any specific points that are not included as options in the dropdown menus that are relevant to the resource, please type in the answer and hit “enter”.

**Participant details**

Please note we may contact you to follow up about the resource you describe if we have any questions.

Name*: ____________________________________________________________________________

Email*: ____________________________________________________________________________

Organization*: ______________________________________________________________________

Job Title: ___________________________________________________________________________

**Resource details**

Below we will be asking you to fill in information about any resources you are familiar with as outlined above. If you know of multiple resources that should be brought to our attention, please fill out a separate survey for each resource. As a reminder, resources can include pregnancy exposure and surveillance registries, databases, cohort surveys, and routinely collected data, that record exposure to medicines and vaccines during pregnancy and maternal and perinatal outcomes in low- and middle-income countries (LMICs)

Resource or Project Name: ___________________________________________________________

Please provide a link to the resource if available: __________________________________________

What location(s) does the resource cover (country/countries or region(s))? ____________________

Who oversees or maintains the resource? Please provide the name of the organization/s or specific person(s) and their contact information if available.

Name of organization: _______________________________________________________________

Primary contact name: _______________________________________________________________

Primary contact email: _______________________________________________________________

How is data collected? Select all that apply.

- Administrative databases
- Medical databases
- Registries
- Research study
- Program implementation
- Other

If you selected “other”, please specify: ______________________________________________

Any additional details you would like to provide? :_______________________________

The data in this resource are captured at a:

- Multi-national
- National
- State / Provincial
- District
- Sub-district
- Community
- Health clinic
- Hospital
- Other

If you selected “other”, please specify: ______________________________________________

How are these data collected?

- Retrospectively
- Prospectively

How many individuals are enrolled in this resource (total)?

- <500 participants
- 500 – 2,000 participants
- 2,000 – 5,000 participants
- 5,001 – 10,000 participants
- 10,001 – 20,000 participants
- >20,000 participants

What population(s) are the target for this resource. Select all that apply.

- Pregnant women
- Non-pregnant women / women of reproductive age
- Breastfeeding women
- Children
- Infants (<28 days old)
- General population

What intervention(s) does this resource include? Select all that apply.

- Vaccine/Immunization
  - COVID-19
  - Influenza
  - Meningococcus
  - Tetanus toxoid
  - Pertussis
  - Other

If you selected “other”, please specify: ___________________________

- Medicines/Drugs/Biologics
  - Antimalarials
  - Antiretrovirals/HIV/AIDS
  - Medicines related to mental health
  - Medicines related to autoimmune diseases
  - Medicines related to cancer
  - Medicines related to diabetes
  - Medicines related to epilepsy
  - Other

If you selected “other”, please specify: ______________________________________________

What outcome(s) is/are recorded while under observation in this resource? Please select all that apply.

- Maternal outcomes
  - Thrombosis and/or thrombocytopenia syndrome
  - Antenatal hospitalization not including delivery
  - Disability
  - Gestational diabetes
  - Gestational hypertension
  - Premature rupture of membranes
  - Preterm labor
  - Preeclampsia / eclampsia
  - Post-partum hemorrhage
  - Antenatal hemorrhage
  - Corporeal infection
  - Spontaneous abortion / miscarriage / pregnancy loss (example: prior to 20 weeks gestation)
  - Maternal death (example: within 42 days of termination of pregnancy)
  - Late maternal death (example: >42 days – 1 year after termination of pregnancy)
  - Other

If you selected “maternal death” or “late maternal death”, please specify the cause of death:

- Direct
- Indirect

If you selected “other”, please specify: ______________________________________________

- Neonatal outcomes
  - Preterm birth
  - Small size for gestational age / restricted fetal growth
  - Stillbirth (death after 28 weeks of pregnancy but before birth)
  - Live birth
  - Congenital anomalies / birth defects
  - Death
  - Other

If “congenital anomalies / birth defects” is selected, please specify: ________________________

If neonatal “death” was selected, please specify the timeframe:

- Early neonatal (0-7 days)
- Late neonatal (8-28 days)
- Post neonatal (29 days – 1 year)

If you selected “other”, please specify: ______________________________________________

- Infant/child outcomes
  - Neonatal infections
  - Respiratory illness
  - Developmental outcomes
    - Specify (motor, cognitive, neurologic, autism, etc.): _____________________
  - Other

If you selected “other”, please specify: ______________________________________________

What is the duration of follow up? _______________________________________________

Resource start date: _________________________________________________________________

Resource end date (if applicable): _____________________________________________________

What is the current status of the resource?

- Open
- Closed

Who has access to this resource and its data? ____________________________________________

Do you participate in the running of this resource?

- Yes
- No

Do you contribute data to this resource?

- Yes
- No
- Not applicable

Are you a user of this resource?

- Yes
- No

Related publications/links: ___________________________________________________________

Anything else you’d like to share? _____________________________________________________

*File upload.* If available, please upload any relevant documents here from the resource, including templates, data collection forms, data dictionaries, data structures, publications, etc. These documents will help us understand the breadth of information that is captured in this resource.

PERLA Key Informant Interview

**Consent script**

Hello, my name is _____________ and I work at PATH, an international NGO working in health. We are conducting a landscape analysis in collaboration with WHO to identify available resources, including pregnancy exposure and surveillance registries, databases, surveys, and routinely collected data, that record exposure to medical products during pregnancy and maternal and perinatal outcomes in low- and middle-income countries (LMICs). You either filled out a survey about one or more resources that fit this description, or have been identified as someone who may have more information on these types of resources and may be interested in telling us more. This interview will take between 15-30 minutes and we will ask for your feedback and impressions on the resource(s) you’ve identified. Please keep in mind there are no right or wrong answers, and we are interested in your understanding of the resource as someone who maintains or interacts with these types of resources. We may record this session to help us later with our report. No video footage will be recorded, only audio. Participation is voluntary and if you would like to stop or not answer a question, you may do so at any time. Your name or identity will not be associated with the feedback you provide. Results may be compiled into a report, peer reviewed manuscript, or other communications materials that will be made publicly available. If we make reference to something you have said in our report, you would be referred to as “Participant #1, or #2, etc.” Please confirm your interest in participating in this session?

- Yes
- No

Thank you for agreeing to participate in this interview. Let’s get started. **(I will start the recording now.)**

This is an interview with participant _________.

**Interviewee information**

*For interviewer only: Please fill this section out before the interview and do not speak any identifying information over the recording. If any sections of their survey have been left blank, please include those as follow up questions.*

Unique ID. Please reference the sheet if they have already filled out a survey and use their unique ID here. If they did not fill out a survey, assign them a new unique ID. ____________________________

Name: _____________________________________________________________________________

Email: _____________________________________________________________________________

Organization: ________________________________________________________________________

Title: _______________________________________________________________________________

Did they complete a survey form?

- Yes
- No

**Resource information**

*For interviewers only: If “No” is selected under the previous question, all the questions from the survey will show up. If “Yes” is selected, the survey questions that have been answered will be skipped and go straight into the following questions.*

We are interested in understanding whether this resource is well equipped or well suited for its designed role or purpose. This could include the possibility of the resource to be combined with others for broader safety surveillance purposes and to understand its impact on supporting maternal health generally.

1. Can you tell me about the overall goals of the resource you have described to us?

*Probe: target population, intervention monitored, outcomes monitored, enrollment size, etc.*

1. How have findings from this resource been used? In particular, have they been useful decision-making?

*Probe: in public health? In regulation? For clinicians? For other healthcare bodies?*

1. What are the advantages of this resource?

*Probe: Comprehensiveness (outcomes or breadth of resources), coverage, timeliness, usefulness, accuracy, completeness .*

1. What are the most important gaps or needs of this resource?

*Probe on gaps: Comprehensiveness (outcomes or breadth of resources), coverage, timeliness, usefulness, accuracy, completeness.*

1. What are the challenges this resource faces in the current context?

*Probe on challenges: Cost, time requirements to maintain the resource, software limitations, etc.*

1. Could data from this resource be able to be combined with other health surveillance resources that are used in this region?

*Probe: Why/why not? Are there any challenges you foresee?*

*Probe: How could these systems link?*

1. Are you aware of any changes that will be made to this resource in the foreseeable future?
2. Is there anyone else you know who might have useful information about resources like the ones we discussed today?

That was my last question. Before we finish, do you have any questions or additional comments regarding the topics discussed during this interview?

Thank you for your time today. Your input is greatly appreciated.

**Table S1. Key characteristics of identified pregnancy exposure registries and related resources**

| **Resource Name** | **Countries** | **Design and Eligibility** | **Maternal Outcomes** | **Neonatal and Infant/child outcomes** | **References** |
| --- | --- | --- | --- | --- | --- |
| **Pregnancy Exposure Registries** | | | | | |
| **CHERISH (Children HIV Exposed Uninfected Research to Inform Survival and Health)** | South Africa | Product focus: antiretrovirals  Prospective  Pregnant women with known HIV status at 24–36 weeks estimated gestational age  Follow-up of children to 3-5 years | Not specified | Infant and under 3-year survival; infant and under 3-year all-cause and infectious-cause hospitalization; growth and neurodevelopmental outcomes at 3–5 years of age | [1] |
| **C-VIPER (COVID-19 Vaccines International Pregnancy Exposure Registry) and PIPER (Pregistry International Pregnancy Exposure Registry)** | Global, based in the United States | Product focus: COVID-19 vaccines  Pregnant women exposed (C-VIPER) and not exposed (PIPER) to COVID-19 vaccines  Comparison group  Infants followed through 1 year of age | Spontaneous abortion, antenatal bleeding, gestational diabetes, gestational hypertension, intrauterine growth restriction, postpartum hemorrhage, fetal distress, uterine rupture, placenta previa, chorioamnionitis, Caesarean delivery, or COVID-19 | Major congenital malformations, low birth weight, neonatal death, neonatal encephalopathy, neonatal infections, neonatal acute kidney injury, preterm birth, respiratory distress in the newborn, small for gestational age, stillbirth, or COVID-19; infant weight, length, developmental milestones through 1 year of age | [2–5] |
| **COVID-PR (COVID-19 International Drug Pregnancy Registry)** | Global, based in the United States | Product focus: COVID-19 drugs (antivirals and monoclonal antibodies)  Pregnant women exposed to COVID-19 drugs  Comparison groups:  1. Pregnant women treated with another therapy for COVID-19  2. Pregnant women hospitalized but not treated for COVID-19  Infants followed through 1 year of age | Spontaneous abortion, intrauterine growth restriction, gestational diabetes, gestational hypertension, postpartum hemorrhage, Caesarean delivery | Major congenital malformations, low birth weight, small for gestational age, neonatal infections, stillbirth, neonatal death, preterm birth; infant weight, length, developmental milestones through 1 year of age. | [5–7] |
| **MANGO (Measuring Adverse Pregnancy and Newborn Congenital Outcomes)** | Kenya (western) | Product focus: antiretrovirals  Prospective and retrospective  Comparison group  Pregnant women with known HIV status, 1:1 ratio of HIV positive to negative | Live birth, stillbirth, miscarriage, termination of pregnancy, ectopic pregnancy, molar pregnancy  Also includes pre-term delivery (<37 weeks gestational age) or very pre-term delivery (<32 weeks gestational age) | Congenital abnormalities on newborn surface exam (e.g., extra digit, hydrocephalus, skull defects, eyes, face, mouth/lip/palate, chest, abdomen, anus, limbs, spine (including neural tube defects), hips, genitalia, skin, etc.)  Also includes low birth weight, small for gestational age (<10th percentile), or very small for gestational age (<3rd percentile) | [8,9] |
| **MiMba (Malaria in Mothers and Babies) Pregnancy Registry** | Kenya, Burkina Faso | Product focus: antimalarials  Prospective  Comparison group  Pregnant women exposed/unexposed to antimalarials during pregnancy  Mothers followed through delivery  Infants followed through two years of age | Miscarriage, stillbirth, maternal mortality | Major congenital anomalies, neonatal mortality, low birthweight, prematurity | [10,11] |
| **REPRESENT (Xiamen Registry of Pregnant Women and Offspring)** | China (Xiamen) | Product focus: none specified  Retrospective  Mothers followed to 42 days after delivery  Infants followed through childhood | Preeclampsia/eclampsia, gestational diabetes, uterine rupture, postpartum hemorrhage, stillbirth, maternal death | Congenital anomalies, preterm birth, low birth weight, neonatal death | [12–14] |
| **UBOMI BUHLE (Understanding Birth Outcomes from Mothers and Infants, Building Healthcare by Linking Exposures) Pregnancy Exposure Registry** | South Africa (3 provinces)  Incorporating the Western Cape Pregnancy Exposure Registry | Product focus: antiretrovirals  Prospective  All pregnant women attending antenatal care  Mothers followed through delivery  Infants followed through neonatal period up to discharge. | Gestational diabetes, gestational hypertension, maternal death, post-partum hemorrhage, preeclampsia / eclampsia, preterm labor, spontaneous abortion / miscarriage / pregnancy loss | Congenital anomalies on newborn surface exam, neonatal death, preterm birth, small size for gestational age /restricted fetal growth, stillbirth | [15–19] |
| **Health and Demographic Surveillance Systems and other Observational cohorts** | | | | | |
| **CHAMPS (Child Health and Mortality Prevention Surveillance Network) pregnancy surveillance** | Global, based in the United States | Product focus: none specified  Prospective and retrospective  All pregnancies in catchment area  Follow-up 42 days after delivery | Maternal death, post-partum hemorrhage, pre-eclampsia and eclampsia, obstructed labor, acute infections (e.g., chorioamnionitis or sepsis), Cesarean section, miscarriage | Neonatal death, neonatal resuscitation stillbirth, low/very low birthweight | Personal communication |
| **IeDEA (International Epidemiology Databases to Evaluate AIDS)** | Multinational | Product focus: antiretrovirals  Various study designs  All populations living with or at risk for HIV  Variable follow-up | Variable | Variable | [20–28,8,29] |
| **INDEPTH** | Multinational | Product focus: none specified  Prospective  General population  Variable follow-up | Maternal death, gestational hypertension, fetal distress, postpartum hemorrhage, spontaneous abortion, antenatal bleeding | Neonatal death, congenital anomalies, neonatal infections, preterm birth, stillbirth, low birthweight, small for gestational age | [30–48,44] |
| **Maternal Newborn Health Registry** | Multinational | Product focus: none specified  Prospective  Pregnant women  Mothers followed 42 days post-partum | Maternal death, pre-eclampsia/eclampsia, gestational hypertension, fetal distress, ectopic pregnancy, postpartum hemorrhage, spontaneous abortion, antenatal bleeding, dysfunctional labor, fetal growth retardation, gestational diabetes, endometritis, chorioamnionitis, PPROM | Neonatal death, congenital anomalies, neonatal infections, preterm birth, stillbirth, low birthweight, small for gestational age, respiratory distress, neonatal seizures | [49–67] |
| **PREPARE** | Uganda | Product focus: antimalarials, antiretrovirals, COVID-19 vaccines, tetanus-diphtheria vaccine  Prospective  Pregnant women  Mothers and infants followed for 9 months after delivery | Serious adverse events, medically-attended events, obstetric complications | Serious adverse events, medically attended events, major congenital anomalies, developmental delay | [68] |
| **Shoklo Malaria Research Unit** | Thailand | Product focus: antimalarials  Prospective  Pregnant women  Mothers followed through delivery  Infants followed through neonatal period | Miscarriage (primary) | Major congenital malformations (secondary) | [69–71] |
| **UCLA DRC Research Program** | Democratic Republic of Congo | Product focus: none specified  Prospective and retrospective components  Pregnant women  Infants followed for 28 days after delivery | Preterm birth; stillbirth | Invasive bloodstream infection; neonatal death, congenital microcephaly, low birth weight; small for gestational age | [72,73] |
| **Outcomes-based registries** | | | | | |
| **BBDSFP (Bogota Birth Defects Surveillance and Follow-up Program)** | Colombia | Product focus: none specified  Retrospective  All deliveries  Neonatal period | Maternal illnesses by ICD code | Congenital anomalies | [74–76] |
| **CHAMPS (Child Health and Mortality Prevention Surveillance) stillbirth and neonatal and mortality surveillance** | Multinational | Product focus: none specified  Retrospective  Stillbirths and neonatal deaths  Neonatal period | stillbirth, spontaneous abortion | Neonatal death | [47,77] |
| **CTBC (China Teratology Birth Cohort)** | China | Product focus: antiretrovirals; antimalarials; medicines related to autoimmunity, cancer, diabetes, and epilepsy  Prospective  Comparison group  All deliveries  Followed for 42 days after delivery | stillbirth, spontaneous abortion | Congenital anomalies, preterm birth, post-term birth, low birth weight, macrosomia, small for gestational age, large for gestational age, low Apgar score | [78] |
| **Eswatini Birth Defects Study** | Eswatini | Product focus: antiretrovirals  Retrospective  Comparison group | N/A | Major and minor surface congenital anomalies | [79,80] |
| **Makerere Birth Defects Surveillance Project** | Uganda | Product focus: antiretrovirals; tetanus toxoid vaccine  Retrospective  Comparison group  All deliveries  Neonatal period | Spontaneous abortion, stillbirth | Congenital anomalies; congenital infections | [81–83] |
| **Malawi Birth Defects Surveillance** | Malawi | Product focus: none specified  Retrospective  All deliveries | N/A | Major external congenital anomalies | [84,85] |
| **Tsepamo Study** | Botswana | Product focus: antiretrovirals  Retrospective  Comparison group  All deliveries  Neonatal period | Preterm delivery, stillbirth | Congenital anomalies on surface exam, preterm birth, small for gestational age, or neonatal death | [86–92] |
| **Maternal conditions-based registries** | | | | | |
| **Kerala registry of Epilepsy and Pregnancy (KREP)** | India | Product focus: anti-epileptics  Prospective, enrolled in the preconception period or first trimester  Exposed and unexposed (comparator)  Pregnant women with epilepsy | Seizure frequency. Pregnancy induced hypertension, pre-eclampsia, intra-uterine growth retardation, pre- and post-term delivery, placenta previa, hydramnios, spontaneous and therapeutic abortion, intra-uterine death and stillbirth, neonatal death, postpartum hemorrhage. | Major congenital malformations; chromosomal defects. Pre-term and post-term birth. | [93–100] |
| **REBECGA Brazilian Registry of Pregnancy and Heart Disease** | Brazil | Product focus: cardiac medications  Prospective (longitudinal) and Retrospective (cross-sectional) stages  Pregnant women with heart disease | Cardiovascular complications during delivery and puerperium; obstetric complications of pregnancy, delivery, and puerperium; Hospitalization for treating cardiovascular complications during pregnancy or puerperium; late maternal death up to 12 months after delivery. | Congenital malformations | [101] |
| **Tamil Nadu Pregnancy and Heart Disease Registry (TNPHDR)** | India | Product focus: cardiac medications  Prospective  Pregnant women with cardiac disease | Cardiac: Cardiac death, cardiac arrest, cardiac hospitalization, new or worsening heart failure, new arrhythmia, thromboembolic event, hemorrhage, aortic dissection, endocarditis, acute coronary syndrome, or cardiac intervention during pregnancy and up to 1 week post-delivery  Obstetric: gestational diabetes mellitus, gestational hypertension, pre- eclampsia, eclampsia / HELLP syndrome, preterm labor, premature rupture of membranes, fetal loss. | Premature birth, intra-uterine growth retardation or small-for-gestational-age and congenital heart disease or anomalies | [102] |
| **Manufacturer registries** | | | | | |
| **APR (Antiretroviral Pregnancy Registry)** | Multinational (70-80% US) | Product focus: antiretrovirals (HIV and HBV)  Prospective  No comparison group  Voluntary; pregnant women exposed to antiretrovirals  Mothers followed through delivery  Infants followed through 28 days | Spontaneous abortion, miscarriage, pregnancy loss | Congenital anomalies, death, live birth, preterm birth, small size for gestational age / restricted fetal growth, stillbirth | [20,103–106,86,107] |
| **Bayer pharmacovigilance (PV) database** | International, with select middle-income countries (Russia, South Africa) | Product focus: Interferon beta-1b (Betaferon(R), Betaseron(R), Extavia(R))  Prospective  Retrospective (for comparison)  Cases reported to Bayer’s global PV database and entered prospectively (before outcome is known) | Spontaneous abortions, stillbirth/fetal death, ectopic pregnancies | Congenital anomalies | [108] |
| **EURAP: International Registry of Antiepileptic Drugs and Pregnancy** | Mainly high-income countries, but also India, Philippines | Product focus: anti-epileptics  Prospective (cases that are enrolled after 16wks, after prenatal dx, or after birth are reported descriptively)  Women exposed to antiepileptic drugs at the time of conception | Seizure frequency | Stillbirths, elective terminations due to fetal abnormalities  Major congenital malformations up to 12 months after birth | [109–114] |
| **GlaxoSmithKline (GSK) Pregnancy Registries** | Global | Product focus: Vaccines (influenza, tetanus, pertussis, varicella, measles-mumps-rubella [MMR])  Antimalarials; antiretrovirals; medications related to autoimmune diseases, epilepsy, and asthma  Prospective  Retrospective  Comparison group  Pregnant women | Varies by product; includes spontaneous abortion | Varies by product; includes stillbirth, congenital anomalies, neonatal death, developmental and genetic outcomes | [115–120] |
| **Novartis Multi-National Gilenya Pregnancy Exposure Registry in Multiple Sclerosis** | Global, including select middle-income countries (Brazil, Russia, Argentina) | Product focus: Medicines for multiple sclerosis (fingolimod)  Prospective  Retrospective  Pregnant women with multiple sclerosis exposed to fingolimod  Followed up to 23 months | Spontaneous abortions, stillbirths and elective terminations | Major congenital malformations, minor congenital malformations, physical developmental delays as well as adverse effects on immune system development in infants around one year of age | [121–123] |
| **Sanofi Pasteur Pregnancy Surveillance Program** | Global | Product focus: Vaccines (Menactra, Adacel, Fluzone, MenQuadfi (US only), Dengvaxia, Flublok (US only))  Prospective Retrospective  Pregnant women exposed to vaccine | Includes gestational diabetes, spontaneous abortion | Preterm birth, stillbirth, congenital anomalies  Developmental (physical and social) outcomes | [124–129] |
| **Electronic health records and other clinical software platforms** | | | | | |
| **Baobab Health Trust** | Malawi | Product focus: none specified  Prospective  All patients | None specified | None specified | [130,131] |
| **CHIRA (China Health Insurance Association) database** | China | Product focus: none specified  Prospective  Systematic sampling from public insurance databases, representing about 2% of total population | Thrombosis, gestational diabetes, gestational hypertension, PROM, preterm labor, pre-eclampsia/eclampsia, post-partum hemorrhage, corporeal infection, spontaneous abortion/miscarriage; maternal death, placenta previa | Preterm birth, stillbirth, small for gestational age, congenital anomalies | [132] |
| **DHIS2** | Palestine, Rwanda, Tanzania | Product focus: none specified  Prospective  All patients | Hospitalization, gestational diabetes, gestational hypertension, PROM, spontaneous abortion, preterm labor, pre-eclampsia/eclampsia, post-partum hemorrhage, dysfunctional labor, fetal growth retardation, maternal death | Neonatal death, congenital anomalies, neonatal infections, preterm birth, stillbirth, low birthweight, small for gestational age, respiratory distress.  Later infancy: Infections, respiratory illness | [133–140] |
| **SIP (Perinatal Informatic System)** | Latin America (multiple countries) | Product focus: none specified  Prospective  All pregnant patients | Thrombosis, gestational diabetes, gestational hypertension, PROM, preterm labor, pre-eclampsia/eclampsia, post-partum hemorrhage, corporeal infection, spontaneous abortion/miscarriage; maternal death, placenta previa | Preterm birth, stillbirth, small for gestational age, congenital anomalies | [141–145] |
| **SmartCare** | Zambia | Product focus: none specified  Prospective  All patients | None specified | None specified | [146–148] |
| **Western Cape Provincial Health Data Centre (PHDC)** | South Africa | Product focus: none specified  Prospective  All patients | None specified | None specified | [19,26,149–151] |

References for Table S1. Key characteristics of identified pregnancy exposure registries and related resources

[1] Slogrove A, Davies M-A. CHERISH (Children HIV Exposed Uninfected Research to Inform Survival and Health) n.d.

[2] Wyszynski DF, Bhattacharya M, Martínez-Pérez O, Scialli AR, Tassinari M, Bar-Zeev N, et al. The COVID-19 Vaccines International Pregnancy Exposure Registry (C-VIPER): Protocol and Methodological Considerations. Drug Saf 2023;46:297–308. https://doi.org/10.1007/s40264-022-01271-3.

[3] COVID-19 Vaccines International Pregnancy Exposure Registry (C-VIPER) n.d. https://c-viper.pregistry.com/ (accessed April 10, 2023).

[4] COVID-19 Vaccines International Pregnancy Exposure Registry - Full Text View - ClinicalTrials.gov n.d. https://clinicaltrials.gov/ct2/show/NCT04705116 (accessed June 29, 2023).

[5] Pregistry International Pregnancy Exposure Registry (PIPER) - Full Text View - ClinicalTrials.gov n.d. https://clinicaltrials.gov/ct2/show/NCT05352256 (accessed June 29, 2023).

[6] COVID-19 International Drug Pregnancy Registry (COVID-PR) n.d. https://covid-pr.pregistry.com/ (accessed April 10, 2023).

[7] COVID-19 International Drug Pregnancy Registry - Full Text View - ClinicalTrials.gov n.d. https://clinicaltrials.gov/ct2/show/NCT05013632 (accessed June 29, 2023).

[8] Wools-Kaloustian K. Measuring Adverse Pregnancy and Newborn Congenital Outcomes: An IeDEA Collaboration Study. clinicaltrials.gov; 2023.

[9] Oyungu E, El Kebbi O, Vreeman R, Nyandiko W, Monahan PO, Tu W, et al. Predicting neurodevelopmental risk in children born to mothers living with HIV in Kenya: protocol for a prospective cohort study (Tabiri Study). BMJ Open 2022;12:e061051. https://doi.org/10.1136/bmjopen-2022-061051.

[10] Malaria in Mothers and Babies Pregnancy Exposure Registry - MiMBa | LSTM n.d. https://www.lstmed.ac.uk/MiMBa (accessed April 10, 2023).

[11] Liverpool School of Tropical Medicine. A Pregnancy Registry to Assess the Safety of Antimalarial Use in Pregnancy. clinicaltrials.gov; 2021.

[12] Huang S, Tan J, Xiong YQ, Liu CR, Qi YN, Sun X. PRO15 A Brief Introduction of DATA Governance for Congenital Anomalies Based on the Xiamen Registry of Pregnant Women and Offspring (REPRESENT): A Population-Based, LONG-TERM and Follow-up Database. Value in Health Regional Issues 2020;22(Supplement):S97–8. https://doi.org/10.1016/j.vhri.2020.07.510.

[13] Tan J, Xiong Y, Qi Y, Liu C, Huang S, Yao G, et al. Data Resource Profile: Xiamen registry of pregnant women and offspring (REPRESENT): a population-based, long-term follow-up database linking four major healthcare data platforms. International Journal of Epidemiology 2021;50:27–8. https://doi.org/10.1093/ije/dyaa161.

[14] Xin S. Xiamen Registry of Pregnant Women and Offspring (REPRESENT): A Population-based, Long-term Follow-up Database Linking Four Major Healthcare Data Platforms. clinicaltrials.gov; 2020.

[15] Ubomi Buhle Project | Cape Town | Birth defects in South Africa – The Ubomi Buhle project is establishing a National Pregnancy Exposure Registry (NPER) in South Africa. The project monitors the exposures to medicines during pregnancy and poor birth outcomes from exposures to substances. n.d. https://ubomibuhle.org.za/ (accessed April 10, 2023).

[16] Mehta UC, van Schalkwyk C, Naidoo P, Ramkissoon A, Mhlongo O, Maharaj NR, et al. Birth outcomes following antiretroviral exposure during pregnancy: Initial results from a pregnancy exposure registry in South Africa. Southern African Journal of HIV Medicine 2019;20:971. https://doi.org/10.4102/sajhivmed.v20i1.971.

[17] Kalk E, Mehta U, Slogrove A, Jacob N, Myer L, Davies MA, et al. Pregnancy exposure registry/birth defects surveillance programme in the Western Cape, South Africa: A model for low- and middle-income countries. Drug Safety 2018;41(11):1212–3. https://doi.org/10.1007/s40264-018-0719-2.

[18] Kalk E, Heekes A, Slogrove AL, Phelanyane F, Davies M-A, Myer L, et al. Cohort profile: the Western Cape Pregnancy Exposure Registry (WCPER). BMJ Open 2022;12:e060205. https://doi.org/10.1136/bmjopen-2021-060205.

[19] van der Hoven J, Allen E, Cois A, de Waal R, Maartens G, Myer L, et al. Determining antenatal medicine exposures in South African women: a comparison of three methods of ascertainment. BMC Pregnancy and Childbirth 2022;22:466. https://doi.org/10.1186/s12884-022-04765-1.

[20] Ford N, Mofenson L, Kranzer K, Medu L, Frigati L, Mills EJ, et al. Safety of efavirenz in first-trimester of pregnancy: a systematic review and meta-analysis of outcomes from observational cohorts. AIDS 2010;24:1461–70. https://doi.org/10.1097/QAD.0b013e32833a2a14.

[21] Chammartin F, Dao Ostinelli CH, Anastos K, Jaquet A, Brazier E, Brown S, et al. International epidemiology databases to evaluate AIDS (IeDEA) in sub-Saharan Africa, 2012-2019. BMJ Open 2020;10:e035246. https://doi.org/10.1136/bmjopen-2019-035246.

[22] Ekouevi DK, Coffie PA, Ouattara E, Moh R, Amani-Bosse C, Messou E, et al. Pregnancy outcomes in women exposed to efavirenz and nevirapine: an appraisal of the IeDEA West Africa and ANRS Databases, Abidjan, Côte d’Ivoire. Journal of Acquired Immune Deficiency Syndromes 2011;56:183–7. https://doi.org/10.1097/QAI.0b013e3181ff04e6.

[23] Zaniewski E, Tymejczyk O, Kariminia A, Desmonde S, Leroy V, Ford N, et al. IeDEA–WHO Research-Policy Collaboration: contributing real-world evidence to HIV progress reporting and guideline development. J Virus Erad 2018;4:9–15.

[24] Batista CJB, Correa RG, Evangelista LR, Fleck K, Silva L, Renaud F, et al. The Brazilian experience of implementing the active pharmacovigilance of dolutegravir. Medicine (Baltimore) 2019;98:e14828. https://doi.org/10.1097/MD.0000000000014828.

[25] Msukwa MT, Keiser O, Jahn A, van Oosterhout JJ, Edmonds A, Phiri N, et al. Timing of combination antiretroviral therapy (cART) initiation is not associated with stillbirth among HIV-infected pregnant women in Malawi. Tropical Medicine & International Health 2019;24:727–35. https://doi.org/10.1111/tmi.13233.

[26] Kalk E, Heekes A, Mehta U, de Waal R, Jacob N, Cohen K, et al. Safety and Effectiveness of Isoniazid Preventive Therapy in Pregnant Women Living with Human Immunodeficiency Virus on Antiretroviral Therapy: An Observational Study Using Linked Population Data. Clinical Infectious Diseases 2020;71:e351–8. https://doi.org/10.1093/cid/ciz1224.

[27] Pereira GFM, Kim A, Jalil EM, Fonseca FF, Shepherd BE, Veloso VG, et al. Dolutegravir and pregnancy outcomes in women on antiretroviral therapy in Brazil: a retrospective national cohort study. The Lancet HIV 2021;8:e33–41. https://doi.org/10.1016/S2352-3018(20)30268-X.

[28] IeDEA International epidemiology Databases to Evaluate AIDS. IeDEA International Epidemiology Databases to Evaluate AIDS 2023. https://www.iedea.org/ (accessed April 10, 2023).

[29] East Africa International Epidemiology Database to evaluate AIDS (IeDEA) Regional Consortium n.d. https://reporter.nih.gov/search/QE33W75NjkuXdm4MZtAhfg/project-details/10239930 (accessed April 10, 2023).

[30] INDEPTH Network | Better Health Information for Better Health Policy n.d. http://www.indepth-network.org/ (accessed April 10, 2023).

[31] Dodoo ANO, Ako-Aduonvo S, Mshinda H, Mwisongo A, Binka FN. INESS - A new platform for evaluating anti-malarial drug safety and effectiveness in Africa. Drug Safety 2010;33(10):959. https://doi.org/10.2165/11532470-000000000-00000.

[32] Ndirangu J, Newell ML, Thorne C, Bland R. Treating HIV-infected mothers reduces under 5 years of age mortality rates to levels seen in children of HIV-uninfected mothers in rural South Africa. Antiviral Therapy 2012;17:81–90. https://doi.org/10.3851/imp1991.

[33] Odhiambo FO, Laserson KF, Sewe M, Hamel MJ, Feikin DR, Adazu K, et al. Profile: The KEMRI/CDC health and demographic surveillance system-Western Kenya. International Journal of Epidemiology 2012;41(4):977–87. https://doi.org/10.1093/ije/dys108.

[34] Oduro AR, Wak G, Azongo D, Debpuur C, Wontuo P, Kondayire F, et al. Profile of the Navrongo health and demographic surveillance system. International Journal of Epidemiology 2012;41(4):968–76. https://doi.org/10.1093/ije/dys111.

[35] Scott JAG, Bauni E, Moisi JC, Ojal J, Gatakaa H, Nyundo C, et al. Profile: The Kilifi health and demographic surveillance system (KHDSS). International Journal of Epidemiology 2012;41(3):650–7. https://doi.org/10.1093/ije/dys062.

[36] Alabi O, Doctor HV, Jumare A, Sahabi N, Abdulwahab A, Findley SE, et al. Health & demographic surveillance system profile: The Nahuche health and demographic surveillance system, northern Nigeria (Nahuche HDSS). International Journal of Epidemiology 2014;43(6):1770–80. https://doi.org/10.1093/ije/dyu197.

[37] Tinto H, Sevene E, Dellicour S, Calip GS, D’Alessandro U, Macete E, et al. Assessment of the safety of antimalarial drug use during early pregnancy (ASAP): Protocol for a multicenter prospective cohort study in Burkina Faso, Kenya and Mozambique. Reproductive Health 2015;12(1) (no pagination). https://doi.org/10.1186/s12978-015-0101-0.

[38] Dellicour S, Desai M, Aol G, Oneko M, Ouma P, Bigogo G, et al. Risks of miscarriage and inadvertent exposure to artemisinin derivatives in the first trimester of pregnancy: A prospective cohort study in western Kenya. Malaria Journal 2015;14(1) (no pagination). https://doi.org/10.1186/s12936-015-0950-6.

[39] Wak G, Williams J, Oduro A, Maure C, Zuber PLF, Black S. The safety of PsA-TT in pregnancy: An assessment performed within the navrongo health and demographic surveillance site in Ghana. Clinical Infectious Diseases 2015;61(Supplement 5):S489–92. https://doi.org/10.1093/cid/civ625.

[40] Arnaldo P, Rovira-Vallbona E, Langa JS, Salvador C, Guetens P, Chiheb D, et al. Uptake of intermittent preventive treatment and pregnancy outcomes: health facilities and community surveys in Chókwè district, southern Mozambique. Malaria Journal 2018;17:109. https://doi.org/10.1186/s12936-018-2255-z.

[41] Koné S, Hürlimann E, Baikoro N, Dao D, Bonfoh B, N’Goran EK, et al. Pregnancy-related morbidity and risk factors for fatal foetal outcomes in the Taabo health and demographic surveillance system, Côte d’Ivoire. BMC Pregnancy and Childbirth 2018;18:216. https://doi.org/10.1186/s12884-018-1858-2.

[42] Ambia J, Kabudula C, Risher K, Xavier Gómez-Olivé F, Rice BD, Etoori D, et al. Outcomes of patients lost to follow-up after antiretroviral therapy initiation in rural north-eastern South Africa. Trop Med Int Health 2019;24:747–56. https://doi.org/10.1111/tmi.13236.

[43] Thysen SM, Fernandes M, Benn CS, Aaby P, Fisker AB. Cohort profile: Bandim Health Project’s (BHP) rural Health and Demographic Surveillance System (HDSS) - A nationally representative HDSS in Guinea-Bissau. BMJ Open 2019;9(6) (no pagination). https://doi.org/10.1136/bmjopen-2018-028775.

[44] Waiswa P, Akuze J, Moyer C, Kwesiga D, Arthur S, Sankoh O, et al. Status of birth and pregnancy outcome capture in Health Demographic Surveillance Sites in 13 countries. Int J Public Health 2019;64:909–20. https://doi.org/10.1007/s00038-019-01241-0.

[45] Augusto O, Stergachis A, Dellicour S, Tinto H, Valá A, Ruperez M, et al. First trimester use of artemisinin-based combination therapy and the risk of low birth weight and small for gestational age. Malaria Journal 2020;19:144. https://doi.org/10.1186/s12936-020-03210-y.

[46] Rouamba T, Sondo P, Derra K, Nakanabo-Diallo S, Bihoun B, Rouamba E, et al. Optimal Approach and Strategies to Strengthen Pharmacovigilance in Sub-Saharan Africa: A Cohort Study of Patients Treated with First-Line Artemisinin-Based Combination Therapies in the Nanoro Health and Demographic Surveillance System, Burkina Faso. Drug Design, Development and Therapy 2020;14:1507–21. https://doi.org/10.2147/dddt.S224857.

[47] Argeseanu Cunningham S, Kwon C, Naser AM, Eilerts H, Reniers G. Pregnancy Surveillance Methods within Health and Demographic Surveillance Systems. Gates Open Research 2021;5 (no pagination). https://doi.org/10.12688/gatesopenres.13332.1.

[48] Kwesiga D, Tawiah C, Imam MA, Tesega AK, Nareeba T, Enuameh YAK, et al. Barriers and enablers to reporting pregnancy and adverse pregnancy outcomes in population-based surveys: EN-INDEPTH study. Popul Health Metr 2021;19:15. https://doi.org/10.1186/s12963-020-00228-x.

[49] NICHD DASH - Study Overview n.d. https://dash.nichd.nih.gov/study/20225 (accessed April 10, 2023).

[50] NICHD Global Network for Women’s and Children’s Health. Global Network for Women’s and Children’s Health Research Maternal Newborn Health Registry. clinicaltrials.gov; 2021.

[51] Althabe F, Moore JL, Gibbons L, Berrueta M, Goudar SS, Chomba E, et al. Adverse maternal and perinatal outcomes in adolescent pregnancies: The Global Network’s Maternal Newborn Health Registry study. Reprod Health 2015;12 Suppl 2:S8. https://doi.org/10.1186/1742-4755-12-s2-s8.

[52] Bucher S, Marete I, Tenge C, Liechty EA, Esamai F, Patel A, et al. A prospective observational description of frequency and timing of antenatal care attendance and coverage of selected interventions from sites in Argentina, Guatemala, India, Kenya, Pakistan and Zambia. Reprod Health 2015;12 Suppl 2:S12. https://doi.org/10.1186/1742-4755-12-s2-s12.

[53] Goudar SS, Carlo WA, McClure EM, Pasha O, Patel A, Esamai F, et al. The Maternal and Newborn Health Registry Study of the Global Network for Women’s and Children’s Health Research. International Journal of Gynaecology and Obstetrics 2012;118:190–3. https://doi.org/10.1016/j.ijgo.2012.04.022.

[54] Bose CL, Bauserman M, Goldenberg RL, Goudar SS, McClure EM, Pasha O, et al. The Global Network Maternal Newborn Health Registry: a multi-national, community-based registry of pregnancy outcomes. Reprod Health 2015;12 Suppl 2:S1. https://doi.org/10.1186/1742-4755-12-s2-s1.

[55] Goudar SS, Stolka KB, Koso-Thomas M, Honnungar NV, Mastiholi SC, Ramadurg UY, et al. Data quality monitoring and performance metrics of a prospective, population-based observational study of maternal and newborn health in low resource settings. Reprod Health 2015;12 Suppl 2:S2. https://doi.org/10.1186/1742-4755-12-s2-s2.

[56] Kodkany BS, Derman RJ, Honnungar NV, Tyagi NK, Goudar SS, Mastiholi SC, et al. Establishment of a Maternal Newborn Health Registry in the Belgaum District of Karnataka, India. Reprod Health 2015;12 Suppl 2:S3. https://doi.org/10.1186/1742-4755-12-s2-s3.

[57] McClure EM, Saleem S, Goudar SS, Moore JL, Garces A, Esamai F, et al. Stillbirth rates in low-middle income countries 2010 - 2013: a population-based, multi-country study from the Global Network. Reprod Health 2015;12 Suppl 2:S7. https://doi.org/10.1186/1742-4755-12-s2-s7.

[58] Pasha O, Saleem S, Ali S, Goudar SS, Garces A, Esamai F, et al. Maternal and newborn outcomes in Pakistan compared to other low and middle income countries in the Global Network’s Maternal Newborn Health Registry: an active, communitybased, pregnancy surveillance mechanism. Reproductive Health 2015;12:1–10.

[59] McClure EM, Garces A, Saleem S, Moore JL, Bose CL, Esamai F, et al. Global Network for Women’s and Children’s Health Research: probable causes of stillbirth in low- and middle-income countries using a prospectively defined classification system. BJOG: An International Journal of Obstetrics and Gynaecology 2018;125:131–8. https://doi.org/10.1111/1471-0528.14493.

[60] Saleem S, Tikmani SS, McClure EM, Moore JL, Azam SI, Dhaded SM, et al. Trends and determinants of stillbirth in developing countries: results from the Global Network’s Population-Based Birth Registry. Reprod Health 2018;15:100. https://doi.org/10.1186/s12978-018-0526-3.

[61] Aghai ZH, Goudar SS, Patel A, Saleem S, Dhaded SM, Kavi A, et al. Gender variations in neonatal and early infant mortality in India and Pakistan: a secondary analysis from the Global Network Maternal Newborn Health Registry. Reprod Health 2020;17:178. https://doi.org/10.1186/s12978-020-01028-0.

[62] Billah SM, Haque R, Chowdhury AI, Siraj MS, Rahman QS, Hossain T, et al. Setting up a maternal and newborn registry applying electronic platform: an experience from the Bangladesh site of the global network for women’s and children’s health. Reproductive Health 2020;17(Supplement 2) (no pagination). https://doi.org/10.1186/s12978-020-00993-w.

[63] Goudar SS, Goco N, Somannavar MS, Kavi A, Vernekar SS, Tshefu A, et al. Institutional deliveries and stillbirth and neonatal mortality in the Global Network’s Maternal and Newborn Health Registry. Reprod Health 2020;17:179. https://doi.org/10.1186/s12978-020-01001-x.

[64] McClure EM, Garces AL, Hibberd PL, Moore JL, Goudar SS, Saleem S, et al. The Global Network Maternal Newborn Health Registry: a multi-country, community-based registry of pregnancy outcomes. Reproductive Health 2020;17:184. https://doi.org/10.1186/s12978-020-01020-8.

[65] Short VL, Hoffman M, Metgud M, Kavi A, Goudar SS, Okitawutshu J, et al. Safety of daily low-dose aspirin use during pregnancy in low-income and middle-income countries. AJOG Global Reports 2021;1:100003. https://doi.org/10.1016/j.xagr.2021.100003.

[66] Naqvi S, Naqvi F, Saleem S, Thorsten VR, Figueroa L, Mazariegos M, et al. Health Care in Pregnancy During the COVID-19 Pandemic and Pregnancy Outcomes in Six Low-and-Middle-Income Countries: Evidence from a Prospective, Observational Registry of the Global Network for Women’s and Children’s Health. BJOG: An International Journal of Obstetrics and Gynaecology 2022. https://doi.org/10.1111/1471-0528.17175.

[67] Naqvi S, Saleem S, Naqvi F, Billah SM, Nielsen E, Fogleman E, et al. Knowledge, attitudes, and practices of pregnant women regarding COVID-19 vaccination in pregnancy in 7 low- and middle-income countries: An observational trial from the Global Network for Women and Children’s Health Research. BJOG: An International Journal of Obstetrics and Gynaecology 2022. https://doi.org/10.1111/1471-0528.17226.

[68] Welcome to PREPARE - St George’s, University of London 2019. https://gbsprepare.org/ (accessed April 10, 2023).

[69] SMRU | Shoklo Malaria Research Unit n.d. https://www.shoklo-unit.com/ (accessed April 10, 2023).

[70] Moore KA, Simpson JA, Paw MK, Pimanpanarak M, Wiladphaingern J, Rijken MJ, et al. Safety of artemisinins in first trimester of prospectively followed pregnancies: an observational study. The Lancet Infectious Diseases 2016;16:576–83. https://doi.org/10.1016/S1473-3099(15)00547-2.

[71] Saito M, Dahal P, Tyrosvoutis MEG, Stepniewska K, Humphreys GS, Paw MK, et al. Optimal duration of follow-up for assessing efficacy of drugs for uncomplicated falciparum malaria in pregnancy in Asia: A systematic review and individual patient data metaanalysis. Tropical Medicine and International Health 2017;22(Supplement 1):95. https://doi.org/10.1111/%28ISSN%291365-3156.

[72] Gadoth A, Mukadi Nkamba D, Arena PJ, Hoff NA, Dzogang C, Kampilu D, et al. Assessing the feasibility of passive surveillance for maternal immunization safety utilizing archival medical records in Kinshasa, Democratic Republic of the Congo. Vaccine 2022;40:3605–13. https://doi.org/10.1016/j.vaccine.2022.04.073.

[73] Arena PJ, Dzogang C, Gadoth A, Nkamba DM, Hoff NA, Kampilu D, et al. Comparison of adverse pregnancy and birth outcomes using archival medical records before and during the first wave of the COVID-19 pandemic in Kinshasa, Democratic Republic of Congo: a facility-based, retrospective cohort study. BMC Pregnancy and Childbirth 2023;23:31. https://doi.org/10.1186/s12884-022-05291-w.

[74] Garcia AM, Machicado S, Gracia G, Zarante IM. Risk factors for congenital diaphragmatic hernia in the Bogota birth defects surveillance and follow-up program, Colombia. Pediatric Surgery International 2016;32(3):227–34. https://doi.org/10.1007/s00383-015-3832-7.

[75] ECLAMC - Latin American Colaborative Study of Congenital Malformations n.d. http://www.eclamc.org/eng/index.php (accessed April 10, 2023).

[76] Castilla EE, Orioli IM. ECLAMC: The Latin-American Collaborative Study of Congenital Malformations. PHG 2004;7:76–94. https://doi.org/10.1159/000080776.

[77] CHAMPS - We Build Knowledge to Save Children’s Lives. CHAMPS Health n.d. https://champshealth.org/ (accessed April 10, 2023).

[78] Zhou Y, Tao J, Wang K, Deng K, Wang Y, Zhao J, et al. Protocol of a prospective and multicentre China Teratology Birth Cohort (CTBC): association of maternal drug exposure during pregnancy with adverse pregnancy outcomes. BMC Pregnancy and Childbirth 2021;21(1) (no pagination). https://doi.org/10.1186/s12884-021-04073-0.

[79] Assessment of Birth Outcomes in Eswatini after Transition to Dolutegravir-based Treatment - EGPAF. Elizabeth Glaser Pediatric AIDS Foundation n.d. https://pedaids.org/research/assessment-of-birth-outcomes-in-eswatini-after-transition-to-dolutegravir-based-treatment/ (accessed April 10, 2023).

[80] NEURAL TUBE AND OTHER BIRTH DEFECTS BY HIV STATUS AND ART REGIMEN IN ESWATINI. CROI Conference n.d. https://www.croiconference.org/abstract/neural-tube-and-other-birth-defects-by-hiv-status-and-art-regimen-in-eswatini/ (accessed April 10, 2023).

[81] Mumpe-Mwanja D, Barlow-Mosha L, Williamson D, Valencia D, Serunjogi R, Kakande A, et al. A hospital-based birth defects surveillance system in Kampala, Uganda. BMC Pregnancy and Childbirth 2019;19:372. https://doi.org/10.1186/s12884-019-2542-x.

[82] Barlow-Mosha L, Mumpe DM, Williamson D, Valencia D, Serunjogi R, Matovu JN, et al. Neural tube defects, HIV, and antiretrovirals: Birth-defect surveillance in Uganda. Topics in Antiviral Medicine 2019;27(SUPPL 1):285s–6s.

[83] Barlow-Mosha L, Serunjogi R, Kalibbala D, Mumpe-Mwanja D, Williamson D, Valencia D, et al. Prevalence of neural tube defects, maternal HIV status, and antiretroviral therapy from a hospital-based birth defect surveillance in Kampala, Uganda. Birth Defects Res 2022;114:95–104. https://doi.org/10.1002/bdr2.1964.

[84] Birth Defects Surveillance in Malawi. I-TECH 2022. https://www.go2itech.org/2022/03/birth-defects-surveillance-in-malawi/ (accessed April 10, 2023).

[85] Birth Defects Surveillance in Malawi - Project brief n.d.

[86] Albano JD, Vannappagari V, Scheuerle A, Watts H, Thorne C, Ng L, et al. Insti exposure and neural tube defects: Data from antiretroviral pregnancy registry. Topics in Antiviral Medicine 2019;27(SUPPL 1):287s.

[87] Caniglia EC, Abrams J, Diseko M, Mayondi G, Mabuta J, Makhema J, et al. Seasonality of adverse birth outcomes in women with and without HIV in a representative birth outcomes surveillance study in Botswana. BMJ Open 2021;11:e045882. https://doi.org/10.1136/bmjopen-2020-045882.

[88] Hill A, Van De Ven NS, Pozniak A, Levi JA, Garratt A, Redd C, et al. Reports of neuraltube defects for 8 arts, in FDA, who, EMA, and UK safety databases. Topics in Antiviral Medicine 2019;27(SUPPL 1):286s–7s.

[89] Raesima MM, Ogbuabo CM, Thomas V, Forhan SE, Gokatweng G, Dintwa E, et al. Dolutegravir use at conception - Additional surveillance data from Botswana. New England Journal of Medicine 2019;381(9) (no pagination). https://doi.org/10.1056/NEJMc1908155.

[90] Zash R, Caniglia EC, Diseko M, Mayondi G, Mabuta J, Luckett R, et al. Maternal weight and birth outcomes among women on antiretroviral treatment from conception in a birth surveillance study in Botswana. Journal of the International AIDS Society 2021;24(6) (no pagination). https://doi.org/10.1002/jia2.25763.

[91] Zash R, Holmes L, Diseko M, Jacobson DL, Brummel S, Mayondi G, et al. Neural-Tube Defects and Antiretroviral Treatment Regimens in Botswana. New England Journal of Medicine 2019;381:827–40. https://doi.org/10.1056/NEJMoa1905230.

[92] Birth Outcomes Surveillance Study: Tsepamo Study | Botswana Harvard Partnership n.d. https://bhp.org.bw/node/52 (accessed April 15, 2023).

[93] Thomas SV, Indrani L, Devi GC, Jacob S, Beegum J, Jacob PP, et al. Pregnancy in women with epilepsy: Preliminary results of Kerala registry of epilepsy and pregnancy. Neurology India 2001;49(1):60–6.

[94] Thomas SV, Ajaykumar B, Sindhu K, Nair MKC, George B, Sarma PS. Motor and mental development of infants exposed to antiepileptic drugs in utero. Epilepsy & Behavior 2008;13:229–36. https://doi.org/10.1016/j.yebeh.2008.01.010.

[95] Tomson T, Battino D, Craig J, Hernandez-Diaz S, Holmes LB, Lindhout D, et al. Pregnancy registries: Differences, similarities, and possible harmonization. Epilepsia 2010;51:909–15. https://doi.org/10.1111/j.1528-1167.2010.02525.x.

[96] Thomas SV, Syam UK, Asha PT, Sucharitha DJ, Sabarinathan S, Sarma PS. Pregnancy outcome and fetal malformation risk of women with epilepsy: Pregnancy registry based experience from Kerala, India. Birth Defects Research Part A - Clinical and Molecular Teratology 2011;91(5):388. https://doi.org/10.1002/bdra.20834.

[97] Thomas SV, Jose M, Divakaran S, Sankara Sarma P. Malformation risk of antiepileptic drug exposure during pregnancy in women with epilepsy: Results from a pregnancy registry in South India. Epilepsia 2017;58(2):274–81. https://doi.org/10.1111/epi.13632.

[98] Keni RR, Jose M, A.S R, Baishya J, Sankara Sarma P, Thomas SV. Anti-epileptic drug and folic acid usage during pregnancy, seizure and malformation outcomes: Changes over two decades in the Kerala Registry of Epilepsy and Pregnancy. Epilepsy Research 2020;159:106250. https://doi.org/10.1016/j.eplepsyres.2019.106250.

[99] Seshachala BB, Jose M, Lathikakumari AM, Murali S, Kumar AS, Thomas SV. Valproate usage in pregnancy: An audit from the Kerala Registry of Epilepsy and Pregnancy. Epilepsia 2021;62:1141–7. https://doi.org/10.1111/epi.16882.

[100] Thomas SV, Jeemon P, Pillai R, Jose M, Lalithakumari AM, Murali S, et al. Malformation risk of new anti-epileptic drugs in women with epilepsy; observational data from the Kerala registry of epilepsy and pregnancy (KREP). Seizure - European Journal of Epilepsy 2021;93:127–32. https://doi.org/10.1016/j.seizure.2021.10.015.

[101] Avila WS, Rivera MAM, Marques-Santos C, Rivera IR, Costa MENC, Lucena AJG de, et al. The REBECGA Brazilian Registry of Pregnancy and Heart Disease: Rationale and Design. Int j Cardiovasc Sci (Impr) 2021;34:452–8. https://doi.org/10.36660/ijcs.20200419.

[102] Gnanaraj JP, Princy SA, Sliwa-Hahnle K, Sathyendra S, Jeyabalan N, Sethumadhavan R, et al. Tamil Nadu Pregnancy and Heart Disease Registry (TNPHDR): design and methodology. BMC Pregnancy and Childbirth 2022;22:80. https://doi.org/10.1186/s12884-021-04305-3.

[103] Tilson HH. Using Registries to Monitor HIV Pre-Exposure Prophylaxis Safety in Clinical Settings. American Journal of Preventive Medicine 2013;44:S151–5. https://doi.org/10.1016/j.amepre.2012.09.042.

[104] Wang L, Kourtis AP, Ellington S, Legardy-Williams J, Bulterys M. Safety of Tenofovir During Pregnancy for the Mother and Fetus: A Systematic Review. Clinical Infectious Diseases 2013;57:1773–81. https://doi.org/10.1093/cid/cit601.

[105] Vannappagari V, Koram N, Albano J, Tilson H, Gee C. Abacavir and Lamivudine Exposures During Pregnancy and Non-defect Adverse Pregnancy Outcomes: Data From the Antiretroviral Pregnancy Registry. JAIDS Journal of Acquired Immune Deficiency Syndromes 2015;68.

[106] Vannappagari V, Albano JD, Koram N, Tilson H, Scheuerle AE, Napier MD. Prenatal exposure to zidovudine and risk for ventricular septal defects and congenital heart defects: data from the Antiretroviral Pregnancy Registry. European Journal of Obstetrics, Gynecology, and Reproductive Biology 2016;197:6–10. https://doi.org/10.1016/j.ejogrb.2015.11.015.

[107] Mofenson LM, Vannappagari V, Scheuerle AE, Baugh B, Beckerman KP, Betman H, et al. Periconceptional antiretroviral exposure and central nervous system (CNS) and neural tube birth defects-data from Antiretroviral Pregnancy Registry (APR). Journal of the International AIDS Society Conference: 10th IAS Conference on HIV Science Mexico City Mexico 2019;22. https://doi.org/10.1002/jia2.25327.

[108] Hellwig K, Duarte Caron F, Wicklein E-M, Bhatti A, Adamo A. Pregnancy outcomes from the global pharmacovigilance database on interferon beta-1b exposure. Therapeutic Advances in Neurological Disorders 2020;13:1756286420910310. https://doi.org/10.1177/1756286420910310.

[109] Beghi E, Annegers JF, Epilepsy for the CGPR in. Pregnancy Registries in Epilepsy. Epilepsia 2001;42:1422–5. https://doi.org/10.1046/j.1528-1157.2001.11201.x.

[110] Gogatishvili N, Ediberidze T, Mamukadze S, Lomidze G, Tatishvili N, Kasradze S. Cognitive outcomes of children with fetal antiepileptic drug exposure at the age of 3-6 years-preliminary data. European Journal of Neurology 2015;1):329. https://doi.org/10.1111/ene.12807.

[111] The EURAP Study Group. Utilization of antiepileptic drugs during pregnancy: Comparative patterns in 38 countries based on data from the EURAP registry. Epilepsia 2009;50:2305–9. https://doi.org/10.1111/j.1528-1167.2009.02093.x.

[112] Tomson T, Battino D, Bonizzoni E, Craig J, Lindhout D, Sabers A, et al. Dose-dependent risk of malformations with antiepileptic drugs: an analysis of data from the EURAP epilepsy and pregnancy registry. The Lancet Neurology 2011;10:609–17. https://doi.org/10.1016/S1474-4422(11)70107-7.

[113] Kochen S, Salera C, Seni J. Pregnant women with epilepsy in a developing country. Open Neurology Journal 2011;5(1):63–7. https://doi.org/10.2174/1874205X01105010063.

[114] EURAP - International Registry of Antiepileptic Drugs and Pregnancy. EURAP n.d. https://eurapinternational.org/ (accessed April 10, 2023).

[115] Tennis P, Eldridge RR, International Lamotrigine Pregnancy Registry Scientific Advisory Committee. Preliminary Results on Pregnancy Outcomes in Women Using Lamotrigine. Epilepsia 2002;43:1161–7. https://doi.org/10.1046/j.1528-1157.2002.45901.x.

[116] GlaxoSmithKline Pregnancy Registries n.d. https://pregnancyregistry.gsk.com/ (accessed April 10, 2023).

[117] GlaxoSmithKline. MENVEO Pregnancy Registry: an Observational Study on the Safety of MENVEO Exposure in Pregnant Women and Their Offspring. clinicaltrials.gov; 2019.

[118] GlaxoSmithKline. Fluarix/ FluLaval/ Fluarix Quadrivalent/ FluLaval Quadrivalent Pregnancy Registry: a Prospective, Exploratory, Cohort Study to Detect and Describe Abnormal Pregnancy Outcomes in Women Intentionally or Unintentionally Vaccinated With Fluarix or Fluarix Quadrivalent or FluLaval or FluLaval Quadrivalent During Pregnancy or Within 28 Days Preceding Conception. clinicaltrials.gov; 2020.

[119] GlaxoSmithKline. Twinrix [Hepatitis A Inactivated & Hepatitis B (Recombinant) Vaccine] Pregnancy Registry. clinicaltrials.gov; 2018.

[120] GlaxoSmithKline. A Post-marketing, Observational, Retrospective, Cohort Study to Assess the Safety of RefortrixTM (Tdap) When Administered During Pregnancy in a Maternal Immunization Program in Brazil. clinicaltrials.gov; 2019.

[121] The Gilenya Pregnancy Registry. Novartis Recruiting Clinical Trials n.d. https://www.recruiting-trials.novartis.com/clinicaltrials/study/nct01285479 (accessed April 10, 2023).

[122] The Gilenya Pregnancy Registry. Novartis n.d. https://www.novartis.com/clinicaltrials/study/nct01285479 (accessed June 29, 2023).

[123] GILENYA REGISTRY n.d. https://www.gilenyapregnancyregistry.com/ (accessed June 29, 2023).

[124] Khromava A, Cohen CJ, Mazur M, Kanesa-thasan N, Crucitti A, Seifert H. Manufacturers’ postmarketing safety surveillance of influenza vaccine exposure in pregnancy. American Journal of Obstetrics and Gynecology 2012;207:S52–6. https://doi.org/10.1016/j.ajog.2012.06.074.

[125] Ledlie S, Gandhi-Banga S, Shrestha A, Mallett Moore T, Khromava A. Exposure to quadrivalent influenza vaccine during pregnancy: Results from a global pregnancy registry. Influenza and Other Respiratory Viruses 2022;16:90–100. https://doi.org/10.1111/irv.12897.

[126] Sanofi Pasteur Pregnancy Registries n.d. https://www.sanofipasteurpregnancyregistry.com/ (accessed April 10, 2023).

[127] Sanofi Pasteur, a Sanofi Company. Sanofi Pasteur Quadrivalent Influenza Vaccine Pregnancy Registry Protocol. clinicaltrials.gov; 2020.

[128] Sanofi Pasteur, a Sanofi Company. Sanofi Pasteur Quadrivalent Intradermal Influenza Vaccine Pregnancy Registry Protocol. clinicaltrials.gov; 2020.

[129] Sanofi Pasteur, a Sanofi Company. A Pregnancy Registry to Evaluate the Safety of Dengue Vaccine Among Inadvertently Exposed Pregnant Women and Their Offsprings (DNG16). clinicaltrials.gov; 2023.

[130] Haas AD, van Oosterhout JJ, Tenthani L, Jahn A, Zwahlen M, Msukwa MT, et al. HIV transmission and retention in care among HIV-exposed children enrolled in Malawi’s prevention of mother-to-child transmission programme. Journal of the International AIDS Society 2017;20:21947. https://doi.org/10.7448/ias.20.1.21947.

[131] Home - Baobab Health Trust 2022. https://baobabhealthtrust.org/ (accessed April 10, 2023).

[132] Zhang J, Ung COL, Guan X, Shi L. Safety of medication use during pregnancy in mainland China: Based on a national health insurance database in 2015. BMC Pregnancy and Childbirth 2019;19(1) (no pagination). https://doi.org/10.1186/s12884-019-2622-y.

[133] Dehnavieh R, Haghdoost A, Khosravi A, Hoseinabadi F, Rahimi H, Poursheikhali A, et al. The District Health Information System (DHIS2): A literature review and meta-synthesis of its strengths and operational challenges based on the experiences of 11 countries. HIM J 2019;48:62–75. https://doi.org/10.1177/1833358318777713.

[134] DHIS2. DHIS2 n.d. https://dhis2.org/ (accessed April 15, 2023).

[135] Wilms MC, Mbembela O, Prytherch H, Hellmold P, Kuelker R. An in-depth, exploratory assessment of the implementation of the National Health Information System at a district level hospital in Tanzania. BMC Health Services Research 2014;14:91. https://doi.org/10.1186/1472-6963-14-91.

[136] Venkateswaran M, Mørkrid K, Khader KA, Awwad T, Friberg IK, Ghanem B, et al. Comparing individual-level clinical data from antenatal records with routine health information systems indicators for antenatal care in the West Bank: A cross-sectional study. PLOS ONE 2018;13:e0207813. https://doi.org/10.1371/journal.pone.0207813.

[137] Venkateswaran M, Ghanem B, Abbas E, Khader KA, Ward IA, Awwad T, et al. A digital health registry with clinical decision support for improving quality of antenatal care in Palestine (eRegQual): a pragmatic, cluster-randomised, controlled, superiority trial. The Lancet Digital Health 2022;4:e126–36. https://doi.org/10.1016/S2589-7500(21)00269-7.

[138] Hassan S, Vikanes A, Laine K, Zimmo K, Zimmo M, Bjertness E, et al. Building a research registry for studying birth complications and outcomes in six Palestinian governmental hospitals. BMC Pregnancy Childbirth 2017;17:112. https://doi.org/10.1186/s12884-017-1296-6.

[139] Dias B. DHIS2 tracker e-Registry in Palestine. DHIS2 2020. https://dhis2.org/palestine-tracker-user-story/ (accessed April 10, 2023).

[140] Maternal and Child Health e-Registry n.d. https://www.pniph.org/index.php/en/component/content/article/93-health-systems/167-maternal-and-child-health-e-registry (accessed April 10, 2023).

[141] Perinatal Information System - PAHO/WHO | Pan American Health Organization n.d. https://www.paho.org/en/latin-american-center-perinatology-women-and-reproductive-health-clap/perinatal-information-system (accessed April 15, 2023).

[142] Karolinski A, Mercer R, Bolzán A, Salgado P, Ocampo C, Nieto R, et al. Bases para el desarrollo e implementación de un modelo de información en salud de la mujer y perinatal orientado a la gestión en Latinoamérica. Rev Panam Salud Publica 2018;42:e148. https://doi.org/10.26633/RPSP.2018.148.

[143] Bradley H, Tapia V, Kamb ML, Newman LM, Garcia PJ, Serruya SJ, et al. Can the Perinatal Information System in Peru be used to measure the proportion of adverse birth outcomes attributable to maternal syphilis infection? Rev Panam Salud Publica 2014;36:73–9.

[144] Serruya SJ, Duran P, Martinez G, Romero M, Caffe S, Alonso M, et al. Maternal and congenital syphilis in selected Latin America and Caribbean countries: a multi-country analysis using data from the Perinatal Information System. Sex Health 2015;12:164–9. https://doi.org/10.1071/SH14191.

[145] Fescina R. History of the Perinatal Information System. Making Pregnancy Safer. 2010.

[146] Gumede-Moyo S, Todd J, Schaap A, Mee P, Filteau S. Effect of prevention of mother-to-child transmission strategies on antiretroviral therapy coverage in pregnant women in Zambia: analysis using routinely collected data (2010-15). The Lancet Global Health 2019;7(Supplement 1):S25. https://doi.org/10.1016/S2214-109X%2819%2930110-X.

[147] Gumede-Moyo S, Todd J, Bond V, Mee P, Filteau S. A qualitative inquiry into implementing an electronic health record system (SmartCare) for prevention of mother-to-child transmission data in Zambia: a retrospective study. BMJ Open 2019;9:e030428. https://doi.org/10.1136/bmjopen-2019-030428.

[148] Kaumba PC. Factors affecting the implementation of the SmartCare EHR system in Zambia. Social Sciences & Humanities Open 2023;7:100399. https://doi.org/10.1016/j.ssaho.2023.100399.

[149] Boulle A, Vega I de, Moodley M, Shand L, Loff A, Fredericks N, et al. Data Centre Profile: The Provincial Health Data Centre of the Western Cape Province, South Africa. International Journal of Population Data Science 2019;4. https://doi.org/10.23889/ijpds.v4i2.1143.

[150] Mehta U, Heekes A, Hons Bm, Kalk E, Boulle A. Assessing the value of Western Cape Provincial Government health administrative data and electronic pharmacy records in ascertaining medicine use during pregnancy. S Afr Med J 2018;108:439–43.

[151] Mehta U, Smith M, Kalk E, Hayes H, Swart A, Tucker L, et al. Understanding and Responding to Prescribing Patterns of Sodium Valproate-Containing Medicines in Pregnant Women and Women of Childbearing Age in Western Cape, South Africa. Drug Safety 2021;44(1):41–51. https://doi.org/10.1007/s40264-020-00987-4.
