# Supplementary material for: Landscape analysis of pregnancy exposure registries in low- and middle-income countries: a scoping review
Source: BMJ Open. 2025 Oct 29;15(10):e097198. doi: 10.1136/bmjopen-2024-097198 (PMC12574391; doi:10.1136/bmjopen-2024-097198)
Supplement: Supplementary file 3 [file bmjopen-2024-097198.reviewer_comments.pdf]

PEER REVIEW HISTORY

BMJ Open publishes all reviews undertaken for accepted manuscripts. Reviewers are asked to complete a checklist review form and are provided with free text boxes to elaborate on their assessment. These free text comments are reproduced below.

ARTICLE DETAILS

Title (Provisional)

Landscape Analysis of Pregnancy Exposure Registries in Low- and Middle-Income Countries: a Scoping Review.

Authors

Bhat, Niranjan; Nakambale, Hilma; Salts, Nancy; Lamprianou, Smaragda; Knudson, Sophie; AbuShweimeh, Rahmeh; Mooney, Jessica; Mehta, Ushma C.; Sevene, Esperanca; Fell, Deshayne; Pal, Shanthi Narayan; Stergachis, Andy

---

VERSION 1 - REVIEW

Reviewer 1

Name Lee, Jessica

Affiliation Harvard T.H. Chan School of Public Health, Center for Biostatistics in AIDS Research

Date 05-May-2025

COI None

This review was well-written and comprehensive in summarizing details on resource types and strengths and weaknesses. A few minor questions for clarification are below:

How did the authors identify the key informants/experts, particularly what was the rationale for reaching out to individuals within the WHO PIDM or ESC? Were any other experts outside of these groups considered? And what are some questions on the survey/semi-structured interview?

Were only sources in English considered or were other languages also assessed and reviewed?

Line 198 states that the "amount of detail on which outcomes were monitored varied", but the following sentence doesn't seem to outline how the amount of details differed. Could the authors elaborate?

A general thought regarding the secondary research questions listed in the protocol paper was that these questions could benefit from deeper discussion.

-Regarding the first secondary question, "Can the PERs that have been identified in LMICs be used or adapted to monitor additional new vaccines or drugs that may be introduced for pregnant women?": how feasible or smoothly could new exposures be incorporated? The authors state most of the registries could be strengthened or modified, but what conditions would need to be met, and what obstacles exist in other registries identified that would prevent this?

-Regarding the other secondary question: "What is the potential for data harmonisation and/or combining of data across databases and registries?": it is understandable more details of each of the registries would be needed to determine potential for harmonization, so what did the authors hope to be able to answer for this question? What are some considerations the authors think are needed to harmonize data? For example, if there are multiple registries in the same region, I'm wondering if it would be possible to identify overlapping individuals and how to account for that.

Minor comment on Table 2 to ensure consistent cell alignment throughout. Would it also be possible to add details on the list of countries if the registries/systems are "multinational" or "global", unless the list of countries is very expansive? Lastly, what is the difference between blank cells and cells with "N/A"?

Figure 1: It is not clear what the difference between some of the exclusion reasons are, such as "No pregnancy outcomes" vs. "Wrong outcomes", "Overview/summary/methods paper" vs. "Overview/summary paper", and "Wrong patient population" vs. "Pediatric population". Why was "wrong comparator" a reason for exclusion?

Figure 2 is a very nice visual representation. I would just advise making sure it is accessible to color-blindedness and the legend being inclusive (I don't think the red diagonal lines is in the legend unless I'm misunderstanding the legend).

**Reviewer**	**2**
**Name**	**SoleimanvandiAzar, Neda**
**Affiliation**	**Iran University of Medical Sciences**
**Date**	**08-Jun-2025**
**COI**	**None**

This research was conducted as a Scoping Review on the Landscape Analysis of Pregnancy Exposure Registries in Low- and Middle-Income Countries. I thank the authors of the article for choosing this topic and writing this manuscript.

However, there are few points that it is recommended to be addressed as far as possible.

1- Title: The title was representative of the type of study.

2- Abstract: It was well written.

3- Introduction: It was well written.

4- Material and Methods: It was rather well written. Would the authors explain why the didn't search in web of Science and Scopus?

5- Results: It was well written.

6- Tables: They were Illustrative

7- Figures: They were Illustrative

8- Discussion: It was well written.

9- Limitation: The authors didn't mention the limitations of the study at the end of the discussion..

10- Conclusion: It was well written, and it was according to the results.

VERSION 1 - AUTHOR RESPONSE

Reviewer: 1
Ms. Jessica Lee, Harvard T.H. Chan School of Public Health
Comments to the Author:
This review was well-written and comprehensive in summarizing details on resource types and strengths and weaknesses. A few minor questions for clarification are below:

How did the authors identify the key informants/experts, particularly what was the rationale for reaching out to individuals within the WHO PIDM or ESC? Were any other experts outside of these groups considered?

PIDM members are the pharmacovigilance authorities for their respective countries, and are therefore expected to be knowledgeable about PER's in their area because they would likely be operators, stakeholders, and/or at least users or recipients of the data. We considered that it would be likely that teams that run PERs in a country would consult with national PV staff during the establishment or operation of the registries. The ESC is a group of experts in maternal immunization safety who are members of the following WHO advisory committees: Strategic Advisory Group of Experts on Immunization (SAGE), Advisory Committee on Safety of Medicinal Products (ACSoMP), Global Advisory Committee on Vaccine Safety (GACSV) and Mother and Newborn Information for Tracking Outcomes & Results. ESC members assessed and advised WHO on a number of topics managed by the pharmacovigilance team, including this study. Additional experts were identified to receive the survey based on the published and grey literature review as well as personal referrals. Additional language has been included in the manuscript.

And what are some questions on the survey/semi-structured interview?

The survey/interview questions are now included as an appendix/supplementary information.

Were only sources in English considered or were other languages also assessed and reviewed?

Used sources in languages available on the listed databases, which did include some Spanish and Portuguese. Abstracts and full-text were translated for data abstraction.

Line 198 states that the "amount of detail on which outcomes were monitored varied", but the following sentence doesn't seem to outline how the amount of details differed. Could the authors elaborate?

Text has been added in the revised manuscript to address this comment.

A general thought regarding the secondary research questions listed in the protocol paper was that these questions could benefit from deeper discussion.
-Regarding the first secondary question, "Can the PERs that have been identified in LMICs be used or adapted to monitor additional new vaccines or drugs that may be introduced for pregnant women?": how feasible or smoothly could new exposures be incorporated? The authors state most of the registries could be strengthened or modified, but what conditions would need to be met, and what obstacles exist in other registries identified that would prevent this?

Criteria to determine whether the described system could be utilized or adapted for future PER include whether the system was currently operational, designed with flexibility to accommodate new vaccines or drugs, capable of timely identification of adverse events, employ electronic or mobile tools for data collection and reporting, incorporate mechanisms for follow-up and reminders, and had prior use in post-marketing safety surveillance.

The ease with which new exposures could be added can also vary based on the complexity of the exposure, such as if multiple doses are given, or if the drug or vaccine can be administered at different locations, or if additional verifications are needed.

-Regarding the other secondary question: "What is the potential for data harmonisation and/or combining of data across databases and registries?": it is understandable more details of each of the registries would be needed to determine potential for harmonization, so what did the authors hope to be able to

answer for this question? What are some considerations the authors think are needed to harmonize data? For example, if there are multiple registries in the same region, I'm wondering if it would be possible to identify overlapping individuals and how to account for that.

One consideration would be whether the registry uses the standardized case definitions developed by the Brighton Collaboration and the GAIA initiative, as they can promote harmonization, enhance reproducibility, and ensure scientifically robust assessments of safety. Other design elements, such as the duration of follow-up and the degree of detail in describing the exposure should be matched. We would agree that in a situation where multiple registries are operating in the same region, procedures should be established to identify individuals with overlapping participation, perhaps by developing data queries that search for records with matching elements such as name, date of birth, contact information, etc.

Minor comment on Table 2 to ensure consistent cell alignment throughout.

Thank you for your comment. The outcomes columns in the table are now left-justified, rather than centered.

Would it also be possible to add details on the list of countries if the registries/systems are "multinational" or "global", unless the list of countries is very expansive?

We agree that listing the countries covered by the global and multinational systems would be informative, their number makes it impractical to integrate into a table cell. We hope that the map provided in Figure 2 provides sufficient indication of the geographic coverage of these systems.

Lastly, what is the difference between blank cells and cells with "N/A"?

The maternal and infant outcomes were inadvertently deleted from the “Maternal conditions-based registries” group and have been restored. In addition, the first PER listing should have included the text “none specified” for the cell regarding maternal outcomes, and this has been restored as well.

Figure 1: It is not clear what the difference between some of the exclusion reasons are, such as "No pregnancy outcomes" vs. "Wrong outcomes", "Overview/summary/methods paper" vs. "Overview/summary paper", and "Wrong patient population" vs. "Pediatric population". Why was "wrong comparator" a reason for exclusion?

Thank you for highlighting the ambiguities in these categorizations, which were developed prospectively but not ultimately used as differentiators in our analyses. To clarify and streamline our exclusion categories, we have now consolidated them. Of note, the study excluded as a having the “wrong comparator” was a case-control study looking at the effect of maternal HIV status on pregnancy outcomes. This study was described in a conference abstract and while it may be assumed that all of the HIV positive women were on antiretroviral therapy and HIV negative were not, the analysis was based on HIV status rather than on the drug exposure, and so this reason for exclusion was given. This exclusion has now been included in the “wrong study design” category.

Figure 2 is a very nice visual representation. I would just advise making sure it is accessible to color-blindedness and the legend being inclusive (I don't think the red diagonal lines is in the legend unless I'm misunderstanding the legend).

[Response under development by WHO co-authors.]

Reviewer: 2
Dr. Neda SoleimanvandiAzar, Iran University of Medical Sciences
Comments to the Author:
This research was conducted as a Scoping Review on the Landscape Analysis of Pregnancy Exposure Registries in Low- and Middle-Income Countries. I thank the authors of the article for choosing this topic and writing this manuscript.
However, there are few points that it is recommended to be addressed as far as possible.
1- Title: The title was representative of the type of study.
2- Abstract: It was well written.
3- Introduction: It was well written.
4- Material and Methods: It was rather well written. Would the authors explain why the didn’t search in web of Science and Scopus?

Thank you for your question. At the time of designing our search strategy, we had believed that the Web of Science and Scopus would likely overlap considerably with our chosen data sources. However, we acknowledge that these repositories may have captured some unique references, and would certainly consider incorporating them for future reviews.

5- Results: It was well written.
6- Tables: They were Illustrative
7- Figures: They were Illustrative
8- Discussion: It was well written.

9- Limitation: The authors didn't mention the limitations of the study at the end of the discussion.

Thank you for this comment. As noted above, we have called out and expanded upon the review of limitations in our Discussion section.

10- Conclusion: It was well written, and it was according to the results.

Reviewer: 1
If you have selected 'Yes' above, please provide details of any competing interests.: Not applicable

Reviewer: 2
If you have selected 'Yes' above, please provide details of any competing interests.: Not applicable

---

VERSION 2 - AUTHOR RESPONSE

Reviewer 1:

First comment: How did the authors identify the key informants/experts, particularly what was the rationale for reaching out to individuals within the WHO PIDM or ESC? Were any other experts outside of these groups considered?

Original response:

PIDM members are the pharmacovigilance authorities for their respective countries, and are therefore expected to be knowledgeable about PER's in their area because they would likely be operators, stakeholders, and/or at least users or recipients of the data. We considered that it would be likely that teams that run PERs in a country would consult with national PV staff during the establishment or operation of the registries. The ESC is a group of experts in maternal immunization safety who are members of the following WHO advisory committees: Strategic Advisory Group of Experts on Immunization (SAGE), Advisory Committee on Safety of Medicinal Products (ACSoMP), Global Advisory Committee on Vaccine Safety (GACSV) and Mother and Newborn Information for Tracking Outcomes & Results. ESC members assessed and advised WHO on a number of topics managed by the pharmacovigilance team, including this study. Additional experts were identified to receive the survey based on the published and grey literature review as well as personal referrals. Additional language has been included in the manuscript.

Text added at lines 146-158 (this information had already been partially added)

Third comment: Were only sources in English considered or were other languages also assessed and reviewed?

Original response:

We used sources in languages available on the listed databases, which did include some Spanish and Portuguese. Abstracts and full-text were translated for data abstraction.

Text added at lines 134-136

Fifth comment: Regarding the first secondary question, "Can the PERs that have been identified in LMICs be used or adapted to monitor additional new vaccines or drugs that may be introduced for pregnant women?": how feasible or smoothly could new exposures be incorporated? The authors state most of the registries could be strengthened or modified, but what conditions would need to be met, and what obstacles exist in other registries identified that would prevent this?

Original response:

Criteria to determine whether the described system could be utilized or adapted for future PER include whether the system was currently operational, designed with flexibility to accommodate new vaccines or drugs, capable of timely identification of adverse events, employ electronic or mobile tools for data collection and reporting, incorporate mechanisms for follow-up and reminders, and had prior use in post-marketing safety surveillance.

The ease with which new exposures could be added can also vary based on the complexity of the exposure, such as if multiple doses are given, or if the drug or vaccine can be administered at different locations, or if additional verifications are needed.

Text added at lines 165-172

Sixth comment: Regarding the other secondary question: "What is the potential for data harmonisation and/or combining of data across databases and registries?": it is understandable more details of each of the registries would be needed to determine potential for harmonization, so what did the authors hope to be able to answer for this question? What are some considerations the authors think are needed to harmonize data? For example, if there are multiple registries in the same region, I'm wondering if it would be possible to identify overlapping individuals and how to account for that.

Original response:

One consideration would be whether the registry uses the standardized case definitions developed by the Brighton Collaboration and the GAIA initiative, as they can promote harmonization, enhance reproducibility, and ensure scientifically robust assessments of safety. Other design elements, such as the duration of follow-up and

the degree of detail in describing the exposure should be matched. We would agree that in a situation where multiple registries are operating in the same region, procedures should be established to identify individuals with overlapping participation, perhaps by developing data queries that search for records with matching elements such as name, date of birth, contact information, etc.

Text added at lines 418-425

- Please include your response to reviewer 2's fourth comment in the manuscript itself.

<u>Reviewer 2:</u>

Fourth comment: Material and Methods: It was rather well written. Would the authors explain why they didn't search in web of Science and Scopus?

Original response:

Thank you for your question. At the time of designing our search strategy, we had believed that the Web of Science and Scopus would likely overlap considerably with our chosen data sources. However, we acknowledge that these repositories may have captured some unique references, and would certainly consider incorporating them for future reviews.

Text added at lines 400-404
